# Population decline of the saguaro cactus throughout its distribution is associated with climate change

**DOI:** 10.1093/aob/mcae094

**Published:** 2024-06-08

**Authors:** Ricardo E Félix-Burruel, Eugenio Larios, Edgar J González, Alberto Búrquez

**Affiliations:** Posgrado en Ciencias de la Tierra, Instituto de Geología, Universidad Nacional Autónoma de Mexico, Mexico; Ecología para la Conservación del Gran Desierto, A.C., Hermosillo, Sonora, Mexico; Ecología para la Conservación del Gran Desierto, A.C., Hermosillo, Sonora, Mexico; Programa Educativo en Ecología, Universidad Estatal de Sonora, Hermosillo, Sonora, Mexico; Departamento de Ecología y Recursos Naturales, Facultad de Ciencias, Universidad Nacional Autónoma de México, Mexico City, Mexico; Instituto de Ecología, Universidad Nacional Autónoma de México, Hermosillo, Sonora, Mexico

**Keywords:** Climate change, population projections, drought, vital rates, environmental variation, species range, *Carnegiea gigantea*

## Abstract

**Background and Aims:**

Climate change is a global phenomenon affecting species, which in arid regions will translate into more frequent and intense periods of drought. The Sonoran Desert is becoming hotter and drier, and many organisms are rapidly changing in abundance and distribution. These population attributes depend directly on the dynamics of the population, which in turn depends on the vital rates of its individuals; yet few studies have documented the effects of climate change on the population dynamics of keystone species such as the saguaro cactus (*Carnegiea gigantea*). Although saguaros have traits that enable them to withstand present environmental conditions, climate change could make them vulnerable if forced beyond their tolerance limits.

**Methods:**

We evaluated the effect of climate change on 13 saguaro populations spanning most of the species’ distribution range. Using field data from 2014 to 2016, we built an integral projection model (IPM) describing the environmentally explicit dynamics of the populations. We used this IPM, along with projections of two climate change scenarios and one no-change scenario, to predict population sizes (*N*) and growth rates (*λ*) from 2017 to 2099 and compared these scenarios to demonstrate the effect of climate change on the future of saguaro cactuses.

**Key Results:**

We found that all populations will decline, mainly due to future increases in drought, mostly hindering recruitment. However, the decline will be different across populations, since those located near the coast will be affected by harsher drought events than those located further inland.

**Conclusions:**

Our study demonstrates that climate change and its associated increase in drought pose a significant threat to the saguaro cactus populations in the Sonoran Desert. Our findings indicate that the recruitment of saguaros, vital for establishing new individuals, is particularly vulnerable to intensifying drought conditions. Importantly, regional climate trends will have different impacts on saguaro populations across their distribution range.

## INTRODUCTION

Deserts and semi-deserts are the most extensive biomes on Earth, occupying more than one-third of the global land surface (Meigs, 1953; Cherlet *et al.,* 2018). These ecosystems are predicted to be highly vulnerable to global warming (IPCC, 2021). Increasing CO_2_, rising temperature, decreasing precipitation, and expanding variance in rainfall and temperature are changing community species compositions, ecosystem processes and biodiversity through their effects on population demography, risking the persistence of key species (Christensen and Lettenmaier, 2007; Seager *et al*., 2007; Overpeck, 2013). The Sonoran Desert is projected to experience increased aridity in the 21st century due to increasing temperatures and variability in mean annual precipitation (IPCC, 2021). Regional droughts will be the most common form of disturbance, producing gaps and pulses in population age distributions (births and deaths) (Swetnam and Betancourt, 1998). The consequences of the ongoing climatic changes on keystone species in the Sonoran Desert have been modelled using bioclimatic variables and species distribution models (Albuquerque *et al*., 2018; Zachmann *et al*., 2021; Brown *et al*., 2023). However, few studies have used demographic data such as growth, survival and fecundity to project population performance under climate change, which provide a mechanistic understanding of the processes driving population dynamics. Demographic data for long-lived desert species are scarce, with few long-term studies (Pierson *et al*., 2013; Rodriguez-Buritica *et al*., 2013; Orum *et al*., 2016; Winkler *et al*., 2018), and even fewer that measure the entire set of vital rates (growth, survival and fecundity) or cover a sizable portion of a species’ geographical range. These long-term demographic datasets could be important assets to inform how climatic variation affects the demography of key species, and how climate change will in turn determine their population viability in the future.

The saguaro cactus (*Carnegiea gigantea*) is a keystone and iconic species of the Sonoran Desert, whose distribution spans almost all of the Desert’s mainland extension (Yetman *et al*., 2020). Saguaros are particularly suitable plants for evaluating the effects of climate change because their growth and survival are mainly controlled by temperature and water availability (Steenbergh and Lowe, 1977; Pierson and Turner, 1998; Félix‐Burruel *et al*., 2019). More specifically, adult saguaros can persist well in the present desert environment and have fine-tuned their physiological responses to current environmental cues (Orum *et al*., 2016). However, under a rapid climate change scenario, the selective pressures acting on saguaro vital rates could become more intense. This is particularly important considering that saguaros may already exist close to their physiological limits in some parts of their range. This massive plant is known for its superb adaptation to extreme temperatures and extended periods of drought conditions that prevail in the Sonoran Desert. However, their seedlings are particularly vulnerable to drought (Winkler *et al*., 2018; Rodríguez‐Buriticá *et al*., 2019), and the regeneration niche envelope for seedlings (*sensu*Grubb, 1977) is very different from that of the adults. The result of this differential ontogenetic adaptation to desert conditions is a pattern of episodic recruitment of new individuals with multi-decadal periodicities potentially linked to the El Niño Southern Oscillation (ENSO) (Félix-Burruel *et al*., 2021). This phenomenon can be observed in the peculiar size and age distribution that saguaro populations possess: the species typically shows multimodal size/age distributions suggesting that recruitment is episodic, only happening during years when good conditions of low drought prevail (Steenbergh and Lowe, 1977; Pierson and Turner, 1998; Rodriguez-Buritica *et al*., 2013; Orum *et al*., 2016; Conver *et al*., 2017). Due to this vulnerable period in the life cycle of saguaro, it is still unknown whether the dynamics of saguaro populations will allow them to persist under the climate change conditions forecasted for the medium- and long-term.

Population models that have studied saguaro recruitment have typically measured the number of recruits by directly counting new individuals in long-term monitoring programmes, mostly around the area of Tucson, Arizona (Orum *et al*., 2016; Winkler *et al*., 2018; Rodríguez‐Buriticá *et al*., 2019). These studies have concluded that recruitment in saguaro populations is controlled by extended periods of suitable microsite conditions at the square metre scale or lower that include suitable nurse plant shade, ample humidity, and protection from herbivores and trampling animals. While direct counts are prime evidence of the recruitment process, they are labour-intensive, and usually only cover a small part of the saguaro range distribution, ignoring the whole suite of environmental variation experienced by the species.

While vital rates related to individual growth, survival and fecundity are often measured in typical monitoring programmes (those that measure height, survivorship and reproduction of individuals each year at each site), vital rates that occur between the seed and recruit stages are often neglected. This is because the fate of seeds from one year to the next is difficult to assess in the field. It is also difficult to observe newly germinated saguaro seedlings unless devoting high-intensity monitoring to find new seedlings. A way to circumvent these issues is to infer unobserved vital rates through inverse modelling (Evans *et al*., 2016; González *et al*., 2016). Inverse modelling of vital rates refers to the use of linear models that infer vital rate values as a function of size and environmental variables but in a decreasing fashion. For example, inverse growth models look at responses to decrements in size, rather than to increments, to model the decrease in size in the past. Inverse modelling has been used to decrease individual saguaros to estimate date of recruitment through a minimum size that represents the size-at-recruitment (1 cm in saguaros) (Félix-Burruel *et al*., 2021). Once date-of-recruitment is determined, the time series of recruitment events and the number of recruits can be inferred, and modelling as a function of the environmental variables can be performed. Previous studies have taken an indirect approach using a growth equation to estimate the year of recruitment of saguaros (Steenbergh and Lowe, 1977; Drezner and Balling, 2002; Drezner, 2006; Donnermeyer and Drezner, 2012; Rodríguez‐Buriticá *et al*., 2019; Félix-Burruel *et al*., 2021). These studies have all concluded that periods of saguaro regeneration are correlated with the temporal patterns of ENSO (usually 2–7 years).

Climate change has been recognized to be affecting saguaro populations through increased frequency of extreme events such as drought, flooding or freezing events (Rodríguez‐Buriticá *et al*., 2019; Swann *et al*., 2021). For example, the rate of establishment of saguaro seedlings slowed dramatically in the population at Saguaro National Park. Also, Swann *et al.* (2018) found that the establishment and growth of saguaros appear to be strongly influenced by drought and high temperatures in the northern region of the saguaro cactus distribution. Albuquerque *et al.* (2018), using bioclimatic variables, forecasted the impact of climate change on the distribution of saguaro for 2050 and 2070. The authors found that at least 8 % of the suitable habitat for the saguaro cactus would be lost by the influence of climate change by 2070. They also found that mean annual precipitation and maximum temperature of the warmest month were the most influential physical variables that will have an impact on the distribution. However, their study only used the influence of regional physical variables to forecast the impact of climate change on the saguaro distribution, ignoring the intrinsic processes that drive population growth, particularly the peculiar recruitment dynamics of this long-lived species.

The main aim of this study was to evaluate the response of the long-lived saguaro cactus to climate change in the context of spatially differentiated climatic conditions. We investigated the effect of climatic projections on the future of saguaro using field data to project the dynamics of 13 populations spanning its distribution range in Sonora, Mexico. Projections were done using an environmentally driven integral projection model (IPM) coupled with climatic projections of two climate change scenarios and a scenario of no climate change from 2016 to the end of the century. If climate change is affecting saguaro populations, we expect climatic projections under climate change to decouple from those derived from the no climate change scenario.

## MATERIALS AND METHODS

### Study species

The saguaro cactus, *Carnegiea gigantea* (Engelmann) Britton & Rose, is a signature species of the Sonoran Desert and one of the most studied perennial wild plant species (Yetman *et al*., 2020). This columnar cactus, only found in the Sonoran Desert, has a lifespan of up to 200 years under appropriate conditions (Pierson *et al*., 2013). Large, adult individuals reach heights ranging between 12 and 18 m and develop branches as they age (Niering *et al*., 1963; Steenbergh and Lowe, 1977). Saguaros tend to grow very slowly; for example, a 10-year-old plant is about 3–5 cm tall, and it needs to reach around 2.5 m to reproduce at an age of 30–40 years (Orum *et al*., 2016). The most critical physical factors for the growth of saguaros are water and temperature (Steenbergh and Lowe, 1977; Pierson and Turner, 1998; Félix‐Burruel *et al*., 2019).

Like many long-lived plants of the world’s deserts, mature saguaro cacti have physiological and morphological adaptations that allow them to survive long periods of moisture shortage and to take maximum advantage of short, infrequent wet periods. The seedling stage is critical for the long-term viability of saguaro populations because at this stage most organisms die from drought and, on higher ground or northern latitudes, frost. Significant saguaro recruitment requires several consecutive years of favourable rainfall periods (Steenbergh and Lowe, 1977; Jordan and Nobel, 1982; Turner, 1990; Winkler *et al*., 2018; Félix-Burruel *et al*., 2021) with sufficient soil moisture, mild winters, wetter than average years, and the presence of protective objects such as rocks or plants acting as nurses (Turner *et al*., 1966; Steenbergh and Lowe, 1977; Drezner and Balling, 2002; Munguía-Rosas and Sosa, 2008; Conver *et al*., 2017). In addition, individual growth rates decrease as organisms reach reproductive maturity because the allocation of resources is split between growth and reproductive effort (Steenbergh and Lowe, 1977).

### Study sites

We established 13 field sites with stands of saguaro across its distribution range in the state of Sonora, Mexico. Twelve sites cover the four mainland subdivisions of the Sonoran Desert, and one site is in the thornscrub transition between the Sonoran Desert and the tropical deciduous forest of the Pacific coast. Study sites are distributed between 26.8°N and 32.2°N, and 109.3°W and 114.1°W, roughly covering an area of 25 square degrees with elevations from almost sea level to 900 m.

Across the study area, the climate has a bimodal pattern of rainfall with convective precipitation during the summer monsoon (July–September) and cold frontal winter rains during the autumn–winter (November–March) (Búrquez *et al*., 1999; Phillips and Comus, 2000). Even at local scales, precipitation shows large seasonal and spatial variability. Overall, the climate is extreme, with yearly temperature maxima of more than 50 °C, brief winter freezes and irregular precipitation. Across the study sites, there is a strong northwest–southeast rainfall gradient. In the northwest, winter rains predominate, while in the southeast most of the rain is summer rainfall (Phillips and Comus, 2000).

### Demographic data

To consider saguaros of all sizes (including seedlings older than 1 year) at each site, we set quadrants of 20 × 20 m until the sample included over 100 living saguaros. Each site ended up being of a different size depending on saguaro local density. On each site, all individuals were identified with an ID number attached to the main trunk and geotagged. Also, to set a height measurement baseline to reduce re-measurement error in case of soil erosion, we also placed a permanent mark on the main stem of each saguaro. The permanent mark for saguaros over 2 m tall was placed at breast height (150 cm), and those lower than 2 m tall at 15 cm. In the case of seedlings, only the location and height were recorded. Individual size was defined as the sum of the height of the main stem and the lengths of all branches. Height measurements were taken from the ground to the apex positioning with a telescoping rod at the permanent mark of each saguaro and were recorded to the nearest centimetre (millimetre in the case of seedlings). Saguaros taller than 2 m were measured using a centimetre graduated telescoping rod with a terminal crossbar (Crain Enterprises, Inc., Mound City, IL, USA). Smaller saguaros were measured with a tape measure. The length of each branch resulted from the subtraction of the length from the ground to the branch base from the length from the ground to the apex of the branch. Further details on the collection of these data are described in Félix‐Burruel *et al*. (2019) and Félix-Burruel *et al*. (2021). Individual growth and survival were measured in the winter seasons (December–February) of 2014, 2015 and 2016. During these seasons, the length of all branches of each plant was measured.

### Environmental variables

We assembled a set of ecologically meaningful environmental variables, varying at the local (site) and regional (Sonoran Desert) scales, identified as the main determinants of saguaro cactus vital rates (Turner *et al*., 1966; Pierson and Turner, 1998; Drezner and Balling, 2002; Orum *et al*., 2016; Winkler *et al*., 2018; Rodríguez‐Buriticá *et al*., 2019; Félix‐Burruel *et al*., 2021). We used two representative variables that operate at the regional scale, the tropical surface water temperature (TSW) and the Palmer Drought Severity Index (PDSI). Also, we included one local-scale variable, the soil water content at saturation (SWC) that operates at the individual level within each population.

By considering both the local and regional scales, we gained a comprehensive understanding of how demographic processes operate within individual populations as well as how they interact and contribute to population dynamics at a larger spatial scale.

#### Tropical surface water temperature

TSW is one of the ways to determine whether the global climate is experiencing an ENSO state. ENSO is a recurring climate pattern in the equatorial Pacific well known for its influence on climate extremes (in temperature and rainfall) all over the world. ENSO events are responsible for wet (warm phase: El Niño) and dry (cold phase: La Niña) conditions associated with precipitation anomalies in the Sonoran Desert (Salinas-Zavala *et al*., 2002). In the warm phase of ENSO, unusually wet conditions are typical during winter and early spring (Salinas-Zavala *et al*., 2002; Magaña *et al*., 2003; Zolotokrylin *et al*., 2016).

In this study, we used forecasted climate values from the Ensemble Simulations of Extreme Weather Events Under Nonlinear Climate Change (ESSENCE) project (Sterl *et al*., 2007). We used NINO 3.4 index annual values (TSWs averaged over the NINO3 and NINO4 regions 5N-5S, 170W-120W) (Bamston *et al*., 1997), from 1950 to 2100, as a driver of saguaro recruitment (Félix-Burruel *et al.*, 2021). We downloaded these data from the Climate Explorer website (http://climexp.knmi.nl), provided by the Koninklijk Nederlands Meteorologisch Instituut (KNMI). We averaged the annual TSW among 17 modelling groups available. Additionally, we used this variable lagged 1 year from the year of the demographic census. This allowed us to consider vital rates not responding immediately to weather cues.

#### Palmer Drought Severity Index

PDSI is a simple model to calculate environmental water stress from the cumulative imbalance of soil moisture supply (Palmer, 1965). This model only requires precipitation, temperature and potential evapotranspiration from observed, reconstructed or projected data. Wells *et al*. (2004) proposed a self-calibrating PDSI (scPDSI) which calibrates the PDSI at any location by replacing empirical constants in the index computation with dynamically calculated values. We obtained scPDSI data from 1950 to 2017 (past period) using the KNMI Climate Explorer data (http://climexp.knmi.nl; van der Schrier *et al*., 2013; Barichivich, 2018). The model ensemble output at the KNMI website is provided on three resolutions; we present our analysis at 0.5° resolution. To estimate future scPDSI values, we used bias-corrected and downscaled monthly temperature and precipitation data generated by the Coupled Model Intercomparison Project Phase 5 (CMIP5; Taylor *et al*., 2012). We analysed the period of 2017–2100 data for the greenhouse gas emission scenarios or representative concentration pathways (RCPs) 2.6 and 8.5 future concentrations expected for 2100 (IPCC, 2014). The monthly mean of temperature and precipitation variables were averaged to produce yearly variables for all modelling groups. We calculated the monthly scPDSI using the scPDSI package (Palmer, 1965; Wells *et al.*, 2004) in R (R Development Core Team, 2021) to obtain annual scPDSI values for each site by averaging its yearly values. Additionally, we also included a one-year delay of the scPDSI data as a variable.

#### Soil water content at saturation

For SWC, we followed the procedure described in Félix-Burruel *et al*. (2021). We used the van Genuchten function to estimate soil water content at saturation (*w*) at a particular point in the water retention curve from soil textural data for each saguaro (van Genuchten, 1980):


$$\begin{array}{l} \begin{array}{lllll} w= & \;52.753\;+\;0.666\cdot Si+\;0.157\cdot Sa-0.008\cdot Si^{2}\;\; & -12.311/Sa\;-6.476\cdot ln\left(Sa\right)-0.004\cdot Cl\cdot Si\; & +\;0.004\cdot Cl\cdot Sa\;-0.004\cdot Si\cdot Sa,\;\; & where\;Sa=\;Sand,Cl=\;Clay\;and\;Si=Silt.\; \end{array} \end{array}$$

Soil data from 15 randomly distributed samples at 0–15 cm depth across each site were processed and analysed with the Bouyoucos method to determine sand, silt and clay percentages (Bouyoucos, 1962). To assign a soil texture to each plant, we interpolated the values for soil particle fractions of sand, silt and clay from each site using inverse distance weighting (Shepard, 1968). The soil texture analysis was conducted at Departamento de Ingeniería Química y Metalurgia, Universidad de Sonora, Hermosillo, Sonora, Mexico.

### Modelling population dynamics

#### Integral projection model

An IPM allows the incorporation of the effect of continuous individual traits such as size and environmental variables on the dynamics of a population. This procedure allows for an estimation of the population growth rate (Easterling *et al*., 2000; Ellner *et al*., 2016). Also, the IPM is a useful tool to project future population dynamics to evaluate a species response under different scenarios (Merow *et al*., 2014; González *et al*., 2021). The function (kernel) describing the dynamics of the saguaro was


$$\begin{array}{l} k\left(z,z^{\prime },p_{t},p_{t-1},w\right)=\;s\left(z,\;p_{t},p_{t-1},w\right)\cdot g\left(z,z^{\prime },p_{t},p_{t-1},w\right), \end{array}$$ (1)

where *s* describes the probability of an individual of size *z* to survive from time *t* to *t* + 1 under environmental conditions *p*_*t*_ (PDSI), *p*_*t*–1_ (PDSI 1-year lag, i.e. PDSI at time *t* − 1) and *w* (SWC); and *g* is the probability of a surviving individual of size *z* to change at time *t* + 1 to a size *z*ʹ under *p*, *p*_*t*–1_ and *w* environmental conditions. In this study, size was modelled as the standardized natural log of individual size (*z*) in centimetres (at time *t*).

To assess the current effect of the environment on the demography of saguaro, we determined the population structure for each site through the integral equation:


$$\begin{array}{lll} {n_{t}}_{+1}\left(z^{\prime }\right)\;= & \;\int k\left(z,z^{\prime },p_{t},p_{t-1},w_{t},e_{t}\right)\cdot n_{t}\left(z\right)dz\;\; & +\;r\left(\;p_{t},p_{t-1},w_{t},e_{t},e_{t-1}\right)\cdot c\left(z^{\prime }\right), \end{array}$$ (2)

where *n*_*t*_ is the size structure of the population at time *t*; *r* is the number of recruits produced at time *t* + 1 under a *p*, *p*_*t*–1_, *w*, *e* (TSW) and *e*_*t*–1_ (TSW 1-year lag) environment; and *c* is the size distribution of recruits. Note that we assume that recruitment does not depend on population size, but rather on the existence of favourable climatic conditions for this process to occur.

The parameterization of the vital rate functions composing the IPM (i.e. survival, growth, probability of recruitment and number of recruits) is discussed in the following sections.

#### Growth and survival

**Fig. 1.**
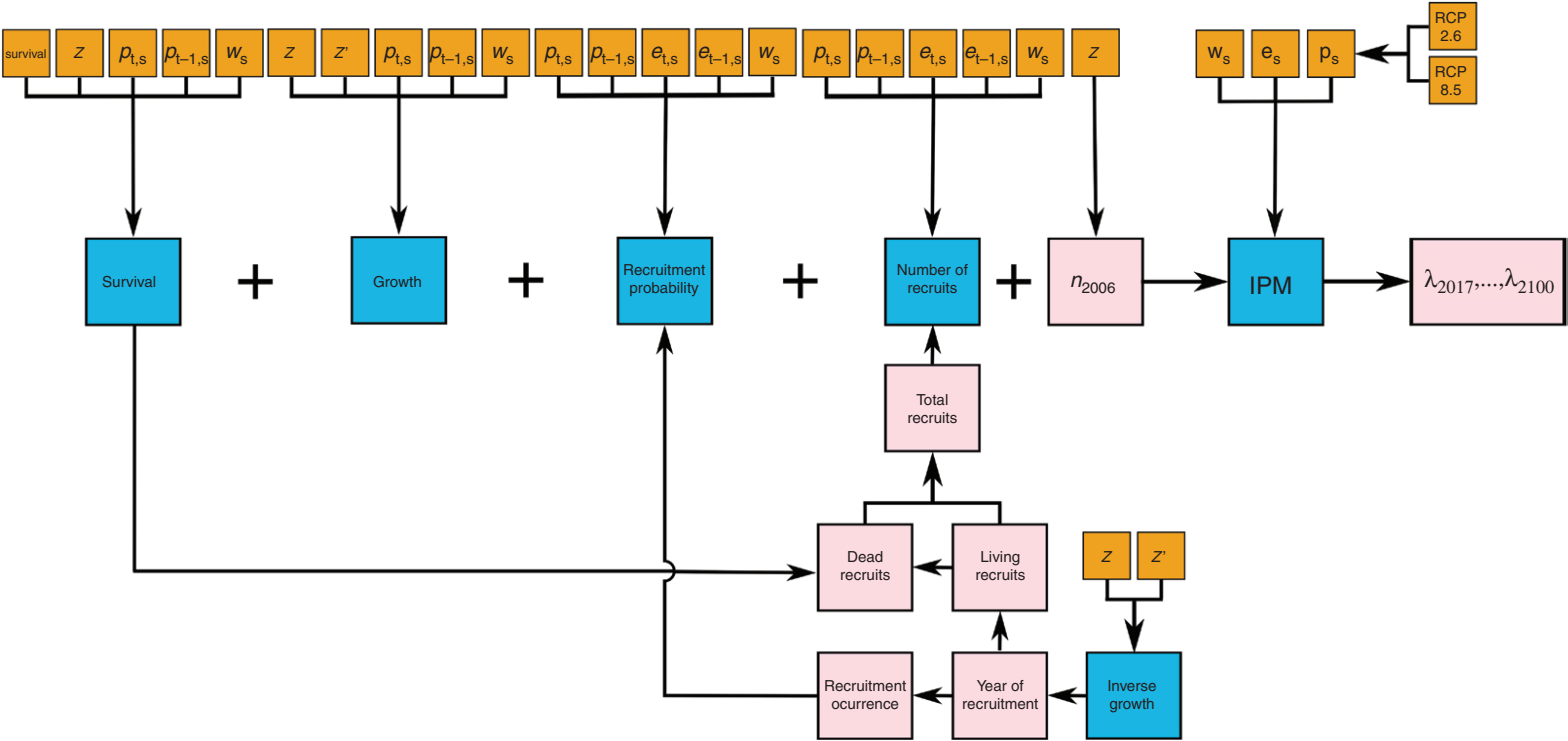
Modelling scheme describing the construction of the vital rate functions used in the integral projection model. The colours of the boxes denote the data (orange; variable names are provided in the main text), the vital rate functions (blue) modelled using the data and the estimates (pink) derived from these functions.

We fitted generalized linear and additive mixed models (GLMMs and GAMMs, respectively) to evaluate the effect of environmental factors on survival and growth of the saguaro cactus (Fig. 1). Survival was modelled with a binomial distribution while growth was assessed using a Gaussian distribution. We also considered the three environmental factors described above, *p*_*t*_, *p*_*t*–1_ and *w*, as explanatory (fixed) variables and site (*S*) as aq random effect. In our modelling procedure, we explored all possible combinations of explanatory variables, excluding those models that did not include the state variable as such variable is required in an IPM; we also considered all possible random effect structures.

To construct predictive models, a model selection was performed through a 5-fold cross-validation procedure (Dormann *et al*., 2018). The original dataset was randomly split into two groups: an 80 % train subset and 20 % test subset. Models were fitted with the training subset and predictions were made on the test subset. Prediction accuracy was evaluated through the square root of the mean squared error (RMSE). This random splitting was performed 100 times, and a mean RMSE (MRMSE), describing the average predictive power of each model was calculated. Models were ordered by their MRSME and an MRSME weight was calculated. Finally, an average model was constructed including those models that jointly accumulated 95 % of the MRSME weights.

#### Recruitment

To estimate the number of recruits per year and population, we first determined the year of recruitment from living individuals with an inverse growth model (Félix-Burruel *et al*., 2021), and then modelled the year of recruitment of dead saguaros (Fig. 1).

As with the growth model, the construction of an inverse growth model involved the fitting of GLMMs and GAMMs, in which we modelled the state variable observed at time *t* + 1 (*z*ʹ) as a function of the state variable at time *t*(*z*), with site as a random effect. An average predictive inverse-growth model was constructed also with a 5-fold cross-validation procedure as described in the previous section. With this average model, we estimated the year of recruitment of living saguaros, and then the events of recruitment (recruitment occurrence) as described in Félix-Burruel *et al.* (2021). To assess the year of recruitment of dead saguaros, we first predicted the estimated number of dead individuals that each living organism in each year of its life represented. We did this by estimating the annual probability of mortality of every individual; then, we added these probabilities as dead (non-observed) individuals, associating them with the year of recruitment of the living organisms. With the years of recruitment of all recruits (living and dead), we estimated the number of recruits per year per site. Since the survival function provides continuous probability estimates, the number of recruits was also a continuous variable.

Finally, we modelled, using GLMMs and GAMMs, the average recruitment probability and the total number of recruits (living and dead) as a function of *p*_*t*_, *p*_*t*–1_, *e*_*t*_, *e*_*t*–1_ and *w* as fixed effects, and using site as a random effect. Average recruitment probability was modelled with a binomial distribution and total number of recruits with a log-Gaussian distribution. Model selection was performed as in the previous section.

Model fitting of the survival, growth, inverse growth, recruitment probability and number of recruits was done in R (R Development Core Team, 2021) using the gamm4 package (Wood and Scheipl, 2020).

### Population dynamics projections to climate future scenarios

We used the IPM developed in the previous sections, the future TSW scenarios from the ESSENCE project (Sterl *et al*., 2007; Supplementary Data Fig. S1), and the future scPDSI calculated by the bias-corrected and downscaled climate variables generated by CMIP5 for RCPs 2.6 and 8.5 (Taylor *et al*., 2012; Fig. S1) to construct a time series of future population growth rates and estimated population sizes for our 13 saguaro populations from 2017 to 2099. We explored the influence of climate change by comparing the population dynamics of the saguaro cactus under the two future scenarios of climate change with the dynamics under a null scenario of no climate change. The null scenario included detrended climate projections from the RCP 8.5 scenario that conserved the natural variability and periodicity of the climate variables but excluded the trend of climate change. This detrending was performed by fitting a spline to the climate variable time series and using the residuals as the detrended variables (Fig. S2).

## RESULTS

### Modelling population dynamics and vital rates

#### Survival and growth

For survival probability, we averaged a total of 121 models out of 156 models constructed since MRMSE was similar for most fitted models (23.07–23.58 %; Supplementary Data Table S1). Average survival probability showed a rapid increase as a function of size; from seedlings to established individuals, with a relatively stable survival rate in this latter group (Fig. S2). Mean deviation from the population-specific survival patterns to the pooled population value was 0.84 %. The average probability of survival as a function of both PDSI and the delayed PDSI showed a constant pattern with a slightly negative effect and a mean deviation from the population-specific survival patterns to the average of all populations of 1.21 %. Finally, the average probability of survival showed a slight increase with increasing values of soil water content at saturation, and a 1.40 % mean deviation of the population-specific survival patterns from the average.

For growth, we constructed 264 models. For this vital rate, we averaged a total of 228 models because MRMSE was also similar in most fitted models, ranging from 0.069 to 0.073 cm year^−1^ (Supplementary Data Table S2). The average size at time *t* + 1 showed a positive relation with size at time *t* (Fig. S2). Mean deviation from the population-specific growth patterns to the averaged value was 12.27 cm year^−1^. Growth was not associated with neither PDSI nor the delayed PDSI (mean deviations of 1.01 and 1.02 cm year^−1^, respectively) or soil water content at saturation (mean deviation of 1.02 cm year^−1^) as shown by the lack of correlation from the average model.

#### Recruitment

For the estimation of recruitment, we first required an inverse growth model (Félix-Burruel *et al.*, 2021). We averaged five models that had MRMSE ranging from 0.069 to 0.071 cm (see Supplementary Data Table S3). The mean deviation from the population-specific growth patterns to the averaged values was 13.063 cm.

Recruitment probability was estimated by averaging 438 models out of 480 models built, which ranged in MRMSE from 0.466 to 0.477 (see Supplementary Data Table S4). The average probability of recruitment decreased with increasing TSW and with the delayed TWS (Fig. S3). This pattern suggests that as equatorial surface temperatures increased, the probability of recruitment decreased. For TSW, the mean deviation from the population-specific recruitment patterns to the averaged one was 2.822 %. Moreover, the average probability of recruitment increased slightly as PDSI and the delayed PDSI increased, suggesting that more new individuals were incorporated into the population under wetter conditions. The mean deviation from the population-specific recruitment patterns to the average was 3.004 %. Similarly, the probability of recruitment increased with higher soil water at saturation, suggesting that the volumetric water content in soils with more clay is beneficial for the recruitment of new individuals. The mean deviation from the population-specific recruitment patterns to the averaged estimates was 3.218 %. Finally, the probability of recruitment had a slight decrease from 1950 to 2016.

From the 452 models constructed, the estimation of the number of recruits was the result of the average of 33 models whose MRMSE ranged between 17.836 and 17.925 (see Supplementary Data Table S5). The number of recruits was a monotonic decay function of TSW (Fig. S3). The number of recruits increased as both PDSI and delayed PDSI increased, suggesting that wetter periods are favourable for recruitment and the number of recruits was somewhat higher at intermediate levels of soil water content at saturation. Finally, the number of recruits had a slight decrease in time from 1950 to 2016. Note that in these models no population-specific random effects could be determined.

### Population dynamics under two climate change scenarios and a null scenario

#### Projected vital rates

**Fig. 2.**
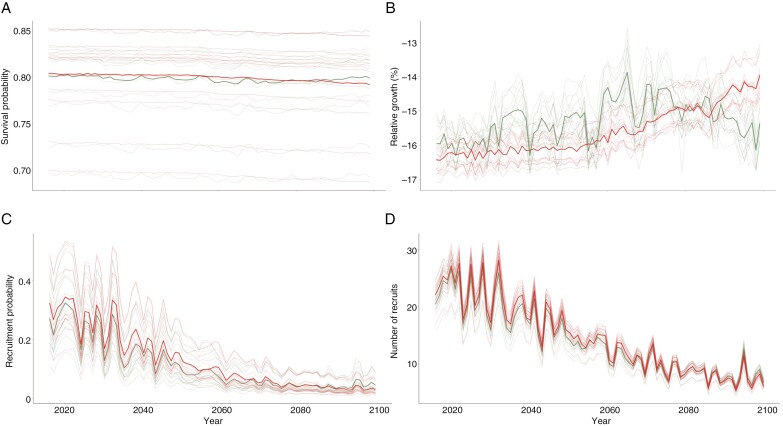
Time series of projected environmental drivers and vital rates under climate change: (A) mean survival probability, (B) mean relative growth, (C) recruitment probability and (D) number of recruits. Main estimates are shown by thick lines and population-specific estimates by thin lines.

In our projections for vital rates, we found a relatively constant probability of survival under very low (RCP 2.6) and very high (RCP 8.5) emission scenarios (Fig. 2A). We also observed a positive trend with more variation in relative growth for low emission (RCP 2.6) than for very high emission scenarios (RCP 8.5), and a negative trend for the low emission forecasts for the last two decades (Fig. 2B). The probability of recruitment and number of recruits decreased through time in both projections (Fig. 2C, D). Both scenarios show a similar periodic trend, with years of low recruitment followed by years of high recruitment. The first decade seems to have a stable trend with high variability; nevertheless, in the second decade, the number of recruits decreased showing less variability.

#### Projected population sizes and growth rates

**Fig. 3.**
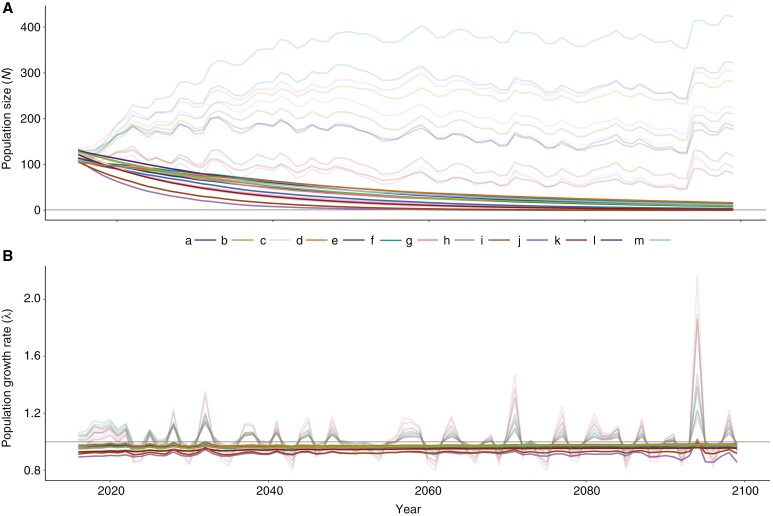
Time series of projected population sizes (*N*; ind.) and growth rates (*λ*; ind. year^−1^) of 13 saguaro populations (a–m) from 2017 to 2099 under a climate change scenario (solid lines) and a no climate change scenario (light lines). Populations: (a) Joyita, (b) Vidrios, (c) McDougal, (d) Primavera, (e) Caborca, (f) Cucurpe, (g) Dipo, (h) Lobos, (i) Orégano, (j) Bahía de Kino, (k) San Marcial, (l) Guásimas and (m) Masiaca.

Under a very high emission climate change scenario, all 13 populations showed declining population sizes (Fig. 3A) and negative growth rates (Fig. 3B) from 2016 to 2099, with this scenario displaying the mean lowest rates in comparison with the very low emission scenario (Supplementary Data Table S6 and Fig. S4). However, under the no climate change scenario, all populations showed stable population sizes and growth rates that fluctuated around 1, evidencing the negative effect that climate change will pose on saguaro populations.

**Fig. 4.**
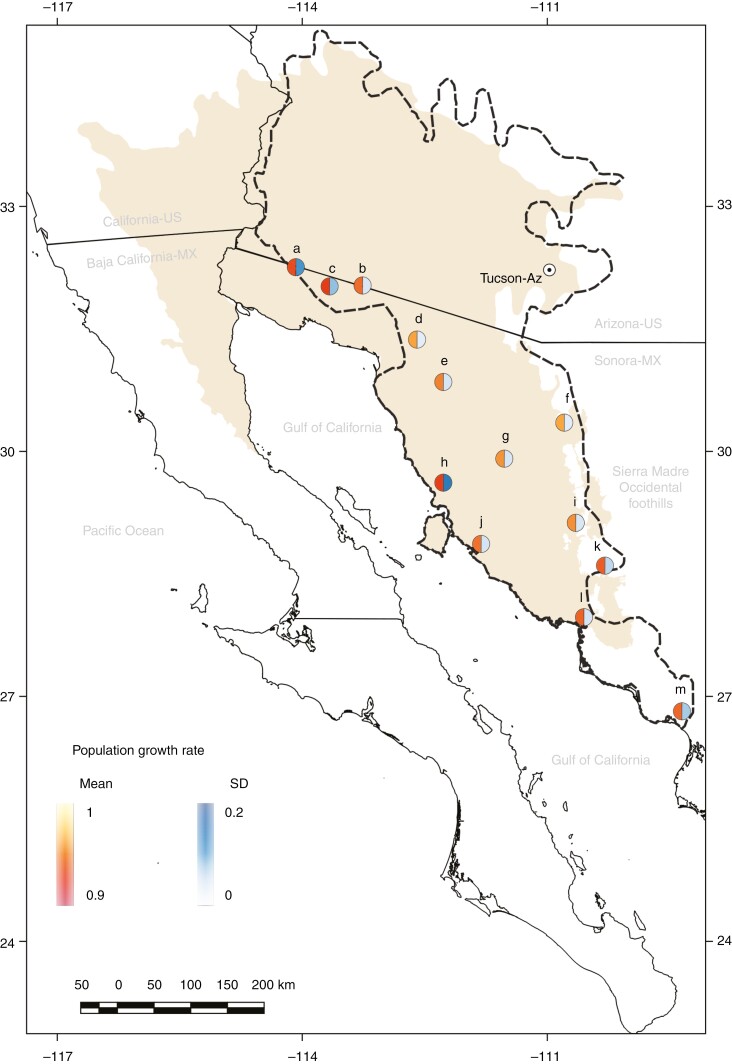
Sonoran Desert ecoregion (in light brown; Olson *et al.*, 2001), distribution of the Saguaro (dashed line) and location of the 13 study populations (a–m). Circles denote the mean (right half) and standard deviation (left half) of the population growth rates (ind. year^−1^) projected from 2017 to 2099 under the very high CO_2_ scenario. Populations: (a) Joyita, (b) Vidrios, (c) McDougal, (d) Primavera, (e) Caborca, (f) Cucurpe, (g) Dipo, (h) Lobos, (i) Orégano, (j) Bahía de Kino, (k) San Marcial, (l) Guásimas and (m) Masiaca.

Remarkably, the populations displaying the lowest growth rates were also the ones with the largest variations over the projected period (Fig. 4; Supplementary Data Table S6, columns ‘SD’). The most vulnerable populations are located near the coast of the Gulf of California while the populations near the Sierra Madre Occidental foothills, although at risk, are declining more slowly (Fig. 4). Furthermore, the rates of population decline do not change over time as indicated by their linear tendencies (Table S6 columns ‘Slope’). As a result, all populations show a declining pattern under the two climate change scenarios. If these conditions persist, these populations will face local extinction.

## DISCUSSION

Cacti are facing serious threats from climate change, especially in areas where hotter and drier conditions are becoming the norm (Goettsch *et al*., 2015; Pillet *et al*., 2022). However, the most accepted hypothesis from plant physiology is that species with CAM pathways are expected to persist in the drylands under future climate scenarios (Nobel, 1996). Moreover, it is important to emphasize that most studies of how climate change will affect cactus populations are based on presence/absence data and only a few incorporate demographic data or combine both (Pillet *et al*., 2022).

In this study, we provide the most comprehensive and geographically distributed assessment of projected saguaro cactus population dynamics under climate change yet attempted. The pattern of future saguaro population dynamics suggests that the projected climatic changes for the present century will have negative effects on the status of saguaro populations, especially when we compare them with the projected null scenario where there is no climate change (Fig. 3). Those populations exhibiting the largest effects are also those displaying the largest variation in these effects over time. An increase in variation in population size increases the likelihood of the population reaching a minimum size extinction threshold (Vucetich *et al*., 2000; Legendre *et al*., 2008).

Although saguaros are well adapted to the extreme conditions of the Sonoran Desert, most vital rates will be affected by changing future climate conditions (Fig. 2). In particular, recruitment will be most impacted through a reduction in the probability of recruitment and the abundance of recruits (Fig. 2C), partially because of the limited water storage capacity and shallow root systems of younger saguaros (Turner *et al*., 1966; Jordan and Nobel, 1982; Orum *et al*., 2016) that do not allow them to cope with drought (Winkler *et al*., 2018) – i.e. the regeneration niche widely differs between the recruit and the adult stages (Grubb, 1977). As established by Félix-Burruel *et al.* (2021) recruitment is periodic, potentially explained by the occurrence of El Niño conditions, and this periodicity will persist under climate change (Fig. 2C, D). In turn, survival, although slightly affected by increasing drought conditions (Fig. 2A), would be expected to translate into lower λ values under small increases in drought due to its high relevance for population growth (Godínez–Álvarez *et al*., 2003). Thus, for both vital rates, reduction in water availability represents the most significant threat to saguaros, particularly in those regions that are experiencing more frequent and intense droughts such as the Sonoran Desert. Finally, our modelling shows that under both climate change scenarios, growth declines (Fig. 2B); this finding can be ascribed to either a loss of branches and/or to slowed individual growth rates.

These patterns in the response of vital rates to climate change explain the expected declining population sizes and therefore the negative population growth rates of the saguaro in this century (Figs 2 and 3). This was true when we compared the two RCP scenarios with the null scenario (in which we eliminated the trend of climatic variables under climate change), for all saguaro populations. Our results also indicate that the eastern and central saguaro populations (near the Sierra Madre Occidental) will decrease their population sizes more slowly (mean in Fig. 4), and less variably (SD in Fig. 4), relative to those near the coast of the Gulf of California. This is in part consistent with what Albuquerque *et al*. (2018) found using species distribution models, where they predicted habitat suitability of the entire saguaro distribution range. They determined a reduction in habitat suitability for the western part of Sonora and an improvement in the western part of Arizona. However, they also determined that the rest of the saguaro distribution would display a relative maintenance of its suitability (no change from present suitability values), while in our case all populations decline, although at less negative rates. The main difference with the study of Albuquerque *et al*. (2018) is the inclusion of demographic data, i.e. while distribution models connect environmental data with species presence, our model links environmental variables with the vital processes that individuals followed through their lives, which in turn determine population abundance and, ultimately, species presence. This is a more mechanistic approach to determining future population trends (Hilborn and Mangel, 1997). However, in terms of the climate variables that were detected as having the largest effects on the species future status, Albuquerque *et al*. (2018) and us coincide. In both studies, precipitation, or the lack of it (i.e. drought), was the main factor determining habitat suitability and future population growth; in our case, this was by affecting the recruitment dynamics. Thus, aside from the responses to environmental factors, the findings of the present study emphasize the importance of incorporating population dynamics into spatial modelling to understand the mechanisms explaining the decline of a long-lived species such as the saguaro. The differential impact of future climate conditions on coastal saguaro populations, leading to faster declines in their population sizes compared to other saguaro populations, is worrisome. This concern is heightened by the fact that Sonoran coastal populations exhibit the highest genetic diversity, especially when compared to those at the northern edge of the range (Sanderson *et al*., 2022).

As climate continues to change, it is a challenge for ecologists and conservationists to understand and forecast the effects of climate change on plant populations. Furthermore, we must consider that different regions will experience local variations in temperature, rainfall patterns and other environmental factors such as drought due to climate change (IPCC, 2021). Therefore, populations of the same species may not respond to climate change in the same way, and thus it is imperative to understand the extent of spatial variation of these changes. Despite these challenges, the impacts of climate change on cacti have been largely unexplored, but recent research aims to shed light on the subject (Pillet *et al*., 2022). In this study, we link large-scale population data and climatic conditions. This information allows us to develop models to explain the relationship between vital rates and climate, and also forecast population responses in the future using climate projections.

Even though there is still much to be learnt about the effects of climate change on long-lived plant populations, our study addresses the challenges posed by climate change on long-lived saguaro populations. In this sense, although all saguaro populations in this study show future decline, we find that the rate of this decline varies mostly because of the region’s uneven distribution of current and future drought, revealing crucial information about its local extinction patterns in the future.

Saguaro is a highly resilient species, probably more so than the short-term conditions we studied suggest. They have endured past climate extremes during the last glaciations and have persisted. However, this is the first time in which anthropogenically driven climate and land-use change have been shown to affect saguaro and other long-lived species populations.

Research on the effects of climate change on keystone species is essential to enhance our understanding of their adaptive capabilities and their resilience to changing environmental conditions. By studying the responses of the saguaro across its distribution, we were able to identify those populations most viable under a changing future and those towards which conservation efforts should be directed.

## SUPPLEMENTARY DATA

Supplementary data are available at *Annals of Botany* online and consist of the following.

Table S1: Best predictive models used in the construction of the survival average model. Table S2: Best predictive models used in the construction of the growth average model. Table S3: Best predictive models used in the construction of the inverse growth average model. Table S4: Best predictive models used in the construction of the probability of recruitment average model. Table S5: Best predictive models used in the construction of the number of recruits average model. Table S6: Statistics of the growth rate time series projected for 13 saguaro populations from 2017 to 2099 under two climate change scenarios.

Figure S1. Estimates of El Niño Southern Oscillation and Palmer Drought Severity Index under a climate change and one no climate change scenarios. Figure S2. Average of survival probability and growth for the 13 saguaro populations. Figure S3. Recruitment probability and number of recruits for the 13 saguaro populations. Figure S4. Time series of projected population sizes and growth rates of 13 saguaro populations from 2017 to 2099 under two climate change scenarios.

mcae094_suppl_Supplementary_Figures_S1-S4

mcae094_suppl_Supplementary_Tables_S1-S6
