# Supplementary material for: Population decline of the saguaro cactus throughout its distribution is associated with climate change
Source: Ann Bot. 2024 Jun 8;135(1-2):317–28. doi: 10.1093/aob/mcae094 (PMC11805942; doi:10.1093/aob/mcae094)

**Title:** Population decline of the saguaro cactus throughout its distribution is associated with climate change.

Authors: Ricardo E. Félix-Burruel, Eugenio Larios, Edgar J. González, and Alberto Búrquez

**This supplementary document includes:**

**Figure S1.** Estimates of El Niño Southern Oscillation and Palmer Drought Severity Index under a climate change and a no climate change scenarios.

**Figure S2.** Average of survival probability and growth for the 13 saguaro populations.

**Figure S3.** Recruitment probability and number of recruits for the 13 saguaro populations.

**Figure S4.** Time series of projected population sizes and growth rates of 13 saguaro populations from 2017 to 2099 under two climate change scenarios.

**Figure S1.** Estimates of El Niño Southern Oscillation (as surface water temperature) under a climate change scenario (solid) and a no climate change scenario (dashed), and Palmer Drought Severity Index under two climate change scenarios, very low (solid green) and very high (solid red) CO_2_ emissions, and no climate change scenarios (dashed green and red), from 1950 to 2099. Patterns prior to 2017 are in light color and after 2017 are in solid color.


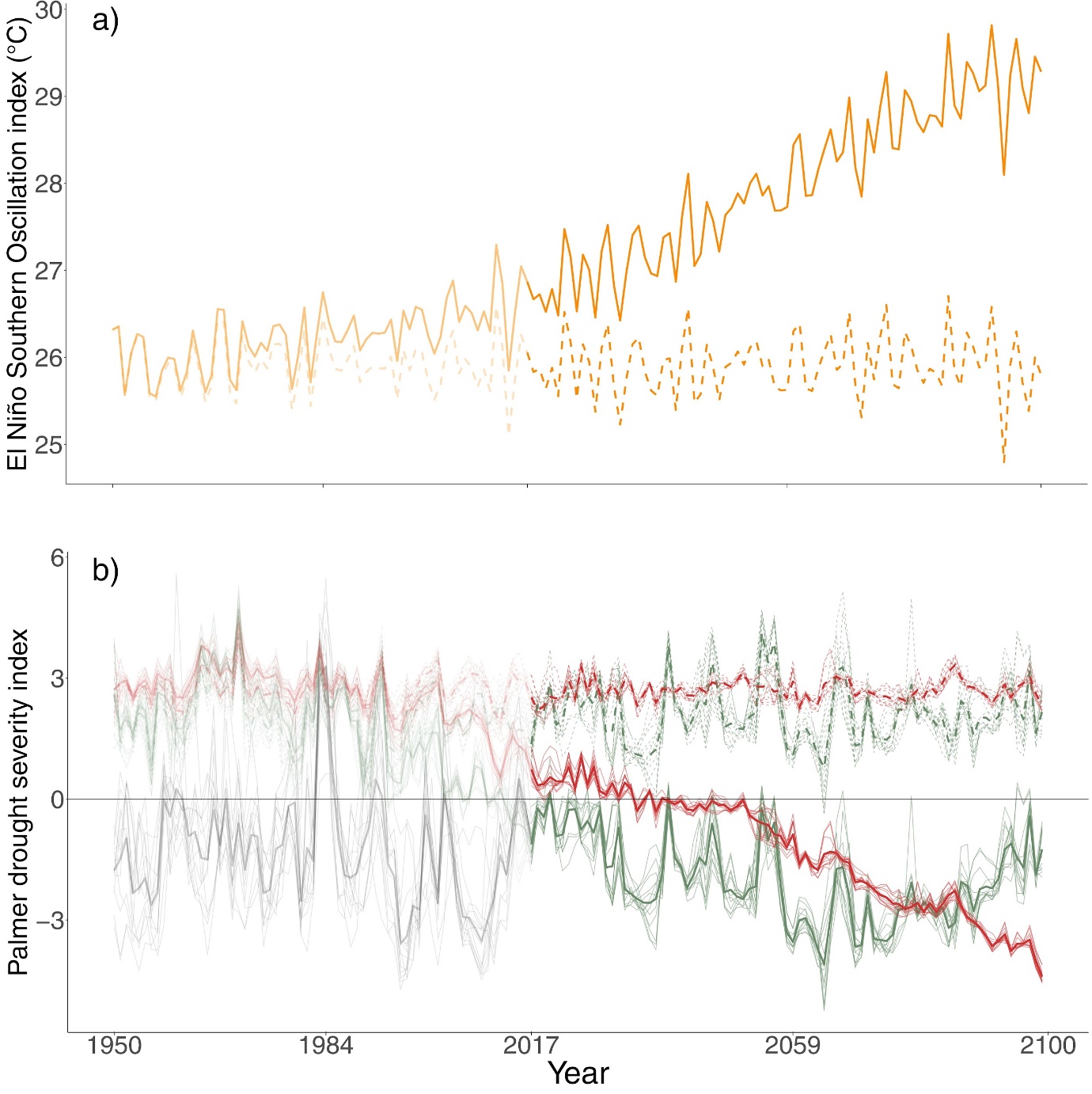


**Figure S2.** Average of survival probability and growth for 13 saguaro populations. Survival probability and growth were modelled as a function of individual size, soil water content at saturation and the Palmer Drought Severity Index and Palmer Drought Severity Index delayed.


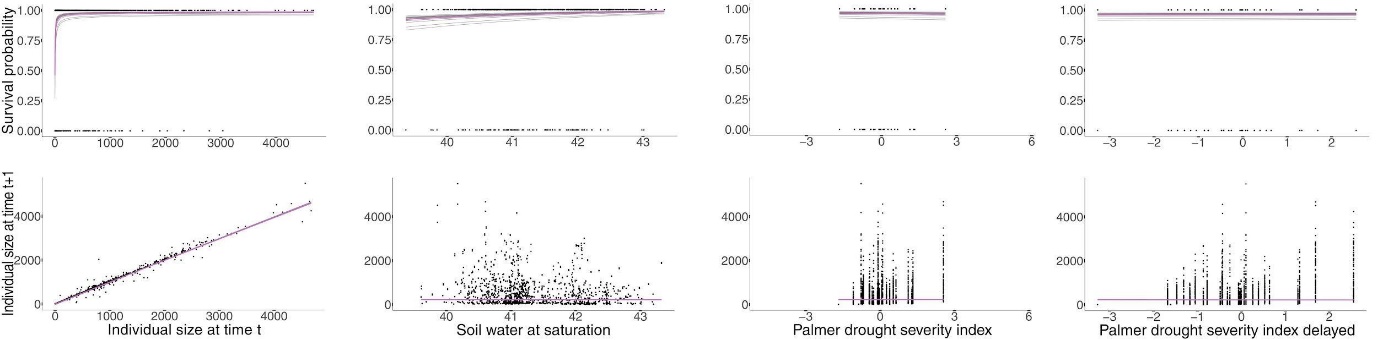


**Figure S3.** Recruitment probability and number of recruits for the 13 saguaro populations. Recruitment probability and the number of recruits were modelled as a function of soil water content at saturation, Palmer Drought Severity Index and Palmer Drought Severity Index Delayed, El Niño Southern Oscillation and El Niño Southern Oscillation Delayed.


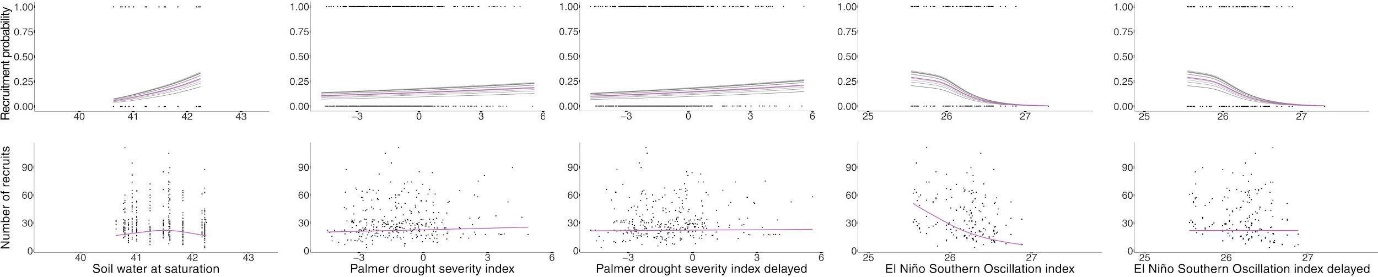


**Figure S4.** Time series of projected population sizes (*N*; ind) and growth rates (*λ*; ind.∙y^-1^) of 13 saguaro populations (a–m) from 2017 to 2099 under two climate change scenarios: very low (green) and very high (red) CO_2_ emissions. The gray line corresponds to a stable population (*λ* = 1). Population names are: a) Joyita, b) Vidrios, c) McDougal, d) Primavera, e) Caborca, f) Cucurpe, g) Dipo, h) Lobos, i) Orégano, j) Bahía de Kino, k) San Marcial, l) Guásimas and m) Masiaca.


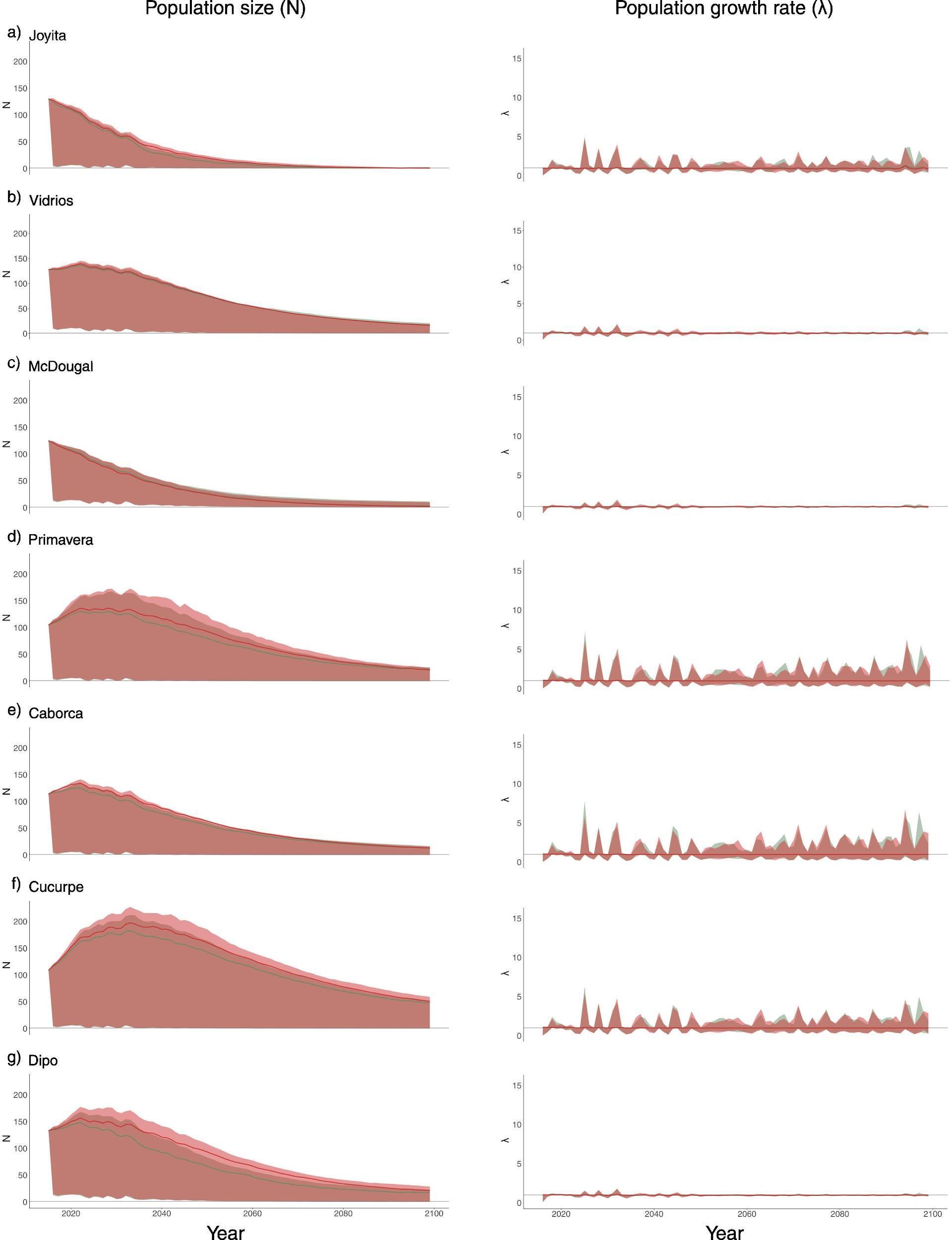


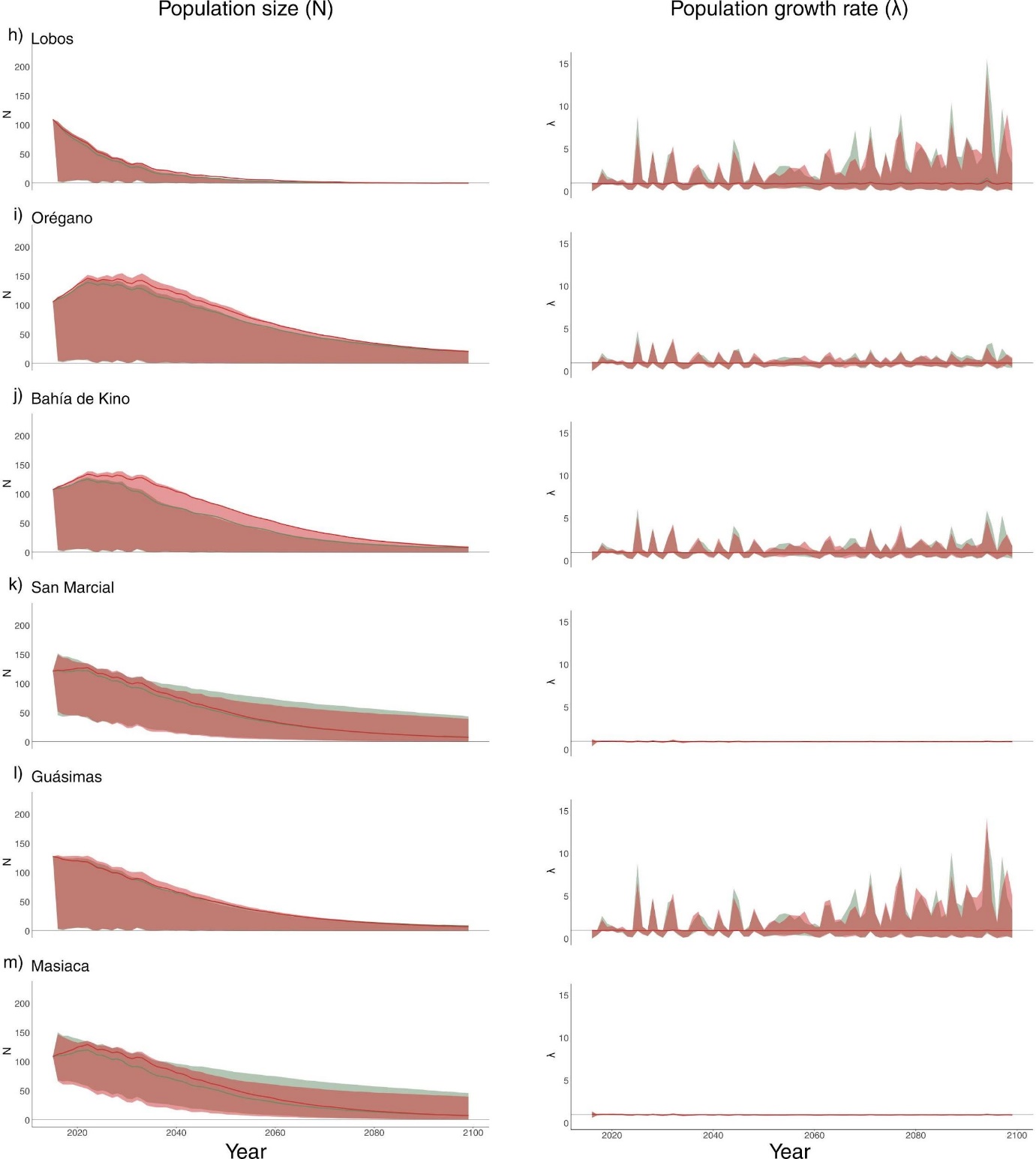

Supplement: mcae094_suppl_Supplementary_Figures_S1-S4 [file mcae094_suppl_supplementary_figures_s1-s4.docx]
