# Supplementary material for: Population decline of the saguaro cactus throughout its distribution is associated with climate change
Source: Ann Bot. 2024 Jun 8;135(1-2):317–28. doi: 10.1093/aob/mcae094 (PMC11805942; doi:10.1093/aob/mcae094)
Supplement: mcae094_suppl_Supplementary_Tables_S1-S6 [file mcae094_suppl_supplementary_tables_s1-s6.docx]

**Title:** Population decline of the saguaro cactus throughout its distribution is associated with climate change

Authors: Ricardo E. Félix-Burruel, Eugenio Larios, Edgar J. González, and Alberto Búrquez

**This supplementary document includes:**

**Supplementary Table S1:** Best predictive models used in the construction of the survival average model.

**Supplementary Table S2:** Best predictive models used in the construction of the growth average model.

**Supplementary Table S3:** Best predictive models used in the construction of the inverse growth average model.

**Supplementary Table S4:** Best predictive models used in the construction of the probability of recruitment average model.

**Supplementary Table S5:** Best predictive models used in the construction of the number of recruits average model.

**Supplementary Table S6:** Statistics of the growth rate time series projected for 13 saguaro populations from 2017 to 2099 under two climate change scenarios.

**Table S1.** Best predictive models used in the construction of the survival average model, where *z* refers to individual size, *w* to soil water content at saturation, and *p_t_* to the Palmer Drought Severity Index at time *t* , *p_t_*_-1_ Palmer Drought Severity Index at time *t*-1, and *S* to the random effect of Site. For each variable, subscript s refers to standardization, subscript l refers to the natural log transformation of individual size, and function s() refers to a spline (i.e., non-linear model).

| **Model** | | **MRMSE** | **Cumulative MRMSE weight** |
| --- | --- | --- | --- |
| **Fixed effect** | **Random effect** |  |  |

| s(*z*_l,s_) + *w*_s_ + *p_t_*_-1,s_ | 1\|*S* | 0.2308 | 0.0147 |
| --- | --- | --- | --- |
| s(*z*_l,s_) + *w*_s_ | 1\|*S* | 0.2308 | 0.0292 |
| s(*z*_l,s_) + *p_t_*_,s_ + *w*_s_ | 1\|*S* | 0.2309 | 0.0436 |
| s(*z*_l,s_) + *w*_s_ | 1 + *w*_s_\|*S* | 0.2309 | 0.0577 |
| s(*z*_l,s_) + *p_t_*_,s_ + *w*_s_ | 0 + *p_t_*_,s_\|*S* | 0.2311 | 0.0713 |
| s(*z*_l,s_) + *w*_s_ + *p_t_*_-1,s_ | 0 + *z*_l,s_\|*S* | 0.2312 | 0.0847 |
| s(*z*_l,s_) + *p_t_*_,s_ + *w*_s_ | 0 + *z*_l,s_\|*S* | 0.2313 | 0.0978 |
| s(*z*_l,s_) + *w*_s_ | 0 + *z*_l,s_\|*S* | 0.2313 | 0.1108 |
| s(*z*_l,s_) + *p_t_*_,s_ + *w*_s_ | 1 + *w*_s_\|*S* | 0.2313 | 0.1238 |
| s(*z*_l,s_) + *p_t_*_,s_ + *w*_s_ | 1 + *p_t_*_,s_\|*S* | 0.2313 | 0.1367 |
| s(*z*_l,s_) + *w*_s_ + *p_t_*_-1,s_ | 0 + *p_t_*_-1,s_\|*S* | 0.2313 | 0.1497 |
| s(*z*_l,s_) + *w*_s_ | 1 + *z*_l,s_\|*S* | 0.2314 | 0.1623 |
| s(*z*_l,s_) + *p_t_*_,s_ + *w*_s_ | 1 + *z*_l,s_\|*S* | 0.2315 | 0.1747 |
| s(*z*_l,s_) + *p_t_*_,s_ + *w*_s_ | 0 + *w*_s_\|*S* | 0.2324 | 0.1849 |
| s(*z*_l,s_) + *w*_s_ | 0 + *w*_s_\|*S* | 0.2325 | 0.1949 |
| s(*z*_l,s_) + *w*_s_ + *p_t_*_-1,s_ | 0 + *w*_s_\|*S* | 0.2325 | 0.2047 |
| s(*z*_l,s_) + *p_t_*_,s_ | 0 + *z*_l,s_\|*S* | 0.2326 | 0.2145 |
| s(*z*_l,s_) | 0 + *z*_l,s_\|*S* | 0.2326 | 0.2243 |
| s(*z*_l,s_) + *p_t_*_-1,s_ | 0 + *z*_l,s_\|*S* | 0.2326 | 0.2339 |
| s(*z*_l,s_) + *p_t_*_,s_ | 0 + *p_t_*_,s_\|*S* | 0.2328 | 0.2433 |
| s(*z*_l,s_) + *p_t_*_,s_ | 1 + *z*_l,s_\|*S* | 0.2328 | 0.2525 |
| s(*z*_l,s_) | 1 + *z*_l,s_\|*S* | 0.2328 | 0.2617 |
| s(*z*_l,s_) + *p_t_*_-1,s_ | 1 + *z*_l,s_\|*S* | 0.2328 | 0.2709 |
| *z*_l,s_ + *p_t_*_,s_ +*w*_s_ + *z*_l,s_ *p_t_*_,s_ + z_l,s_*w*_s_ + *p_t_*_,s_*w*_s_ + *z*_l,s_*p_t_*_,s_*w*_s_ | 1\|*S* | 0.233 | 0.2798 |
| s(*z*_l,s_) + *p_t_*_,s_ | 1 + *p_t_*_,s_\|*S* | 0.233 | 0.2886 |
| *z*_l,s_ + *p_t_*_,s_ +*w*_s_ + *z*_l,s_ *p_t_*_,s_ + z_l,s_*w*_s_ + *p_t_*_,s_*w*_s_ + *z*_l,s_*p_t_*_,s_*w*_s_ | 1 + *w*_s_\|*S* | 0.233 | 0.2973 |
| s(*z*_l,s_) + *p_t_*_,s_ | 1\|*S* | 0.2331 | 0.3059 |
| *z*_l,s_ + *p_t_*_,s_ +*w*_s_ + *z*_l,s_ *p_t_*_,s_ + z_l,s_*w*_s_ + *p_t_*_,s_*w*_s_ + *z*_l,s_*p_t_*_,s_*w*_s_ | 1 + *p_t_*_,s_\|*S* | 0.2331 | 0.3145 |
| s(*z*_l,s_) + *p_t_*_-1,s_ | 1\|*S* | 0.2332 | 0.323 |
| *z*_l,s_ + *w*_s_ + s(*p_t_*_-1,s_) | 0 + *p_t_*_-1,s_\|*S* | 0.2332 | 0.3314 |
| *z*_l,s_ + s(*w*_s_) + *p_t_*_-1,s_ | 0 + *p_t_*_-1,s_\|*S* | 0.2332 | 0.3399 |
| s(*z*_l,s_) | 1\|*S* | 0.2332 | 0.3483 |
| *z*_l,s_ + *p_t_*_,s_ + *w*_s_ + *z*_l,s_ *p_t_*_,s_ | 1 + *w*_s_\|*S* | 0.2332 | 0.3568 |
| *z*_l,s_ + *p_t_*_,s_ + *w*_s_ + *z*_l,s_ *p_t_*_,s_ | 1\|*S* | 0.2332 | 0.3652 |
| *z*_l,s_ + *p_t_*_,s_ + *w*_s_ + *z*_l,s_ *p_t_*_,s_ | 0 + *w*_s_\|*S* | 0.2333 | 0.3735 |
| s(*z*_l,s_) + *p_t_*_-1,s_ | 0 + *p_t_*_-1,s_\|*S* | 0.2333 | 0.3818 |
| *z*_l,s_ + *w*_s_ + *z*_l,s_ *w*_s_ | 1\|*S* | 0.2333 | 0.39 |
| *z*_l,s_ + *w*_s_ + *z*_l,s_ *w*_s_ | 1 + *w*_s_\|*S* | 0.2334 | 0.3982 |
| *z*_l,s_ + s(*w*_s_) + *p_t_*_-1,s_ | 1\|*S* | 0.2334 | 0.4062 |
| *z*_l,s_ + *w*_s_ + s(*p_t_*_-1,s_) | 1\|*S* | 0.2334 | 0.4142 |
| s(*z*_l,s_) + *p_t_*_-1,s_ | 1 + *p_t_*_-1,s_\|*S* | 0.2334 | 0.4222 |
| *z*_l,s_ + *p_t_*_,s_ + *w*_s_ + *z*_l,s_ *p_t_*_,s_ | 1 + *p_t_*_,s_\|*S* | 0.2335 | 0.4302 |
| *z*_l,s_ + *p_t_*_,s_ + *w*_s_ + *z*_l,s_ *p_t_*_,s_ + z_l,s_*w*_s_ + *p_t_*_,s_*w*_s_ + *z*_l,s_*p_t_*_,s_*w*_s_ | 0 + *w*_s_\|*S* | 0.2335 | 0.4381 |
| *z*_l,s_ + s(*w*_s_) | 0 + *w*_s_\|*S* | 0.2335 | 0.4459 |
| *z*_l,s_ + s(*w*_s_) + *p_t_*_-1,s_ | 0 + *w*_s_\|*S* | 0.2336 | 0.4537 |
| *z*_l,s_ + *w*_s_ + s(*p_t_*_-1,s_) | 0 + *w*_s_\|*S* | 0.2336 | 0.4615 |
| s(*z*_l,s_) + s(*w*_s_) | 1 + *w*_s_\|*S* | 0.2336 | 0.4693 |
| s(*z*_l,s_) + *w*_s_ | 1 + *w*_s_\|*S* | 0.2336 | 0.477 |
| *z*_l,s_ + s(*w*_s_) + *p_t_*_-1,s_ | 1 + *w*_s_\|*S* | 0.2336 | 0.4848 |
| *z*_l,s_ + *w*_s_ + s(*p_t_*_-1,s_) | 1 + *w*_s_\|*S* | 0.2336 | 0.4925 |
| *z*_l,s_ + *p_t_*_,s_ + s(*w*_s_) | 0 + *w*_s_\|*S* | 0.2336 | 0.5002 |
| s(*z*_l,s_) + *w*_s_ | 0 + *w*_s_\|*S* | 0.2337 | 0.5079 |
| *z*_l,s_ + s(*p_t_*_,s_) + *w*_s_ | 0 + *w*_s_\|*S* | 0.2337 | 0.5155 |
| *z*_l,s_ + *p_t_*_,s_ + *w*_s_ | 0 + *w*_s_\|*S* | 0.2337 | 0.523 |
| *z*_l,s_ + *p_t_*_,s_ + *p_t_*_,s_ *w*_s_ | 1 + *w*_s_\|*S* | 0.2337 | 0.5306 |
| *z*_l,s_ + *p_t_*_,s_ + *w*_s_ | 1 + *w*_s_\|*S* | 0.2337 | 0.5381 |
| *z*_l,s_ + s(*p_t_*_,s_) + *w*_s_ | 1 + *w*_s_\|*S* | 0.2337 | 0.5456 |
| *z*_l,s_ + *w*_s_ + *z*_l,s_ *w*_s_ | 0 + *w*_s_\|*S* | 0.2337 | 0.5531 |
| *z*_l,s_ + *p_t_*_,s_ + s(*w*_s_) | 1 + *w*_s_\|*S* | 0.2337 | 0.5606 |
| s(*z*_l,s_) + *w*_s_ | 1\|*S* | 0.2337 | 0.568 |
| *z*_l,s_ + s(*w*_s_) | 1\|*S* | 0.2337 | 0.5755 |
| *z*_l,s_ + *p_t_*_,s_ + *w*_s_ | 1\|*S* | 0.2338 | 0.583 |
| *z*_l,s_ + *p_t_*_,s_ + s(*w*_s_) | 1\|*S* | 0.2338 | 0.5904 |
| *z*_l,s_ + s(*p_t_*_,s_) + *w*_s_ | 1\|*S* | 0.2338 | 0.5979 |
| *z*_l,s_ + *p_t_*_,s_ + *p_t_*_,s_ *w*_s_ | 1 + *p_t_*_,s_\|*S* | 0.2338 | 0.6054 |
| *z*_l,s_ + s(*p_t_*_,s_) + *w*_s_ | 1 + *p_t_*_,s_\|*S* | 0.2338 | 0.6128 |
| *z*_l,s_ + *p_t_*_,s_ + s(*w*_s_) | 1 + *p_t_*_,s_\|*S* | 0.2338 | 0.6203 |
| *z*_l,s_ + *p_t_*_,s_ + *w*_s_ | 1 + *p_t_*_,s_\|*S* | 0.2338 | 0.6278 |
| *z*_l,s_ + *p_t_*_,s_ + *p_t_*_,s_ *w*_s_ | 1\|*S* | 0.2338 | 0.6352 |
| *z*_l,s_ + *p_t_*_,s_ + *z*_l,s_ *p_t_*_,s_ | 1\|*S* | 0.2338 | 0.6426 |
| *z*_l,s_ + s(*w*_s_) + *p_t_*_-1,s_ | 1 + *p_t_*_-1,s_\|*S* | 0.2338 | 0.65 |
| *z*_l,s_ + *w*_s_ + s(*p_t_*_-1,s_) | 1 + *p_t_*_-1,s_\|*S* | 0.2338 | 0.6573 |
| *z*_l,s_ + *p_t_*_,s_ + s(*w*_s_) | 0 + *p_t_*_,s_\|*S* | 0.2338 | 0.6646 |
| *z*_l,s_ + s(*p_t_*_,s_) + *w*_s_ | 0 + *p_t_*_,s_\|*S* | 0.2338 | 0.6719 |
| *z*_l,s_ + *p_t_*_,s_ + *w*_s_ | 0 + *p_t_*_,s_\|*S* | 0.2338 | 0.6792 |
| *z*_l,s_ + *p_t_*_,s_ + *p_t_*_,s_ *w*_s_ | 0 + *p_t_*_,s_\|*S* | 0.2339 | 0.6865 |
| *z*_l,s_ + *p_t_*_,s_ + *p_t_*_,s_ *w*_s_ | 0 + *w*_s_\|*S* | 0.2339 | 0.6937 |
| *z*_l,s_ + *p_t_*_-1,s_ | 1\|*S* | 0.234 | 0.7008 |
| *z*_l,s_ + *p_t_*_-1,s_ | 1\|*S* | 0.234 | 0.708 |
| *z*_l,s_ | 1\|*S* | 0.234 | 0.715 |
| *z*_l,s_ + s(*p_t_*_,s_) | 1\|*S* | 0.234 | 0.722 |
| *z*_l,s_ + *p_t_*_,s_ | 1\|*S* | 0.234 | 0.729 |
| *z*_l,s_ + *p_t_*_,s_ | 1 + *p_t_*_,s_\|*S* | 0.234 | 0.736 |
| *z*_l,s_ + s(*p_t_*_-1,s_) | 1 + *p_t_*_,s_\|*S* | 0.234 | 0.743 |
| *z*_l,s_ + *p_t_*_,s_ + *z*_l,s_ *p*_t,s_ | 1 + *p_t_*_,s_\|*S* | 0.2341 | 0.75 |
| *z*_l,s_ + s(*p_t_*_-1,s_) | 1 + *p_t_*_-1,s_\|*S* | 0.2342 | 0.7567 |
| *z*_l,s_ + *p_t_*_-1,s_ | 1 + *p_t_*_-1,s_\|*S* | 0.2342 | 0.7634 |
| *z*_l,s_ + s(*p_t_*_-1,s_) | 0 + *p_t_*_-1,s_\|*S* | 0.2342 | 0.7701 |
| *z*_l,s_ + *p_t_*_-1,s_ | 0 + *p_t_*_-1,s_\|*S* | 0.2342 | 0.7768 |
| *z*_l,s_ + *p_t_*_-1,s_ + *z*_l,s_ *p_t_*_-1,s_ | 1\|*S* | 0.2343 | 0.7833 |
| *z*_l,s_ + *p_t_*_,s_ | 0 + *p_t_*_,s_\|*S* | 0.2345 | 0.7896 |
| *z*_l,s_ + s(*p_t_*_,s_) | 0 + v\|*S* | 0.2345 | 0.7959 |
| *z*_l,s_ + *p_t_*_-1,s_ + *z*_l,s_ *p_t_*_-1,s_ | 1 + *p_t_*_-1,s_\|*S* | 0.2347 | 0.8019 |
| *z*_l,s_ + *p_t_*_,s_ + *w*_s_ + *z*_l,s_ *p_t_*_,s_ + z_l,s_*w*_s_ + *p_t_*_,s_*w*_s_ + *z*_l,s_*p_t_*_,s_*w*_s_ | 0 + *p_t_*_,s_\|*S* | 0.2348 | 0.8078 |
| *z*_l,s_ + *p_t_*_-1,s_ + *z*_l,s_ *p_t_*_-1,s_ | 0 + *p_t_*_-1,s_\|*S* | 0.2348 | 0.8137 |
| *z*_l,s_ + *p_t_*_-1,s_ + *z*_l,s_ *p_t_*_-1,s_ | 1 + *z*_l,s_\|*S* | 0.2349 | 0.8194 |
| *z*_l,s_ + *w*_s_ + s(*p_t_*_-1,s_) | 1 + *z*_l,s_\|*S* | 0.235 | 0.825 |
| *z*_l,s_ + s(*w*_s_) + *p_t_*_-1,s_ | 1 + *z*_l,s_\|*S* | 0.235 | 0.8305 |
| *z*_l,s_ + *p_t_*_-1,s_ + *z*_l,s_ *p_t_*_-1,s_ | 0 + *z*_l,s_\|*S* | 0.2352 | 0.8359 |
| *z*_l,s_ + *p_t_*_,s_ + *w*_s_ + *z*_l,s_ *p_t_*_,s_ | 0 + *p_t_*_,s_\|*S* | 0.2352 | 0.8413 |
| *z*_l,s_ + s(*w*_s_) + *p_t_*_-1,s_ | 0 + *z*_l,s_\|*S* | 0.2353 | 0.8466 |
| *z*_l,s_ + *w*_s_ + s(*p_t_*_-1,s_) | 0 + *z*_l,s_\|*S* | 0.2353 | 0.8519 |
| *z*_l,s_ + s(*p_t_*_,s_) | 1 + *z*_l,s_\|*S* | 0.2353 | 0.8572 |
| *z*_l,s_ + *p_t_*_-1,s_ | 1 + *z*_l,s_\|*S* | 0.2353 | 0.8624 |
| *z*_l,s_ + s(*w*_s_) | 1 + *z*_l,s_\|*S* | 0.2353 | 0.8676 |
| s(*z*_l,s_) + *w*_s_ | 1 + *z*_l,s_\|*S* | 0.2353 | 0.8728 |
| *z*_l,s_ + s(*p_t_*_,s_) + *w*_s_ | 1 + *z*_l,s_\|*S* | 0.2354 | 0.878 |
| *z*_l,s_ + *p_t_*_,s_ + *w*_s_ | 1 + *z*_l,s_\|*S* | 0.2354 | 0.8832 |
| *z*_l,s_ + *p_t_*_,s_ + s(*w*_s_) | 1 + *z*_l,s_\|*S* | 0.2354 | 0.8883 |
| *z*_l,s_ + *p_t_*_,s_ + *p_t_*_,s_ *w*_s_ | 1 + *z*_l,s_\|*S* | 0.2354 | 0.8935 |
| *z*_l,s_ | 1 + *z*_l,s_\|*S* | 0.2354 | 0.8986 |
| *z*_l,s_ + s(*p_t_*_,s_) | 1 + *z*_l,s_\|*S* | 0.2355 | 0.9037 |
| *z*_l,s_ + *p_t_*_,s_ | 1 + *z*_l,s_\|*S* | 0.2355 | 0.9088 |
| s(*z*_l,s_) + *w*_s_ | 0 + *z*_l,s_\|*S* | 0.2356 | 0.9138 |
| *z*_l,s_ + s(*w*_s_) | 0 + *z*_l,s_\|*S* | 0.2356 | 0.9187 |
| *z*_l,s_ + *p_t_*_,s_ + s(*w*_s_) | 0 + *z*_l,s_\|*S* | 0.2356 | 0.9237 |
| *z*_l,s_ + s(*p_t_*_,s_) + *w*_s_ | 0 + *z*_l,s_\|*S* | 0.2356 | 0.9286 |
| *z*_l,s_ + *p_t_*_,s_ + *w*_s_ | 0 + *z*_l,s_\|*S* | 0.2356 | 0.9336 |
| *z*_l,s_ + *p_t_*_,s_ + *w*_s_ + *z*_l,s_ *p_t_*_,s_ | 0 + *z*_l,s_\|*S* | 0.2356 | 0.9385 |
| *z*_l,s_ + *p_t_*_,s_ + *p_t_*_,s_ *w*_s_ | 0 + *z*_l,s_\|*S* | 0.2357 | 0.9433 |
| *z*_l,s_ + *p_t_*_,s_ + *w*_s_ + *z*_l,s_ *p_t_*_,s_ | 1 + *z*_l,s_\|*S* | 0.2358 | 0.948 |

**Table S2.** Best predictive models used in the construction of the growth average model, where *z* refers to individual size, *w* to soil water content at saturation, and *p_t_* to the Palmer Drought Severity Index at time *t* , *p_t_*_-1_ Palmer Drought Severity Index at time *t*-1, and *S* to the random effect of Site. For each variable, subscript s refers to standardization, subscript l refers to the natural log transformation of individual size, and function s() refers to a spline (i.e., non-linear model).

| **Model** | | **MRMSE** | **Cumulative MRMSE weight** |
| --- | --- | --- | --- |
| **Fixed effect** | **Random effect** |  |  |
| s(*z*_l,s_) + s(*w*_s_) + s(*p_t_*_-1,s_) | 0 + *w*_s_\|*S* | 0.0687 | 0.0062 |
| s(*z*_l,s_) + *w*_s_ + s(*p_t_*_-1,s_) | 0 + *w*_s_\|*S* | 0.0687 | 0.0124 |
| s(*z*_l,s_) + *w*_s_ + s(*p_t_*_-1,s_) | 1 + *w*_s_\|*S* | 0.0688 | 0.0184 |
| s(*z*_l,s_) + s(*w*_s_) + s(*p_t_*_-1,s_) | 1 + *w*_s_\|*S* | 0.0688 | 0.0245 |
| s(*z*_l,s_) + s(*w*_s_) + s(*p_t_*_-1,s_) | 1\|*S* | 0.0688 | 0.0305 |
| s(*z*_l,s_) + *w*_s_ + s(*p_t_*_-1,s_) | 1\|*S* | 0.0688 | 0.0366 |
| s(*z*_l,s_) + s(*w*_s_) + s(*p_t_*_-1,s_) | 0 + *z*_l,s_\|*S* | 0.0689 | 0.0426 |
| s(*z*_l,s_) + *w*_s_ + s(*p_t_*_-1,s_) | 0 + *z*_l,s_\|*S* | 0.0689 | 0.0485 |
| s(*z*_l,s_) + s(*p_t_*_-1,s_) | 0 + *z*_l,s_\|*S* | 0.0689 | 0.0545 |
| s(*z*_l,s_) + s(*p_t_*_-1,s_) | 1\|*S* | 0.0690 | 0.0603 |
| s(*z*_l,s_) + *w*_s_ + s(*p_t_*_-1,s_) | 1 + *z*_l,s_\|*S* | 0.0690 | 0.0661 |
| s(*z*_l,s_) + s(*w*_s_) + s(*p_t_*_-1,s_) | 1 + *z*_l,s_\|*S* | 0.0690 | 0.0718 |
| s(*z*_l,s_) + s(*p_t_*_,s_) + *w*_s_ | 1 + *w*_s_\|*S* | 0.0690 | 0.0776 |
| s(*z*_l,s_) + s(*p_t_*_,s_) + s(*w*_s_) | 1 + *w*_s_\|*S* | 0.0690 | 0.0834 |
| s(*z*_l,s_) + *p_t_*_,s_ | 0 + *p_t_*_,s_\|*S* | 0.0690 | 0.0891 |
| s(*z*_l,s_) + s(*p_t_*_,s_) + s(*w*_s_) | 1\|*S* | 0.0691 | 0.0949 |
| s(*z*_l,s_) + s(*p_t_*_,s_) + *w*_s_ | 1\|*S* | 0.0691 | 0.1006 |
| s(*z*_l,s_) + s(*p_t_*_,s_) + s(*w*_s_) | 0 + *w*_s_\|*S* | 0.0691 | 0.1063 |
| s(*z*_l,s_) + s(*p_t_*_,s_) + *w*_s_ | 0 + *w*_s_\|*S* | 0.0691 | 0.1121 |
| s(*z*_l,s_) + s(*p_t_*_,s_) | 1\|*S* | 0.0691 | 0.1178 |
| s(*z*_l,s_) + *p_t_*_,s_ + *w*_s_ + *p_t_*_,s_ *w*_s_ | 1 + *w*_s_\|*S* | 0.0691 | 0.1236 |
| s(*z*_l,s_) + *p_t_*_,s_ + *w*_s_ | 1 + *w*_s_\|*S* | 0.0691 | 0.1293 |
| s(*z*_l,s_) + *p_t_*_,s_ + s(*w*_s_) | 0 + *w*_s_\|*S* | 0.0691 | 0.1350 |
| s(*z*_l,s_) + *p_t_*_,s_ + *w*_s_ | 0 + *w*_s_\|*S* | 0.0691 | 0.1408 |
| s(*z*_l,s_) + *p_t_*_,s_ + s(*w*_s_) | 1 + *w*_s_\|*S* | 0.0691 | 0.1465 |
| s(*z*_l,s_) + *p_t_*_,s_ + s(*w*_s_) | 1\|*S* | 0.0691 | 0.1522 |
| s(*z*_l,s_) + *p_t_*_,s_ + *w*_s_ | 1\|*S* | 0.0691 | 0.1579 |
| s(*z*_l,s_) + *p_t_*_,s_ + *w*_s_ + *p_t_*_,s_ *w*_s_ | 1\|*S* | 0.0691 | 0.1637 |
| s(*z*_l,s_) + *p_t_*_,s_ | 1\|*S* | 0.0691 | 0.1694 |
| s(*z*_l,s_) + *p_t_*_,s_ + *w*_s_ + *p_t_*_,s_ *w*_s_ | 0 + *w*_s_\|*S* | 0.0691 | 0.1751 |
| s(*z*_l,s_) + s(*p_t_*_,s_) | 1 + *z*_l,s_\|*S* | 0.0691 | 0.1808 |
| s(*z*_l,s_) + s(*p_t_*_,s_) | 0 + *z*_l,s_\|*S* | 0.0691 | 0.1865 |
| s(*z*_l,s_) + *p_t_*_,s_ | 0 + *z*_l,s_\|*S* | 0.0691 | 0.1922 |
| s(*z*_l,s_) + s(*p_t_*_,s_) + s(*w*_s_) | 0 + *z*_l,s_\|*S* | 0.0691 | 0.1980 |
| s(*z*_l,s_) + s(*p_t_*_,s_) + *w*_s_ | 0 + *z*_l,s_\|*S* | 0.0691 | 0.2037 |
| s(*z*_l,s_) + *p_t_*_,s_ + *w*_s_ + *p_t_*_,s_ *w*_s_ | 0 + *z*_l,s_\|*S* | 0.0691 | 0.2094 |
| s(*z*_l,s_) + *p_t_*_,s_ + *w*_s_ | 0 + *z*_l,s_\|*S* | 0.0691 | 0.2151 |
| s(*z*_l,s_) + *p_t_*_,s_ + s(*w*_s_) | 0 + *z*_l,s_\|*S* | 0.0691 | 0.2208 |
| s(*z*_l,s_) + *p_t_*_,s_ | 1 + *z*_l,s_\|*S* | 0.0691 | 0.2265 |
| s(*z*_l,s_) + *p_t_*_,s_ + s(*w*_s_) | 1 + *z*_l,s_\|*S* | 0.0691 | 0.2322 |
| s(*z*_l,s_) + *p_t_*_,s_ + *w*_s_ | 1 + *z*_l,s_\|*S* | 0.0691 | 0.2379 |
| s(*z*_l,s_) + *p_t_*_,s_ + *w*_s_ + *p_t_*_,s_ *w*_s_ | 1 + *z*_l,s_\|*S* | 0.0691 | 0.2436 |
| s(*z*_l,s_) + s(*p_t_*_,s_) + *w*_s_ | 1 + *z*_l,s_\|*S* | 0.0691 | 0.2492 |
| s(*z*_l,s_) + s(*p_t_*_,s_) + s(*w*_s_) | 1 + *z*_l,s_\|*S* | 0.0691 | 0.2549 |
| s(*z*_l,s_) + *w*_s_ + *p_t_*_-1,s_ | 0 + *w*_s_\|*S* | 0.0691 | 0.2606 |
| s(*z*_l,s_) + s(*w*_s_) + *p_t_*_-1,s_ | 0 + *w*_s_\|*S* | 0.0691 | 0.2662 |
| s(*z*_l,s_) + *w*_s_ + *p_t_*_-1,s_ + *w*_s_ *p_t_*_-1,s_ | 0 + *w*_s_\|*S* | 0.0691 | 0.2719 |
| s(*z*_l,s_) + s(*p_t_*_-1,s_) | 1 + *z*_l,s_\|*S* | 0.0692 | 0.2775 |
| s(*z*_l,s_) + *w*_s_ | 0 + *w*_s_\|*S* | 0.0693 | 0.2830 |
| s(*z*_l,s_) + s(*w*_s_) | 0 + *w*_s_\|*S* | 0.0693 | 0.2885 |
| s(*z*_l,s_) + *w*_s_ | 1 + *w*_s_\|*S* | 0.0693 | 0.2939 |
| s(*z*_l,s_) + s(*w*_s_) | 1 + *w*_s_\|*S* | 0.0693 | 0.2994 |
| s(*z*_l,s_) + s(*w*_s_) | 1\|*S* | 0.0693 | 0.3049 |
| s(*z*_l,s_) + *w*_s_ | 1\|*S* | 0.0693 | 0.3104 |
| s(*z*_l,s_) | 1\|*S* | 0.0693 | 0.3158 |
| s(*z*_l,s_) + *w*_s_ | 0 + *z*_l,s_\|*S* | 0.0693 | 0.3213 |
| s(*z*_l,s_) + s(*w*_s_) | 0 + *z*_l,s_\|*S* | 0.0693 | 0.3267 |
| s(*z*_l,s_) | 0 + *z*_l,s_\|*S* | 0.0693 | 0.3322 |
| s(*z*_l,s_) + *w*_s_ | 1 + *z*_l,s_\|*S* | 0.0693 | 0.3376 |
| s(*z*_l,s_) + s(*w*_s_) | 1 + *z*_l,s_\|*S* | 0.0693 | 0.3430 |
| s(*z*_l,s_) | 1 + *z*_l,s_\|*S* | 0.0693 | 0.3484 |
| s(*z*_l,s_) + *p_t_*_,s_ + s(*w*_s_) | 0 + *p_t_*_,s_\|*S* | 0.0694 | 0.3538 |
| s(*z*_l,s_) + *p_t_*_,s_ + *w*_s_ | 0 + *p_t_*_,s_\|*S* | 0.0694 | 0.3592 |
| s(*z*_l,s_) + *p_t_*_,s_ + *w*_s_ + *p_t_*_,s_ *w*_s_ | 0 + *p_t_*_,s_\|*S* | 0.0694 | 0.3645 |
| s(*z*_l,s_) + *p_t_*_-1,s_ | 0 + *z*_l,s_\|*S* | 0.0695 | 0.3698 |
| s(*z*_l,s_) + *w*_s_ + *p_t_*_-1,s_ + *w*_s_ *p_t_*_-1,s_ | 0 + *z*_l,s_\|*S* | 0.0695 | 0.3750 |
| s(*z*_l,s_) + s(*w*_s_) + *p_t_*_-1,s_ | 0 + *z*_l,s_\|*S* | 0.0695 | 0.3802 |
| s(*z*_l,s_) + *w*_s_ + *p_t_*_-1,s_ | 0 + *z*_l,s_\|*S* | 0.0695 | 0.3853 |
| *z*_l,s_ + *w*_s_ + *p_t_*_-1,s_ + *z*_l,s_ *w*_s_ + *z*_l,s_ *p_t_*_-1,s_ + *w*_s_ *p_t_*_-1,s_ | 0 + *w*_s_\|*S* | 0.0696 | 0.3904 |
| s(*z*_l,s_) + *w*_s_ + *p_t_*_-1,s_ | 1 + *w*_s_\|*S* | 0.0697 | 0.3955 |
| s(*z*_l,s_) + s(*w*_s_) + *p_t_*_-1,s_ | 1 + *w*_s_\|*S* | 0.0697 | 0.4005 |
| s(*z*_l,s_) + *w*_s_ + *p_t_*_-1,s_ + *w*_s_ *p_t_*_-1,s_ | 1 + *w*_s_\|*S* | 0.0697 | 0.4055 |
| s(*z*_l,s_) + *w*_s_ + *p_t_*_-1,s_ + *w*_s_ *p_t_*_-1,s_ | 1\|*S* | 0.0697 | 0.4105 |
| *z*_l,s_ + *w*_s_ + *p_t_*_-1,s_ + *z*_l,s_ *w*_s_ + *z*_l,s_ *p_t_*_-1,s_ + *w*_s_ *p_t_*_-1,s_ | 1 + *w*_s_\|*S* | 0.0697 | 0.4155 |
| s(*z*_l,s_) + *w*_s_ + *p_t_*_-1,s_ | 1\|*S* | 0.0697 | 0.4205 |
| s(*z*_l,s_) + s(*w*_s_) + *p_t_*_-1,s_ | 1\|*S* | 0.0697 | 0.4254 |
| *z*_l,s_ + *w*_s_ + *p_t_*_-1,s_ + *z*_l,s_ *w*_s_ + *z*_l,s_ *p_t_*_-1,s_ + *w*_s_ *p_t_*_-1,s_ | 1 + *z*_l,s_\|*S* | 0.0698 | 0.4304 |
| *z*_l,s_ + *w*_s_ + *p_t_*_-1,s_ + *z*_l,s_ *w*_s_ + *z*_l,s_ *p_t_*_-1,s_ + *w*_s_ *p_t_*_-1,s_ | 1\|*S* | 0.0698 | 0.4353 |
| *z*_l,s_ + *w*_s_ + *p_t_*_-1,s_ + *z*_l,s_ *w*_s_ + *z*_l,s_ *p_t_*_-1,s_ + *w*_s_ *p_t_*_-1,s_ | 0 + *z*_l,s_\|*S* | 0.0698 | 0.4403 |
| s(*z*_l,s_) + *p_t_*_-1,s_ | 1\|*S* | 0.0698 | 0.4451 |
| s(*z*_l,s_) + *w*_s_ + *p_t_*_-1,s_ + *w*_s_ *p_t_*_-1,s_ | 1 + *z*_l,s_\|*S* | 0.0699 | 0.4500 |
| s(*z*_l,s_) + *w*_s_ + *p_t_*_-1,s_ | 1 + *z*_l,s_\|*S* | 0.0699 | 0.4548 |
| s(*z*_l,s_) + s(*w*_s_) + *p_t_*_-1,s_ | 1 + *z*_l,s_\|*S* | 0.0699 | 0.4596 |
| s(*z*_l,s_) + *p_t_*_-1,s_ | 1 + *z*_l,s_\|*S* | 0.0700 | 0.4643 |
| s(*z*_l,s_) + *w*_s_ + s(*p_t_*_-1,s_) | 0 + *p_t_*_-1,s_\|*S* | 0.0701 | 0.4689 |
| s(*z*_l,s_) + s(*w*_s_) + s(*p_t_*_-1,s_) | 0 + *p_t_*_-1,s_\|*S* | 0.0701 | 0.4735 |
| *z*_l,s_ + *p_t_*_-1,s_ + *z*_l,s_ *p_t_*_-1,s_ | 0 + *z*_l,s_\|*S* | 0.0702 | 0.4780 |
| *z*_l,s_ + *p_t_*_-1,s_ + *z*_l,s_ *p_t_*_-1,s_ | 1 + *z*_l,s_\|*S* | 0.0702 | 0.4825 |
| s(*z*_l,s_) + *p_t_*_-1,s_ | 0 + *p_t_*_-1,s_\|*S* | 0.0702 | 0.4870 |
| s(*z*_l,s_) + s(*p_t_*_-1,s_) | 0 + *p_t_*_-1,s_\|*S* | 0.0702 | 0.4915 |
| *z*_l,s_ + *p_t_*_-1,s_ + *z*_l,s_ *p_t_*_-1,s_ | 1\|*S* | 0.0704 | 0.4958 |
| s(*z*_l,s_) + s(*w*_s_) + *p_t_*_-1,s_ | 0 + *p_t_*_-1,s_\|*S* | 0.0704 | 0.5001 |
| s(*z*_l,s_) + *w*_s_ + *p_t_*_-1,s_ | 0 + *p_t_*_-1,s_\|*S* | 0.0704 | 0.5044 |
| *z*_l,s_ + *w*_s_ + *p_t_*_-1,s_ + *z*_l,s_ *w*_s_ + *z*_l,s_ *p_t_*_-1,s_ + *w*_s_ *p_t_*_-1,s_ | 0 + *p_t_*_-1,s_\|*S* | 0.0705 | 0.5086 |
| s(*z*_l,s_) + *w*_s_ + *p_t_*_-1,s_ + *w*_s_ *p_t_*_-1,s_ | 0 + *p_t_*_-1,s_\|*S* | 0.0705 | 0.5129 |
| s(*z*_l,s_) + *w*_s_ + *p_t_*_-1,s_ + *w*_s_ *p_t_*_-1,s_ | 1 + *p_t_*_-1,s_\|*S* | 0.0705 | 0.5172 |
| *z*_l,s_ + *w*_s_ + s(*p_t_*_-1,s_) + *z*_l,s_ *w*_s_ | 0 + *w*_s_\|*S* | 0.0706 | 0.5213 |
| *z*_l,s_ + *p_t_*_-1,s_ + *z*_l,s_ *p_t_*_-1,s_ | 0 + *p_t_*_-1,s_\|*S* | 0.0707 | 0.5253 |
| *z*_l,s_ + *p_t_*_,s_ + *w*_s_ + *z*_l,s_ *p_t_*_,s_ + *z*_l,s_ *w*_s_ + *p_t_*_,s_ *w*_s_ | 1 + *w*_s_\|*S* | 0.0708 | 0.5293 |
| *z*_l,s_ + *p_t_*_,s_ + *w*_s_ + *z*_l,s_ *p_t_*_,s_ + *z*_l,s_ *w*_s_ + *p_t_*_,s_ *w*_s_ | 1\|*S* | 0.0708 | 0.5333 |
| *z*_l,s_ + *p_t_*_,s_ + *w*_s_ + *z*_l,s_ *p_t_*_,s_ + *z*_l,s_ *w*_s_ + *p_t_*_,s_ *w*_s_ | 0 + *w*_s_\|*S* | 0.0708 | 0.5373 |
| *z*_l,s_ + *w*_s_ + s(*p_t_*_-1,s_) + *z*_l,s_ *w*_s_ | 1\|*S* | 0.0709 | 0.5412 |
| *z*_l,s_ + *w*_s_ + s(*p_t_*_-1,s_) + *z*_l,s_ *w*_s_ | 1 + *w*_s_\|*S* | 0.0709 | 0.5452 |
| *z*_l,s_ + *p_t_*_,s_ + *w*_s_ + *z*_l,s_ *p_t_*_,s_ + *z*_l,s_ *w*_s_ + *p_t_*_,s_ *w*_s_ | 1 + *z*_l,s_\|*S* | 0.0711 | 0.5489 |
| *z*_l,s_ + *w*_s_ + s(*p_t_*_-1,s_) | 0 + *w*_s_\|*S* | 0.0711 | 0.5526 |
| *z*_l,s_ + s(*w*_s_) + s(*p_t_*_-1,s_) | 0 + *w*_s_\|*S* | 0.0711 | 0.5564 |
| *z*_l,s_ + *p_t_*_,s_ + *w*_s_ + *z*_l,s_ *p_t_*_,s_ + *z*_l,s_ *w*_s_ + *p_t_*_,s_ *w*_s_ | 0 + *z*_l,s_\|*S* | 0.0711 | 0.5601 |
| s(*z*_l,s_) + s(*p_t_*_-1,s_) | 0 + *p_t_*_,s_\|*S* | 0.0712 | 0.5637 |
| *z*_l,s_ + *w*_s_ + *p_t_*_-1,s_ + *z*_l,s_ *w*_s_ | 0 + *w*_s_\|*S* | 0.0712 | 0.5674 |
| *z*_l,s_ + *w*_s_ + *z*_l,s_ *w*_s_ | 0 + *w*_s_\|*S* | 0.0713 | 0.5710 |
| *z*_l,s_ + *p_t_*_,s_ + *w*_s_ + *z*_l,s_ *p_t_*_,s_ + *z*_l,s_ *w*_s_ + *p_t_*_,s_ *w*_s_ | 0 + *p_t_*_,s_\|*S* | 0.0713 | 0.5746 |
| *z*_l,s_ + *w*_s_ + *p_t_*_-1,s_ + *z*_l,s_ *w*_s_ + *z*_l,s_ *p_t_*_-1,s_ + *w*_s_ *p_t_*_-1,s_ | 1 + *p_t_*_-1,s_\|*S* | 0.0713 | 0.5782 |
| *z*_l,s_ + *w*_s_ + *z*_l,s_ *w*_s_ | 1 + *w*_s_\|*S* | 0.0713 | 0.5817 |
| *z*_l,s_ + *w*_s_ + s(*p_t_*_-1,s_) | 1 + *w*_s_\|*S* | 0.0713 | 0.5853 |
| *z*_l,s_ + s(*w*_s_) + s(*p_t_*_-1,s_) | 1 + *w*_s_\|*S* | 0.0713 | 0.5889 |
| s(*z*_l,s_) + *w*_s_ + *p_t_*_-1,s_ | 1 + *p_t_*_-1,s_\|*S* | 0.0713 | 0.5924 |
| s(*z*_l,s_) + *w*_s_ + s(*p_t_*_-1,s_) | 1 + *p_t_*_-1,s_\|*S* | 0.0713 | 0.5960 |
| s(*z*_l,s_) + s(*w*_s_) + s(*p_t_*_-1,s_) | 1 + *p_t_*_-1,s_\|*S* | 0.0713 | 0.5995 |
| s(*z*_l,s_) + s(*w*_s_) + *p_t_*_-1,s_ | 1 + *p_t_*_-1,s_\|*S* | 0.0713 | 0.6031 |
| *z*_l,s_ + *w*_s_ + *z*_l,s_ *w*_s_ | 1\|*S* | 0.0713 | 0.6066 |
| *z*_l,s_ + s(*w*_s_) + s(*p_t_*_-1,s_) | 1\|*S* | 0.0713 | 0.6102 |
| *z*_l,s_ + *w*_s_ + s(*p_t_*_-1,s_) | 1\|*S* | 0.0713 | 0.6137 |
| *z*_l,s_ + s(*p_t_*_-1,s_) | 0 + *z*_l,s_\|*S* | 0.0713 | 0.6173 |
| *z*_l,s_ + s(*w*_s_) + s(*p_t_*_-1,s_) | 0 + *z*_l,s_\|*S* | 0.0713 | 0.6208 |
| *z*_l,s_ + s(*w*_s_) + s(*p_t_*_-1,s_) | 1 + *z*_l,s_\|*S* | 0.0714 | 0.6243 |
| *z*_l,s_ + *w*_s_ + s(*p_t_*_-1,s_) | 0 + *z*_l,s_\|*S* | 0.0714 | 0.6278 |
| *z*_l,s_ + *w*_s_ + s(*p_t_*_-1,s_) | 1 + *z*_l,s_\|*S* | 0.0714 | 0.6314 |
| *z*_l,s_ + s(*p_t_*_-1,s_) | 1\|*S* | 0.0714 | 0.6349 |
| *z*_l,s_ + s(*p_t_*_-1,s_) | 1 + *z*_l,s_\|*S* | 0.0714 | 0.6384 |
| *z*_l,s_ + *w*_s_ + s(*p_t_*_-1,s_) + *z*_l,s_ *w*_s_ | 1 + *z*_l,s_\|*S* | 0.0714 | 0.6418 |
| *z*_l,s_ + *p_t_*_,s_ + *w*_s_ + *z*_l,s_ *p_t_*_,s_ | 0 + *w*_s_\|*S* | 0.0715 | 0.6453 |
| *z*_l,s_ + *p_t_*_,s_ + s(*w*_s_) + *z*_l,s_ *p_t_*_,s_ | 0 + *w*_s_\|*S* | 0.0715 | 0.6487 |
| *z*_l,s_ + *p_t_*_,s_ + *w*_s_ + *z*_l,s_ *p_t_*_,s_ | 1 + *w*_s_\|*S* | 0.0715 | 0.6522 |
| *z*_l,s_ + *p_t_*_,s_ + s(*w*_s_) + *z*_l,s_ *p_t_*_,s_ | 1 + *w*_s_\|*S* | 0.0715 | 0.6557 |
| *z*_l,s_ + *p_t_*_,s_ + *z*_l,s_ *p_t_*_,s_ | 1\|*S* | 0.0715 | 0.6591 |
| *z*_l,s_ + *p_t_*_,s_ + *w*_s_ + *z*_l,s_ *p_t_*_,s_ | 1\|*S* | 0.0715 | 0.6626 |
| *z*_l,s_ + *p_t_*_,s_ + s(*w*_s_) + *z*_l,s_ *p_t_*_,s_ | 1\|*S* | 0.0715 | 0.6660 |
| *z*_l,s_ + *p_t_*_,s_ + *z*_l,s_ *p_t_*_,s_ | 1 + *z*_l,s_\|*S* | 0.0715 | 0.6694 |
| *z*_l,s_ + *p_t_*_,s_ + *z*_l,s_ *p_t_*_,s_ | 0 + *z*_l,s_\|*S* | 0.0715 | 0.6728 |
| *z*_l,s_ + *p_t_*_,s_ + s(*w*_s_) + *z*_l,s_ *p_t_*_,s_ | 0 + *z*_l,s_\|*S* | 0.0715 | 0.6763 |
| *z*_l,s_ + *p_t_*_,s_ + *w*_s_ + *z*_l,s_ *p_t_*_,s_ | 0 + *z*_l,s_\|*S* | 0.0715 | 0.6797 |
| *z*_l,s_ + *p_t_*_,s_ + s(*w*_s_) + *z*_l,s_ *p_t_*_,s_ | 1 + *z*_l,s_\|*S* | 0.0715 | 0.6831 |
| *z*_l,s_ + *p_t_*_,s_ + *w*_s_ + *z*_l,s_ *p_t_*_,s_ | 1 + *z*_l,s_\|*S* | 0.0715 | 0.6864 |
| *z*_l,s_ + *w*_s_ + s(*p_t_*_-1,s_) + *z*_l,s_ *w*_s_ | 0 + *z*_l,s_\|*S* | 0.0715 | 0.6898 |
| *z*_l,s_ + s(*w*_s_) + *p_t_*_-1,s_ | 0 + *w*_s_\|*S* | 0.0716 | 0.6932 |
| s(*z*_l,s_) + s(*p_t_*_-1,s_) | 1 + *p_t_*_-1,s_\|*S* | 0.0716 | 0.6965 |
| s(*z*_l,s_) + *p_t_*_-1,s_ | 1 + *p_t_*_-1,s_\|*S* | 0.0716 | 0.6998 |
| *z*_l,s_ + *p_t_*_,s_ + *z*_l,s_ *p_t_*_,s_ | 0 + *p_t_*_,s_\|*S* | 0.0717 | 0.7031 |
| *z*_l,s_ + *p_t_*_,s_ + *w*_s_ + *p_t_*_,s_ *w*_s_ | 1 + *w*_s_\|*S* | 0.0717 | 0.7064 |
| *z*_l,s_ + *p_t_*_,s_ + *w*_s_ + *p_t_*_,s_ *w*_s_ | 1\|*S* | 0.0717 | 0.7097 |
| *z*_l,s_ + s(*p_t_*_,s_) + *w*_s_ | 0 + *w*_s_\|*S* | 0.0717 | 0.7130 |
| *z*_l,s_ + *p_t_*_,s_ + *w*_s_ | 0 + *w*_s_\|*S* | 0.0717 | 0.7163 |
| *z*_l,s_ + *p_t_*_,s_ + s(*w*_s_) | 0 + *w*_s_\|*S* | 0.0717 | 0.7196 |
| *z*_l,s_ + s(*p_t_*_,s_) + s(*w*_s_) | 0 + *w*_s_\|*S* | 0.0717 | 0.7229 |
| *z*_l,s_ + *p_t_*_,s_ + *w*_s_ + *p_t_*_,s_ *w*_s_ | 0 + *w*_s_\|*S* | 0.0717 | 0.7262 |
| s(*z*_l,s_) + *w*_s_ + *p_t_*_-1,s_ + *w*_s_ *p_t_*_-1,s_ | 0 + *w*_s_\|*S* | 0.0717 | 0.7295 |
| *z*_l,s_ + *w*_s_ + *p_t_*_-1,s_ | 0 + *w*_s_\|*S* | 0.0717 | 0.7328 |
| *z*_l,s_ + *p_t_*_,s_ | 1\|*S* | 0.0717 | 0.7361 |
| *z*_l,s_ + *p_t_*_,s_ + s(*w*_s_) | 1 + *w*_s_\|*S* | 0.0717 | 0.7394 |
| *z*_l,s_ + *p_t_*_,s_ + *w*_s_ | 1 + *w*_s_\|*S* | 0.0717 | 0.7427 |
| *z*_l,s_ + *p_t_*_,s_ + *w*_s_ | 1\|*S* | 0.0717 | 0.7460 |
| *z*_l,s_ + *p_t_*_,s_ + s(*w*_s_) | 1\|*S* | 0.0717 | 0.7492 |
| *z*_l,s_ + s(*p_t_*_,s_) + s(*w*_s_) | 1 + *w*_s_\|*S* | 0.0717 | 0.7525 |
| *z*_l,s_ + s(*p_t_*_,s_) + *w*_s_ | 1 + *w*_s_\|*S* | 0.0717 | 0.7558 |
| *z*_l,s_ + s(*w*_s_) | 0 + *w*_s_\|*S* | 0.0718 | 0.7590 |
| s(*z*_l,s_) + s(*p_t_*_,s_) + *w*_s_ | 0 + *p_t_*_,s_\|*S* | 0.0718 | 0.7622 |
| s(*z*_l,s_) + s(*p_t_*_,s_) + s(*w*_s_) | 0 + *p_t_*_,s_\|*S* | 0.0718 | 0.7655 |
| *z*_l,s_ + s(*p_t_*_,s_) | 1\|*S* | 0.0718 | 0.7687 |
| *z*_l,s_ + s(*p_t_*_,s_) + s(*w*_s_) | 1\|*S* | 0.0718 | 0.7719 |
| *z*_l,s_ + s(*p_t_*_,s_) + *w*_s_ | 1\|*S* | 0.0718 | 0.7751 |
| *z*_l,s_ + *p_t_*_,s_ | 0 + *p_t_*_,s_\|*S* | 0.0718 | 0.7783 |
| s(*z*_l,s_) + s(*p_t_*_,s_) + *w*_s_ | 1 + *p_t_*_,s_\|*S* | 0.0718 | 0.7815 |
| s(*z*_l,s_) + s(*p_t_*_,s_) + s(*w*_s_) | 1 + *p_t_*_,s_\|*S* | 0.0718 | 0.7847 |
| *z*_l,s_ + *w*_s_ | 0 + *w*_s_\|*S* | 0.0718 | 0.7879 |
| *z*_l,s_ + *p_t_*_,s_ | 1 + *z*_l,s_\|*S* | 0.0719 | 0.7910 |
| *z*_l,s_ + *p_t_*_,s_ + *w*_s_ + *p_t_*_,s_ *w*_s_ | 0 + *z*_l,s_\|*S* | 0.0719 | 0.7942 |
| *z*_l,s_ + *p_t_*_,s_ | 0 + *z*_l,s_\|*S* | 0.0719 | 0.7974 |
| *z*_l,s_ + *p_t_*_,s_ + *w*_s_ + *p_t_*_,s_ *w*_s_ | 1 + *z*_l,s_\|*S* | 0.0719 | 0.8005 |
| *z*_l,s_ + *p_t_*_,s_ + s(*w*_s_) | 0 + *z*_l,s_\|*S* | 0.0719 | 0.8037 |
| *z*_l,s_ + s(*p_t_*_,s_) | 0 + *z*_l,s_\|*S* | 0.0719 | 0.8068 |
| *z*_l,s_ + *p_t_*_,s_ + *w*_s_ | 0 + *z*_l,s_\|*S* | 0.0719 | 0.8100 |
| *z*_l,s_ + s(*p_t_*_,s_) + s(*w*_s_) | 0 + *z*_l,s_\|*S* | 0.0719 | 0.8132 |
| *z*_l,s_ + s(*p_t_*_,s_) + *w*_s_ | 0 + *z*_l,s_\|*S* | 0.0719 | 0.8163 |
| *z*_l,s_ + *p_t_*_,s_ + *w*_s_ | 1 + *z*_l,s_\|*S* | 0.0719 | 0.8194 |
| *z*_l,s_ + *p_t_*_,s_ + s(*w*_s_) | 1 + *z*_l,s_\|*S* | 0.0719 | 0.8226 |
| *z*_l,s_ + s(*p_t_*_,s_) | 1 + *z*_l,s_\|*S* | 0.0719 | 0.8257 |
| *z*_l,s_ | 1\|*S* | 0.0719 | 0.8289 |
| *z*_l,s_ + s(*w*_s_) | 1 + *w*_s_\|*S* | 0.0719 | 0.8320 |
| *z*_l,s_ + *w*_s_ | 1 + *w*_s_\|*S* | 0.0719 | 0.8351 |
| *z*_l,s_ + *w*_s_ | 1\|*S* | 0.0719 | 0.8383 |
| *z*_l,s_ + s(*w*_s_) | 1\|*S* | 0.0719 | 0.8414 |
| *z*_l,s_ + s(*p_t_*_,s_) + s(*w*_s_) | 1 + *z*_l,s_\|*S* | 0.0719 | 0.8445 |
| *z*_l,s_ + s(*p_t_*_,s_) + *w*_s_ | 1 + *z*_l,s_\|*S* | 0.0719 | 0.8477 |
| *z*_l,s_ + *p_t_*_,s_ + s(*w*_s_) +*z*_l,s_ *p_t_*_,s_ | 0 + *p_t_*_,s_\|*S* | 0.0719 | 0.8508 |
| *z*_l,s_ + *p_t_*_,s_ + *w*_s_ + *z*_l,s_ *p_t_*_,s_ | 0 + *p_t_*_,s_\|*S* | 0.0719 | 0.8539 |
| *z*_l,s_ + ws + *p_t_*_-1,s_ + *z*_l,s_ *w*_s_ | 1 + *w*_s_\|*S* | 0.0720 | 0.8570 |
| *z*_l,s_ + *w*_s_ + *z*_l,s_ *w*_s_ | 1 + *z*_l,s_\|*S* | 0.0720 | 0.8601 |
| s(*z*_l,s_) + s(*p_t_*_-1,s_) | 1 + *p_t_*_,s_\|*S* | 0.0720 | 0.8631 |
| *z*_l,s_ + *w*_s_ + *z*_l,s_ *w*_s_ | 0 + *z*_l,s_\|*S* | 0.0720 | 0.8662 |
| *z*_l,s_ | 1 + *z*_l,s_\|*S* | 0.0720 | 0.8692 |
| *z*_l,s_ | 0 + *z*_l,s_\|*S* | 0.0720 | 0.8723 |
| *z*_l,s_ + s(*w*_s_) | 0 + *z*_l,s_\|*S* | 0.0721 | 0.8753 |
| *z*_l,s_ + *w*_s_ | 0 + *z*_l,s_\|*S* | 0.0721 | 0.8784 |
| *z*_l,s_ + *w*_s_ | 1 + *z*_l,s_\|*S* | 0.0721 | 0.8814 |
| *z*_l,s_ + s(*w*_s_) | 1 + *z*_l,s_\|*S* | 0.0721 | 0.8844 |
| *z*_l,s_ + *w*_s_ + *p_t_*_-1,s_ + *z*_l,s_ *w*_s_ | 1\|*S* | 0.0721 | 0.8874 |
| *z*_l,s_ + *p_t_*_-1,s_ + *z*_l,s_ *p_t_*_-1,s_ | 1 + *p_t_*_-1,s_\|*S* | 0.0721 | 0.8904 |
| *z*_l,s_ + *p_t_*_-1,s_ | 0 + *z*_l,s_\|*S* | 0.0722 | 0.8934 |
| *z*_l,s_ + *w*_s_ + *p_t_*_-1,s_ + *z*_l,s_ *w*_s_ | 0 + *z*_l,s_\|*S* | 0.0722 | 0.8963 |
| *z*_l,s_ + s(*w*_s_) + *p_t_*_-1,s_ | 0 + *z*_l,s_\|*S* | 0.0722 | 0.8993 |
| *z*_l,s_ + *w*_s_ + *p_t_*_-1,s_ | 0 + *z*_l,s_\|*S* | 0.0722 | 0.9022 |
| s(*z*_l,s_) + *w*_s_ + *p_t_*_-1,s_ + *w*_s_ *p_t_*_-1,s_ | 1 + *z*_l,s_\|*S* | 0.0723 | 0.9051 |
| *z*_l,s_ + *p_t_*_,s_ + s(*w*_s_) | 0 + *p_t_*_,s_\|*S* | 0.0723 | 0.9079 |
| *z*_l,s_ + *p_t_*_,s_ + *w*_s_ | 0 + *p_t_*_,s_\|*S* | 0.0723 | 0.9108 |
| *z*_l,s_ + s(*w*_s_) + *p_t_*_-1,s_ | 1 + *z*_l,s_\|*S* | 0.0724 | 0.9136 |
| *z*_l,s_ + *w*_s_ + *p_t_*_-1,s_ | 1 + *z*_l,s_\|*S* | 0.0724 | 0.9165 |
| s(*z*_l,s_) + *p_t_*_-1,s_ | 1 + *z*_l,s_\|*S* | 0.0724 | 0.9193 |
| *z*_l,s_ + *w*_s_ + *p_t_*_-1,s_ + *z*_l,s_ *w*_s_ | 1 + *z*_l,s_\|*S* | 0.0724 | 0.9221 |
| *z*_l,s_ + *w*_s_ + *p_t_*_-1,s_ + *z*_l,s_ *w*_s_ | 0 + *z*_l,s_\|*S* | 0.0725 | 0.9249 |
| *z*_l,s_ + *p_t_*_,s_ + *w*_s_ + *p_t_*_,s_ *w*_s_ | 0 + *p_t_*_,s_\|*S* | 0.0725 | 0.9277 |
| s(*z*_l,s_) + *w*_s_ + *p_t_*_-1,s_ + *w*_s_ *p_t_*_-1,s_ | 1 + *w*_s_\|*S* | 0.0726 | 0.9304 |
| s(*z*_l,s_) + *w*_s_ + *p_t_*_-1,s_ + *w*_s_ *p_t_*_-1,s_ | 1\|*S* | 0.0726 | 0.9331 |
| *z*_l,s_ + *w*_s_ + *p_t_*_-1,s_ | 1 + *w*_s_\|*S* | 0.0726 | 0.9358 |
| *z*_l,s_ + s(*w*_s_) + *p_t_*_-1,s_ | 1 + *w*_s_\|*S* | 0.0726 | 0.9385 |
| *z*_l,s_ + *w*_s_ + *p_t_*_-1,s_ | 1\|*S* | 0.0726 | 0.9412 |
| *z*_l,s_ + s(*w*_s_) + *p_t_*_-1,s_ | 1\|*S* | 0.0726 | 0.9439 |
| *z*_l,s_ + *p_t_*_-1,s_ | 1\|*S* | 0.0727 | 0.9466 |
| *z*_l,s_ + *w*_s_ + *p_t_*_-1,s_ + *z*_l,s_ *w*_s_ | 0 + *p_t_*_-1,s_\|*S* | 0.0731 | 0.9490 |

**Table S3.** Best predictive models used in the construction of the inverse growth average model, where *z’*_l,s_ refers to individual standardized log-size at time *t*+1, *S* to the random effect of Site and function s() refers to a spline (i.e., non-linear model).

| **Model** | | **MRSME** | **Cumulative MRMSE weight** |
| --- | --- | --- | --- |
| **Fixed effect** | **Random effect** |  |  |
| s(*z’*_l,s_) | 1\|*S* | 0.0694 | 0.1896 |
| s(*z’*_l,s_) | 0 + *z’*_l,s_\|*S* | 0.0694 | 0.3785 |
| s(*z’*_l,s_) | 1 + *z’*_l,s_\|*S* | 0.0695 | 0.5651 |
| *z’*_l,s_ | 1\|*S* | 0.0705 | 0.7156 |
| *z’*_l,s_ | 0 + *z’*_l,s_\|*S* | 0.0707 | 0.8600 |

**Table S4.** Best predictive models used in the construction of the probability of recruitment average model, where *w* refers to soil water content at saturation, *p_t_* to the Palmer Drought Severity Index at time *t* , *p_t_*_-1_ Palmer Drought Severity Index at time *t*-1, *e_t_* to the El Niño-Southern Oscillation (ENSO) at time *t*, *e_t_*_-1_ to the El Niño-Southern Oscillation(ENSO) at time *t*-1, and *S* to the random effect of Site. For each variable, subscript s refers to standardization, and function s() refers to a spline (i.e., non-linear model).

| **Model** | | **MRSME** | **Cumulative MRSME weight** |
| --- | --- | --- | --- |
| **Fixed effect** | **Random effect** |  |  |
| s(*w*_s_) + *p_t_*_-1,s_ + s(*e_t_*_-1,s_) | 0 + *p_t_*_-1,s_\|*S* | 0.4662 | 0.0036 |
| s(*w*_s_) + s(*p_t_*_-1,s_) + s(*e_t_*_-1,s_) | 0 + *p_t_*_-1,s_\|*S* | 0.4662 | 0.0073 |
| s(*w*_s_) + s(*p_t_*_-1,s_) + s(*e_t_*_,s_) | 0 + *p_t_*_-1,s_\|*S* | 0.4669 | 0.0107 |
| s(*w*_s_) + *p_t_*_-1,s_ + s(*e_t_*_,s_) | 0 + *p_t_*_-1,s_\|*S* | 0.4669 | 0.0141 |
| s(*w*_s_) + s(*p_t_*_,s_) + s(*e_t_*_,s_) | 0 + *p_t_*_,s_\|*S* | 0.4677 | 0.0173 |
| s(*w*_s_) + *p_t_*_,s_ + s(*e_t_*_,s_) | 0 + *p_t_*_,s_\|*S* | 0.4677 | 0.0205 |
| s(*w*_s_) + *p_t_*_,s_ + s(*e_t_*_-1,s_) | 0 + *p_t_*_,s_\|*S* | 0.4680 | 0.0236 |
| s(*w*_s_) + s(*p_t_*_,s_) + s(*e_t_*_-1,s_) | 0 + *p_t_*_,s_\|*S* | 0.4680 | 0.0267 |
| s(*w*_s_) + *p_t_*_-1,s_ + *e_t_*_-1,s_ + *p_t_*_-1,s_ *e_t_*_-1,s_ | 0 + *p_t_*_-1,s_\|*S* | 0.4685 | 0.0297 |
| *w*_s_ + *p_t_*_-1,s_ + s(*e_t_*_-1,s_) + *w*_s_ *p_t_*_-1,s_ | 0 + *p_t_*_-1,s_\|*S* | 0.4688 | 0.0327 |
| s(*w*_s_) + s(*p_t_*_-1,s_) + s(*e_t_*_,s_) | 1 + *e_t_*_,s_\|*S* | 0.4688 | 0.0356 |
| s(*w*_s_) + *p_t_*_-1,s_ + s(*e_t_*_,s_) | 1 + *e_t_*_,s_\|*S* | 0.4688 | 0.0385 |
| s(*w*_s_) + *p_t_*_-1,s_ + *e_t_*_-1,s_ | 0 + *p_t_*_-1,s_\|*S* | 0.4690 | 0.0413 |
| s(*w*_s_) + s(*p_t_*_-1,s_) + *e_t_*_-1,s_ | 0 + *p_t_*_-1,s_\|*S* | 0.4690 | 0.0442 |
| *w*_s_ + *p_t_*_-1,s_ + s(*e_t_*_-1,s_) + *w*_s_ *p_t_*_-1,s_ | 1 + *w*_s_\|*S* | 0.4691 | 0.0471 |
| s(*w*_s_) + *p_t_*_,s_ + *e_t_*_-1,s_ + *p_t_*_,s_ *e_t_*_-1,s_ | 0 + *p_t_*_,s_\|*S* | 0.4692 | 0.0499 |
| *w*_s_ + *p_t_*_-1,s_ + s(*e_t_*_-1,s_) + *w*_s_ *p_t_*_-1,s_ | 1\|*S* | 0.4692 | 0.0527 |
| *w*_s_ + *p_t_*_-1,s_ + s(*e_t_*_-1,s_) + *w*_s_ *p_t_*_-1,s_ | 1 + *p_t_*_-1,s_\|*S* | 0.4692 | 0.0555 |
| *w*_s_ + *p_t_*_-1,s_ + s(*e_t_*_,s_) + *w*_s_ *p_t_*_-1,s_ | 0 + *p_t_*_-1,s_\|*S* | 0.4693 | 0.0583 |
| *w*_s_ + *p_t_*_-1,s_ + s(*e_t_*_-1,s_) + *w*_s_ *p_t_*_-1,s_ | 0 + *w*_s_\|*S* | 0.4694 | 0.0611 |
| *w*_s_ + *p_t_*_-1,s_ + s(*e_t_*_-1,s_) + *w*_s_ *p_t_*_-1,s_ | 1 + *e_t_*_-1,s_\|*S* | 0.4695 | 0.0638 |
| *w*_s_ + s(*p_t_*_-1,s_) + s(*e_t_*_-1,s_) | 0 + *p_t_*_-1,s_\|*S* | 0.4695 | 0.0666 |
| *w*_s_ + *p_t_*_-1,s_ + s(*e_t_*_-1,s_) | 0 + *p_t_*_-1,s_\|*S* | 0.4695 | 0.0693 |
| *w*_s_ + *p_t_*_-1,s_ + s(*e_t_*_,s_) + *w*_s_ *p_t_*_-1,s_ | 1 + *p_t_*_-1,s_\|*S* | 0.4697 | 0.0721 |
| *w*_s_ + *p_t_*_-1,s_ + s(*e_t_*_,s_) + *w*_s_ *p_t_*_-1,s_ | 1\|*S* | 0.4697 | 0.0748 |
| *w*_s_ + *p_t_*_-1,s_ + s(*e_t_*_-1,s_) + *w*_s_ *p_t_*_-1,s_ | 0 + *e_t_*_-1,s_\|*S* | 0.4697 | 0.0775 |
| s(*w*_s_) + *p_t_*_-1,s_ + *e_t_*_,s_ | 0 + *p_t_*_-1,s_\|*S* | 0.4697 | 0.0802 |
| s(*w*_s_) + s(*p_t_*_-1,s_) + *e_t_*_,s_ | 0 + *p_t_*_-1,s_\|*S* | 0.4697 | 0.0829 |
| s(*w*_s_) + *p_t_*_-1,s_ + s(*e_t_*_,s_) | 1 + *e_t_*_,s_\|*S* | 0.4697 | 0.0856 |
| s(*w*_s_) + s(*p_t_*_,s_) + s(*e_t_*_,s_) | 1 + *e_t_*_,s_\|*S* | 0.4697 | 0.0883 |
| *w*_s_ + *p_t_*_-1,s_ + s(*e_t_*_-1,s_) | 1 + *w*_s_\|*S* | 0.4697 | 0.0910 |
| *w*_s_ + s(*p_t_*_-1,s_) + s(*e_t_*_-1,s_) | 1 + *w*_s_\|*S* | 0.4697 | 0.0937 |
| s(*w*_s_) + *p_t_*_-1,s_ + *e_t_*_,s_ + *p_t_*_-1,s_ | 0 + *p_t_*_-1,s_\|*S* | 0.4698 | 0.0963 |
| s(*w*_s_) + *p_t_*_-1,s_ + s(*e_t_*_-1,s_) | 1\|*S* | 0.4699 | 0.0990 |
| *w*_s_ + *p_t_*_-1,s_ + s(*e_t_*_-1,s_) | 1\|*S* | 0.4699 | 0.1017 |
| *w*_s_ + s(*p_t_*_-1,s_) + s(*e_t_*_-1,s_) | 1\|*S* | 0.4699 | 0.1044 |
| s(*w*_s_) + s(*p_t_*_-1,s_) + s(*e_t_*_-1,s_) | 1\|*S* | 0.4699 | 0.1070 |
| *w*_s_ + *p_t_*_-1,s_ + s(*e_t_*_,s_) + *w*_s_ *p_t_*_-1,s_ | 0 + *w*_s_\|S | 0.4699 | 0.1097 |
| *w*_s_ + *p_t_*_-1,s_ + s(*e_t_*_,s_) + *w*_s_ *p_t_*_-1,s_ | 1 + *w*_s_\|*S* | 0.4699 | 0.1124 |
| s(*w*_s_) + s(*p_t_*_-1,s_) + s(*e_t_*_-1,s_) | 1 + *p_t_*_-1,s_\|*S* | 0.4700 | 0.1150 |
| *w*_s_ + *p_t_*_-1,s_ + s(*e_t_*_-1,s_) | 1 + *p_t_*_-1,s_\|*S* | 0.4700 | 0.1177 |
| *w*_s_ + s(*p_t_*_-1,s_) + s(*e_t_*_-1,s_) | 1 + *p_t_*_-1,s_\|*S* | 0.4700 | 0.1203 |
| s(*w*_s_) + *p_t_*_-1,s_ + s(*e_t_*_-1,s_) | 1 + *p_t_*_-1,s_\|*S* | 0.4700 | 0.1230 |
| s(*w*_s_) + s(*p_t_*_-1,s_) + s(*e_t_*_-1,s_) | 1 + *w*_s_\|*S* | 0.4700 | 0.1256 |
| s(*w*_s_) + *p_t_*_-1,s_ + s(*e_t_*_-1,s_) | 0 + *w*_s_\|*S* | 0.4700 | 0.1283 |
| *w*_s_ + s(*p_t_*_-1,s_) + s(*e_t_*_-1,s_) | 0 + *w*_s_\|*S* | 0.4700 | 0.1309 |
| s(*w*_s_) + s(*p_t_*_-1,s_) + s(*e_t_*_-1,s_) | 0 + *w*_s_\|*S* | 0.4700 | 0.1335 |
| s(*w*_s_) + *p_t_*_-1,s_ + s(*e_t_*_-1,s_) | 1 + *w*_s_\|*S* | 0.4700 | 0.1362 |
| *w*_s_ + *p_t_*_-1,s_ + s(*e_t_*_-1,s_) | 0 + *w*_s_\|*S* | 0.4700 | 0.1388 |
| *w*_s_ + *p_t_*_-1,s_ + s(*e_t_*_,s_) | 0 + *p_t_*_-1,s_\|*S* | 0.4700 | 0.1415 |
| *w*_s_ + s(*p_t_*_-1,s_) + s(*e_t_*_,s_) | 0 + *p_t_*_-1,s_\|*S* | 0.4700 | 0.1441 |
| *w*_s_ + s(*p_t_*_-1,s_) + s(*e_t_*_-1,s_) | 1 + *e_t_*_-1,s_\|*S* | 0.4701 | 0.1467 |
| *w*_s_ + *p_t_*_-1,s_ + s(*e_t_*_-1,s_) | 1 + *e_t_*_-1,s_\|*S* | 0.4701 | 0.1493 |
| s(*w*_s_) + *p_t_*_-1,s_ + s(*e_t_*_-1,s_) | 1 + *e_t_*_-1,s_\|*S* | 0.4701 | 0.1520 |
| s(*w*_s_) + s(*p_t_*_-1,s_) + s(*e_t_*_-1,s_) | 1 + *e_t_*_-1,s_\|*S* | 0.4701 | 0.1546 |
| *w*_s_ + *p_t_*_-1,s_ + s(*e_t_*_,s_) + *w*_s_ *p_t_*_-1,s_ | 1 + *e_t_*_,s_\|*S* | 0.4701 | 0.1572 |
| *w*_s_ + s(*p_t_*_-1,s_) + s(*e_t_*_-1,s_) | 1 + *w*_s_\|*S* | 0.4702 | 0.1598 |
| *w*_s_ + *p_t_*_-1,s_ + s(*e_t_*_,s_) + *w*_s_ *p_t_*_-1,s_ | 0 + *e_t_*_,s_\|*S* | 0.4702 | 0.1624 |
| s(*w*_s_) + s(*p_t_*_-1,s_) + s(*e_t_*_,s_) | 1 + *w*_s_\|*S* | 0.4702 | 0.1650 |
| *w*_s_ + *p_t_*_-1,s_ + s(*e_t_*_,s_) | 1 + *w*_s_*\|S* | 0.4702 | 0.1676 |
| s(*w*_s_) + s(*p_t_*_-1,s_) + s(*e_t_*_,s_) | 1\|*S* | 0.4703 | 0.1702 |
| s(*w*_s_) + *p_t_*_-1,s_ + s(*e_t_*_,s_) | 1\|*S* | 0.4703 | 0.1728 |
| *w*_s_ + *p_t_*_-1,s_ + s(*e_t_*_,s_) | 1\|*S* | 0.4703 | 0.1753 |
| *w*_s_ + s(*p_t_*_-1,s_) + s(*e_t_*_,s_) | 1\|*S* | 0.4703 | 0.1779 |
| *w*_s_ + s(*p_t_*_-1,s_) + s(*e_t_*_-1,s_) | 0 + *e_t_*_-1,s_\|*S* | 0.4704 | 0.1805 |
| s(*w*_s_) + *p_t_*_-1,s_ + s(*e_t_*_-1,s_) | 0 + *e_t_*_-1,s_\|*S* | 0.4704 | 0.1830 |
| *w*_s_ + *p_t_*_-1,s_ + s(*e_t_*_-1,s_) | 0 + *e_t_*_-1,s_\|*S* | 0.4704 | 0.1856 |
| s(*w*_s_) + s(*p_t_*_-1,s_) + s(*e_t_*_-1,s_) | 0 + *e_t_*_-1,s_\|*S* | 0.4704 | 0.1881 |
| s(*w*_s_) + s(*p_t_*_-1,s_) + s(*e_t_*_,s_) | 1 + *p_t_*_-1,s_\|*S* | 0.4704 | 0.1907 |
| s(*w*_s_) + *p_t_*_-1,s_ + s(*e_t_*_,s_) | 1 + *p_t_*_-1,s_\|*S* | 0.4704 | 0.1932 |
| *w*_s_ + s(*p_t_*_-1,s_) + s(*e_t_*_,s_) | 1 + *p_t_*_-1,s_\|*S* | 0.4704 | 0.1958 |
| *w*_s_ + *p_t_*_-1,s_ + s(*e_t_*_,s_) | 1 + *p_t_*_-1,s_\|*S* | 0.4704 | 0.1983 |
| s(*w*_s_) + *p_t_*_-1,s_ + s(*e_t_*_,s_) | 1 + *w*_s_\|*S* | 0.4704 | 0.2009 |
| *w*_s_ + s(*p_t_*_-1,s_) + s(*e_t_*_,s_) | 0 + *w*_s_\|*S* | 0.4704 | 0.2034 |
| s(*w*_s_) + *p_t_*_-1,s_ + s(*e_t_*_,s_) | 0 + *w*_s_\|*S* | 0.4704 | 0.2060 |
| *w*_s_ + *p_t_*_-1,s_ + s(*e_t_*_,s_) | 0 + *w*_s_\|*S* | 0.4704 | 0.2085 |
| s(*w*_s_) + s(*p_t_*_-1,s_) + s(*e_t_*_,s_) | 0 + *w*_s_\|*S* | 0.4704 | 0.2110 |
| s(*w*_s_) + s(*p_t_*_,s_) + *e_t_*_,s_ | 0 + *p_t_*_,s_\|*S* | 0.4704 | 0.2136 |
| s(*w*_s_) + *p_t_*_,s_ + *e_t_*_,s_ | 0 + *p_t_*_,s_\|*S* | 0.4704 | 0.2161 |
| s(ws) + *p_t_*_,s_ + *e_t_*_,s_ + *p_t_*_,s_ *e_t_*_,s_ | 0 + *p_t_*_,s_\|*S* | 0.4706 | 0.2187 |
| s(*w*_s_) + *p_t_*_,s_ + *e_t_*_-1,s_ | 0 + *p_t_*_,s_\|*S* | 0.4707 | 0.2211 |
| ss(*w*_s_) + s(*p_t_*_,s_) + *e_t_*_,s_ | 0 + *p_t_*_,s_\|*S* | 0.4707 | 0.2236 |
| *w*_s_ + *p_t_*_,s_ + s(*e_t_*_,s_) + *w*_s_ *p_t_*_,s_ | 0 + *p_t_*_,s_\|*S* | 0.4707 | 0.2261 |
| *w*_s_ + *p_t_*_-1,s_ + s(*e_t_*_,s_) | 0 + *p_t_*_,s_\|*S* | 0.4708 | 0.2286 |
| *w*_s_ + s(*p_t_*_,s_) + s(*e_t_*_,s_) | 0 + *p_t_*_,s_\|*S* | 0.4708 | 0.2311 |
| *w*_s_ + s(*p_t_*_-1,s_) + s(*e_t_*_,s_) | 1 + *e_t_*_,s_\|*S* | 0.4708 | 0.2335 |
| *w*_s_ + *p_t_*_-1,s_ + s(*e_t_*_,s_) | 1 + *e_t_*_,s_\|*S* | 0.4708 | 0.2360 |
| *w*_s_ + *p_t_*_-1,s_ + s(*e_t_*_,s_) | 0 + *e_t_*_,s_\|*S* | 0.4709 | 0.2385 |
| s(*w*_s_) + *p_t_*_-1,s_ + s(*e_t_*_,s_) | 0 + *e_t_*_,s_\|*S* | 0.4709 | 0.2409 |
| s(*w*_s_) + s(*p_t_*_-1,s_) + s(*e_t_*_,s_) | 0 + *e_t_*_,s_\|*S* | 0.4709 | 0.2434 |
| *w*_s_ + s(*p_t_*_-1,s_) + s(*e_t_*_,s_) | 0 + *e_t_*_,s_\|*S* | 0.4709 | 0.2458 |
| *w*_s_ + *p_t_*_-1,s_ + *e_t_*_-1,s_ +*w*_s_ *p_t_*_-1,s_ + *w*_s_ *e_t_*_-1,s_ + *p_t_*_-1,s_ *e_t_*_-1,s_ | 0 + *p_t_*_-1,s_\|*S* | 0.4709 | 0.2483 |
| *w*_s_ + *p_t_*_,s_ + s(*e_t_*_,s_) + *w*_s_ *p_t_*_,s_ | 1 + *w*_s_\|*S* | 0.4709 | 0.2507 |
| s(*w*_s_) + s(*p_t_*_,s_) + s(*e_t_*_,s_) | 1 + *w*_s_\|*S* | 0.4710 | 0.2531 |
| *w*_s_ + *p_t_*_,s_ + s(*e_t_*_,s_) | 1 + *w*_s_\|*S* | 0.4710 | 0.2556 |
| *w*_s_ + *p_t_*_,s_ + s(*e_t_*_,s_) + *w*_s_ *p_t_*_,s_ | 1 + *p_t_*_,s_\|*S* | 0.4710 | 0.2580 |
| *w*_s_ + *p_t_*_,s_ + s(*e_t_*_,s_) + *w*_s_ *p_t_*_,s_ | 1\|*S* | 0.4710 | 0.2604 |
| s(*w*_s_) + *p_t_*_,s_ + s(*e_t_*_,s_) | 1\|*S* | 0.4711 | 0.2628 |
| *w*_s_ + s(*p_t_*_,s_) + s(*e_t_*_,s_) | 1\|*S* | 0.4711 | 0.2652 |
| *w*_s_ + *p_t_*_,s_ + s(*e_t_*_,s_) | 1\|*S* | 0.4711 | 0.2677 |
| s(*w*_s_) + s(*p_t_*_,s_) + s(*e_t_*_,s_) | 1\|*S* | 0.4711 | 0.2701 |
| s(*w*_s_) + s(*p_t_*_,s_) + s(*e_t_*_,s_) | 1 + *p_t_*_,s_\|*S* | 0.4711 | 0.2725 |
| s(*w*_s_) + *p_t_*_,s_ + s(*e_t_*_,s_) | 1 + *p_t_*_,s_\|*S* | 0.4711 | 0.2749 |
| *w*_s_ + *p_t_*_,s_ + s(*e_t_*_,s_) | 1 + *p_t_*_,s_\|*S* | 0.4711 | 0.2773 |
| *w*_s_ + s(*p_t_*_,s_) + s(*e_t_*_,s_) | 1 + *p_t_*_,s_\|*S* | 0.4711 | 0.2797 |
| *w*_s_ + s(*p_t_*_,s_) + s(*e_t_*_-1,s_) | 0 + *p_t_*_,s_\|*S* | 0.4711 | 0.2821 |
| *w*_s_ + *p_t_*_,s_ + s(*e_t_*_-1,s_) | 0 + *p_t_*_,s_\|*S* | 0.4711 | 0.2845 |
| *w*_s_ + *p_t_*_,s_ + s(*e_t_*_-1,s_) + *w*_s_ *p_t_*_,s_ | 0 + *p_t_*_,s_\|*S* | 0.4712 | 0.2869 |
| *w*_s_ + *p_t_*_,s_ + s(*e_t_*_,s_) + *w*_s_ *p_t_*_,s_ | 0 + *w*_s_\|*S* | 0.4712 | 0.2893 |
| *w*_s_ + s(*p_t_*_,s_) + s(*e_t_*_,s_) | 0 + *w*_s_\|*S* | 0.4712 | 0.2917 |
| *w*_s_ + s(*p_t_*_,s_) + s(*e_t_*_,s_) | 1 + *w*_s_\|*S* | 0.4712 | 0.2941 |
| s(*w*_s_) + *p_t_*_,s_ + s(*e_t_*_,s_) | 1 + *w*_s_\|*S* | 0.4712 | 0.2965 |
| *w*_s_ + *p_t_*_,s_ + s(*e_t_*_,s_) | 0 + *w*_s_\|*S* | 0.4712 | 0.2988 |
| s(*w*_s_) + *p_t_*_,s_ + s(*e_t_*_,s_) | 0 + *w*_s_\|*S* | 0.4712 | 0.3012 |
| s(*w*_s_) + s(*p_t_*_,s_) + s(*e_t_*_,s_) | 0 + *w*_s_\|*S* | 0.4712 | 0.3036 |
| *w*_s_ + *p_t_*_-1,s_ + *e_t_*_-1,s_ + *w*_s_ *p_t_*_-1,s_ + *w*_s_ *e_t_*_-1,s_ + *p_t_*_-1,s_ | 0 + *e_t_*_-1,s_\|*S* | 0.4713 | 0.3060 |
| *w*_s_ + *p_t_*_,s_ + *e_t_*_-1,s_ +*w*_s_ *p_t_*_,s_ + *w*_s_ *e_t_*_-1,s_ + *p_t_*_,s_ *e_t_*_-1,s_ | 0 + *p_t_*_,s_\|*S* | 0.4714 | 0.3083 |
| *w*_s_ + *p_t_*_,s_ + s(*e_t_*_-1,s_) + *w*_s_ *p_t_*_,s_ | 1 + *p_t_*_,s_\|*S* | 0.4715 | 0.3107 |
| *w*_s_ + *p_t_*_,s_ + s(*e_t_*_-1,s_) + *w*_s_ *p_t_*_,s_ | 1\|*S* | 0.4715 | 0.3130 |
| *w*_s_ + *p_t_*_,s_ + s(*e_t_*_-1,s_) | 1\|*S* | 0.4715 | 0.3154 |
| s(*w*_s_) + *p_t_*_,s_ + s(*e_t_*_-1,s_) | 1\|*S* | 0.4715 | 0.3177 |
| *w*_s_ + s(*p_t_*_,s_) + s(*e_t_*_-1,s_) | 1\|*S* | 0.4715 | 0.3200 |
| s(*w*_s_) + *p_t_*_,s_ + s(*e_t_*_-1,s_) | 1 + *p_t_*_,s_\|*S* | 0.4715 | 0.3223 |
| *w*_s_ + s(*p_t_*_,s_) + s(*e_t_*_-1,s_) | 1 + *p_t_*_,s_\|*S* | 0.4715 | 0.3247 |
| *w*_s_ + *p_t_*_,s_ + s(*e_t_*_-1,s_) | 1 + *p_t_*_,s_\|*S* | 0.4715 | 0.3270 |
| s(*w*_s_) + s(*p_t_*_,s_) + s(*e_t_*_-1,s_) | 1 + *p_t_*_,s_\|*S* | 0.4715 | 0.3293 |
| s(*w*_s_) + s(*p_t_*_,s_) + s(*e_t_*_-1,s_) | 1\|*S* | 0.4715 | 0.3317 |
| *w*_s_ + *p_t_*_-1,s_ + *e_t_*_-1,s_ +*w*_s_ *p_t_*_-1,s_ + *w*_s_ *e_t_*_-1,s_ + *p_t_*_-1,s_ *e_t_*_-1,s_ | 1\|*S* | 0.4715 | 0.3340 |
| *w*_s_ + *p_t_*_-1,s_ + *e_t_*_-1,s_ + *w*_s_ *p_t_*_-1,s_ | 0 + *p_t_*_-1,s_\|*S* | 0.4715 | 0.3363 |
| *w*_s_ + *p_t_*_-1,s_ + *e_t_*_-1,s_ +*w*_s_ *p_t_*_-1,s_ + *w*_s_ *e_t_*_-1,s_ + *p_t_*_-1,s_ *e_t_*_-1,s_ | 1 + *p_t_*_-1,s_\|*S* | 0.4715 | 0.3386 |
| *w*_s_ + *p_t_*_,s_ + s(*e_t_*_-1,s_) + *w*_s_ *p_t_*_,s_ | 0 + ws\|*S* | 0.4716 | 0.3410 |
| *w*_s_ + *p_t_*_,s_ + s(*e_t_*_-1,s_) + *w*_s_ *p_t_*_,s_ | 1 + *w*_s_\|*S* | 0.4716 | 0.3433 |
| *w*_s_ + *p_t_*_,s_ + s(*e_t_*_,s_) + *w*_s_ *p_t_*_,s_ | 1 + *e_t_*_,s_\|*S* | 0.4716 | 0.3456 |
| *w*_s_ + *p_t_*_-1,s_ + *e_t_*_,s_ +*w*_s_ *p_t_*_-1,s_ + *w*_s_ *e_t_*_,s_ + *p_t_*_-1,s_ *e_t_*_,s_ | 0 + *p_t_*_-1,s_\|*S* | 0.4716 | 0.3479 |
| s(*w*_s_) + *p_t_*_-1,s_ + *e_t_*_,s_ | 1 + *e_t_*_,s_\|*S* | 0.4716 | 0.3502 |
| s(*w*_s_) + s(*p_t_*_-1,s_) + *e_t_*_,s_ | 1 + *e_t_*_,s_\|*S* | 0.4716 | 0.3526 |
| *w*_s_ + *p_t_*_-1,s_ + *e_t_*_,s_ +*w*_s_ *p_t_*_-1,s_ + *w*_s_ *e_t_*_,s_ + *p_t_*_-1,s_ *e_t_*_,s_ | 0 + *e_t_*_,s_\|*S* | 0.4716 | 0.3549 |
| s(*w*_s_) + s(*p_t_*_,s_) + s(*e_t_*_-1,s_) | 0 + *w*_s_\|*S* | 0.4716 | 0.3572 |
| s(*w*_s_) + s(*p_t_*_,s_) + s(*e_t_*_-1,s_) | 1 + *w*_s_\|*S* | 0.4716 | 0.3595 |
| *w*_s_ + s(*p_t_*_,s_) + s(*e_t_*_-1,s_) | 1 + *w*_s_\|*S* | 0.4716 | 0.3618 |
| *w*_s_ + *p_t_*_,s_ + s(*e_t_*_-1,s_) | 1 + *w*_s_\|*S* | 0.4716 | 0.3641 |
| s(*w*_s_) + *p_t_*_,s_ + s(*e_t_*_-1,s_) | 1 + *w*_s_\|*S* | 0.4716 | 0.3664 |
| s(*w*_s_) + *p_t_*_,s_ + s(*e_t_*_-1,s_) | 0 + *w*_s_\|*S* | 0.4716 | 0.3688 |
| *w*_s_ + s(*p_t_*_,s_) + s(*e_t_*_-1,s_) | 0 + *w*_s_\|*S* | 0.4716 | 0.3711 |
| *w*_s_ + *p_t_*_,s_ + s(*e_t_*_-1,s_) | 0 + *w*_s_\|*S* | 0.4716 | 0.3734 |
| *w*_s_ + *p_t_*_,s_ + s(*e_t_*_-1,s_) + *w*_s_ *p_t_*_,s_ | 1 + *e_t_*_-1,s_\|*S* | 0.4716 | 0.3757 |
| *w*_s_ + *p_t_*_-1,s_ + *e_t_*_-1,s_ +*w*_s_ *p_t_*_-1,s_ + *w*_s_ *e_t_*_-1,s_ + *p_t_*_-1,s_ *e_t_*_-1,s_ | 1 + *w*_s_\|*S* | 0.4716 | 0.3780 |
| *w*_s_ + s(*p_t_*_,s_) + s(*e_t_*_-1,s_) | 1 + *e_t_*_-1,s_\|*S* | 0.4716 | 0.3803 |
| *w*_s_ + *p_t_*_,s_ + s(*e_t_*_-1,s_) | 1 + *e_t_*_-1,s_\|*S* | 0.4716 | 0.3827 |
| s(*w*_s_) + s(*p_t_*_,s_) + s(*e_t_*_-1,s_) | 1 + *e_t_*_-1,s_\|*S* | 0.4716 | 0.3850 |
| s(*w*_s_) + *p_t_*_,s_ + s(*e_t_*_-1,s_) | 1 + *e_t_*_-1,s_\|*S* | 0.4716 | 0.3873 |
| *w*_s_ + s(*p_t_*_,s_) + s(*e_t_*_,s_) | 1 + *e_t_*_,s_\|*S* | 0.4716 | 0.3896 |
| *w*_s_ + *p_t_*_,s_ + s(*e_t_*_,s_) + *w*_s_ *p_t_*_,s_ | 0 + *e_t_*_,s_\|*S* | 0.4716 | 0.3919 |
| *w*_s_ + *p_t_*_,s_ + s(*e_t_*_,s_) | 1 + *e_t_*_,s_\|*S* | 0.4716 | 0.3942 |
| *w*_s_ + *p_t_*_,s_ + *e_t_*_-1,s_ +*w*_s_ *p_t_*_,s_ + *w*_s_ *e_t_*_-1,s_ + *p_t_*_,s_ *e_t_*_-1,s_ | 0 + *e_t_*_-1,s_\|*S* | 0.4716 | 0.3965 |
| *w*_s_ + *p_t_*_-1,s_ + *e_t_*_-1,s_ +*w*_s_ *p_t_*_-1,s_ + *w*_s_ *e_t_*_-1,s_ + *p_t_*_-1,s_ *e_t_*_-1,s_ | 1 + *e_t_*_-1,s_\|*S* | 0.4717 | 0.3988 |
| *w*_s_ + *p_t_*_-1,s_ + *e_t_*_-1,s_ +*w*_s_ *p_t_*_-1,s_ + *w*_s_ *e_t_*_-1,s_ + *p_t_*_-1,s_ *e_t_*_-1,s_ | 0 + *p_t_*_-1,s_\|*S* | 0.4717 | 0.4011 |
| s(*w*_s_) + s(*p_t_*_,s_) + s(*e_t_*_,s_) | 0 + *e_t_*_,s_\|*S* | 0.4717 | 0.4034 |
| s(*w*_s_) + *p_t_*_,s_ + s(*e_t_*_,s_) | 0 + *e_t_*_,s_\|*S* | 0.4717 | 0.4057 |
| *w*_s_ + s(*p_t_*_,s_) + s(*e_t_*_,s_) | 0 + *e_t_*_,s_\|*S* | 0.4717 | 0.4080 |
| *w*_s_ + *p_t_*_,s_ + s(*e_t_*_,s_) | 0 + *e_t_*_,s_\|*S* | 0.4717 | 0.4103 |
| *w*_s_ + *p_t_*_-1,s_ + *e_t_*_-1,s_ + *w*_s_ *p_t_*_-1,s_ | 1 + *w*_s_\|*S* | 0.4719 | 0.4125 |
| *w*_s_ + *p_t_*_-1,s_ + *e_t_*_-1,s_ +*w*_s_ *p_t_*_-1,s_ + *w*_s_ *e_t_*_-1,s_ + *p_t_*_-1,s_ *e_t_*_-1,s_ | 0 + *w*_s_\|*S* | 0.4719 | 0.4148 |
| *w*_s_ + *p_t_*_-1,s_ + *e_t_*_-1,s_ + *p_t_*_-1,s_ *e_t_*_-1,s_ | 1 + *w*_s_\|*S* | 0.4719 | 0.4171 |
| *w*_s_ + *p_t_*_-1,s_ + *e_t_*_,s_ +*w*_s_ *p_t_*_-1,s_ + *w*_s_ *e_t_*_,s_ + *p_t_*_-1,s_ *e_t_*_,s_ | 1 + *w*_s_\|*S* | 0.4719 | 0.4193 |
| *w*_s_ + *p_t_*_-1,s_ + *e_t_*_-1,s_ + *w*_s_ *p_t_*_-1,s_ | 1\|*S* | 0.4719 | 0.4216 |
| *w*_s_ + *p_t_*_-1,s_ + *e_t_*_-1,s_ + *w*_s_ *p_t_*_-1,s_ | 1 + *p_t_*_-1,s_\|*S* | 0.4719 | 0.4238 |
| *w*_s_ + *p_t_*_,s_ + *e_t_*_,s_ +*w*_s_ *p_t_*_,s_ + *w*_s_ *e_t_*_,s_ + *p_t_*_,s_ *e_t_*_,s_ | 1\|*S* | 0.4719 | 0.4261 |
| *w*_s_ + *p_t_*_-1,s_ + *e_t_*_,s_ +*w*_s_ *p_t_*_-1,s_ + *w*_s_ *e_t_*_,s_ + *p_t_*_-1,s_ *e_t_*_,s_ | 1 + *p_t_*_-1,s_\|*S* | 0.4720 | 0.4283 |
| *w*_s_ + *p_t_*_,s_ + *e_t_*_-1,s_ +*w*_s_ *p_t_*_,s_ + *w*_s_ *e_t_*_-1,s_ + *p_t_*_,s_ *e_t_*_-1,s_ | 1 + *w*_s_\|*S* | 0.4720 | 0.4305 |
| *w*_s_ + *p_t_*_,s_ + s(*e_t_*_-1,s_) + *w*_s_ *p_t_*_,s_ | 0 + *e_t_*_-1,s_\|*S* | 0.4720 | 0.4328 |
| *w*_s_ + *p_t_*_,s_ + *e_t_*_-1,s_ +*w*_s_ *p_t_*_,s_ + *w*_s_ *e_t_*_-1,s_ + *p_t_*_,s_ *e_t_*_-1,s_ | 1 + *p_t_*_,s_\|*S* | 0.4720 | 0.4350 |
| *w*_s_ + *p_t_*_,s_ + *e_t_*_-1,s_ +*w*_s_ *p_t_*_,s_ + *w*_s_ *e_t_*_-1,s_ + *p_t_*_,s_ *e_t_*_-1,s_ | 1\|*S* | 0.4720 | 0.4373 |
| s(*w*_s_) + *p_t_*_-1,s_ + *e_t_*_-1,s_ +s(*w*_s_) *p_t_*_-1,s_ + s(*w*_s_) *e_t_*_-1,s_ + *p_t_*_-1,s_ *e_t_*_-1,s_ | 1\|*S* | 0.4720 | 0.4395 |
| *w*_s_ + *p_t_*_-1,s_ + *e_t_*_-1,s_ + *p_t_*_-1,s_ *e_t_*_-1,s_ | 1\|*S* | 0.4720 | 0.4417 |
| s(*w*_s_) + *p_t_*_,s_ + s(*e_t_*_-1,s_) | 0 + *e_t_*_-1,s_\|*S* | 0.4720 | 0.4440 |
| *w*_s_ + s(*p_t_*_,s_) + s(*e_t_*_-1,s_) | 0 + *e_t_*_-1,s_\|*S* | 0.4720 | 0.4462 |
| s(*w*_s_) + s(*p_t_*_,s_) + s(*e_t_*_-1,s_) | 0 + *e_t_*_-1,s_\|*S* | 0.4720 | 0.4484 |
| *w*_s_ + *p_t_*_,s_ + s(*e_t_*_-1,s_) | 0 + *e_t_*_-1,s_\|*S* | 0.4720 | 0.4507 |
| s(*w*_s_) + *p_t_*_-1,s_ + *e_t_*_,s_ + *p_t_*_-1,s_ *e_t_*_,s_ | 1 + *e_t_*_,s_\|*S* | 0.4721 | 0.4529 |
| *w*_s_ + *p_t_*_-1,s_ + *e_t_*_-1,s_ + *w*_s_ *p_t_*_-1,s_ | 1 + *e_t_*_-1,s_\|*S* | 0.4721 | 0.4551 |
| *w*_s_ + *p_t_*_-1,s_ + *e_t_*_,s_ + *w*_s_ *p_t_*_-1,s_ | 0 + *p_t_*_-1,s_\|*S* | 0.4721 | 0.4573 |
| *w*_s_ + *p_t_*_-1,s_ + *e_t_*_-1,s_ + *w*_s_ *p_t_*_-1,s_ | 0 + *e_t_*_-1,s_\|*S* | 0.4721 | 0.4595 |
| s(*w*_s_) + *p_t_*_-1,s_ + *e_t_*_-1,s_ + *p_t_*_-1,s_ *e_t_*_-1,s_ | 1 + *p_t_*_-1,s_\|*S* | 0.4721 | 0.4617 |
| *w*_s_ + *p_t_*_-1,s_ + *e_t_*_-1,s_ + *p_t_*_-1,s_ *e_t_*_-1,s_ | 1 + *p_t_*_-1,s_\|*S* | 0.4721 | 0.4640 |
| *w*_s_ + s(*p_t_*_-1,s_) + *e_t_*_-1,s_ | 0 + *p_t_*_-1,s_\|*S* | 0.4721 | 0.4662 |
| *w*_s_ + *p_t_*_-1,s_ + *e_t_*_-1,s_ | 0 + *p_t_*_-1,s_\|*S* | 0.4721 | 0.4684 |
| s(*w*_s_) + *p_t_*_-1,s_ + *e_t_*_-1,s_ + *p_t_*_-1,s_ *e_t_*_-1,s_ | 1 + *w*_s_\|*S* | 0.4721 | 0.4706 |
| s(*w*_s_) + *p_t_*_-1,s_ + *e_t_*_-1,s_ + *p_t_*_-1,s_ *e_t_*_-1,s_ | 0 + *w*_s_\|*S* | 0.4721 | 0.4728 |
| *w*_s_ + *p_t_*_-1,s_ + *e_t_*_-1,s_ + *p_t_*_-1,s_ *e_t_*_-1,s_ | 0 + *w*_s_\|*S* | 0.4721 | 0.4750 |
| *w*_s_ + *p_t_*_-1,s_ + *e_t_*_-1,s_ + *w*_s_ *p_t_*_-1,s_ | 0 + *w*_s_\|*S* | 0.4722 | 0.4772 |
| *w*_s_ + *p_t_*_-1,s_ + *e_t_*_-1,s_ + *p_t_*_-1,s_ *e_t_*_-1,s_ | 1 + *e_t_*_-1,s_\|*S* | 0.4722 | 0.4794 |
| s(*w*_s_) + *p_t_*_-1,s_ + *e_t_*_-1,s_ + *p_t_*_-1,s_ *e_t_*_-1,s_ | 1 + *e_t_*_-1,s_\|*S* | 0.4722 | 0.4816 |
| *w*_s_ + *p_t_*_-1,s_ + *e_t_*_,s_ + *w*_s_ *p_t_*_-1,s_ + *w*_s_ *e_t_*_,s_ + *p_t_*_-1,s_ *e_t_*_,s_ | 0 + *w*_s_\|*S* | 0.4722 | 0.4838 |
| *w*_s_ + *p_t_*_,s_ + *e_t_*_-1,s_ + *w*_s_ *p_t_*_,s_ + *w*_s_ *e_t_*_-1,s_ + *p_t_*_,s_ *e_t_*_-1,s_ | 0 + *w*_s_\|*S* | 0.4722 | 0.4860 |
| *w*_s_ + *p_t_*_,s_ + *e_t_*_-1,s_ + *w*_s_ *p_t_*_,s_ + *w*_s_ *e_t_*_-1,s_ + *p_t_*_,s_ *e_t_*_-1,s_ | 1 + *e_t_*_-1,s_\|*S* | 0.4723 | 0.4882 |
| *w*_s_ + *p_t_*_-1,s_ + *e_t_*_-1,s_ + *p_t_*_-1,s_ *e_t_*_-1,s_ | 0 + *e_t_*_-1,s_\|*S* | 0.4723 | 0.4904 |
| s(*w*_s_) + *p_t_*_-1,s_ + *e_t_*_-1,s_ + *p_t_*_-1,s_ *e_t_*_-1,s_ | 0 + *e_t_*_-1,s_\|*S* | 0.4723 | 0.4926 |
| *w*_s_ + *p_t_*_-1,s_ + *e_t_*_,s_ + *w*_s_ *p_t_*_-1,s_ | 1 + *w*_s_\|*S* | 0.4724 | 0.4947 |
| s(*w*_s_) + *p_t_*_-1,s_ + *e_t_*_-1,s_ | 1 + *w*_s_\|*S* | 0.4724 | 0.4969 |
| *w*_s_ + *p_t_*_-1,s_ + *e_t_*_-1,s_ | 1 + *w*_s_\|*S* | 0.4724 | 0.4990 |
| *w*_s_ + s(*p_t_*_-1,s_) + *e_t_*_-1,s_ | 1 + *w*_s_\|*S* | 0.4724 | 0.5012 |
| s(*w*_s_) + *p_t_*_,s_ + *e_t_*_,s_ | 1 + *e_t_*_,s_\|*S* | 0.4724 | 0.5033 |
| s(*w*_s_) + s(*p_t_*_,s_) + (*e_t_*_,s_) | 1 + *e_t_*_,s_\|*S* | 0.4724 | 0.5055 |
| *w*_s_ + *p_t_*_,s_ + *e_t_*_-1,s_ + *p_t_*_,s_ *e_t_*_-1,s_ | 0 + *p_t_*_,s_\|*S* | 0.4725 | 0.5077 |
| *w*_s_ + *p_t_*_-1,s_ + *e_t_*_,s_ + *w*_s_ *p_t_*_-1,s_ | 1\|*S* | 0.4725 | 0.5098 |
| *w*_s_ + *p_t_*_-1,s_ + *e_t_*_,s_ + *w*_s_ *p_t_*_-1,s_ | 1 + *p_t_*_-1,s_\|*S* | 0.4725 | 0.5120 |
| *w*_s_ + *p_t_*_-1,s_ + *e_t_*_-1,s_ | 1\|*S* | 0.4725 | 0.5141 |
| s(*w*_s_) + *p_t_*_-1,s_ + *e_t_*_-1,s_ | 1\|*S* | 0.4725 | 0.5162 |
| s(*w*_s_) + s(*p_t_*_-1,s_) + *e_t_*_-1,s_ | 1\|*S* | 0.4725 | 0.5184 |
| *w*_s_ + s(*p_t_*_-1,s_) + *e_t_*_-1,s_ | 1\|*S* | 0.4725 | 0.5205 |
| *w*_s_ + *p_t_*_-1,s_ + *e_t_*_,s_ + *w*_s_ *p_t_*_-1,s_ + *w*_s_ *e_t_*_,s_ + *p_t_*_-1,s_ *e_t_*_,s_ | 1 + *e_t_*_,s_\|*S* | 0.4725 | 0.5227 |
| *w*_s_ + *p_t_*_-1,s_ + *e_t_*_-1,s_ | 1 + *p_t_*_-1,s_\|*S* | 0.4726 | 0.5248 |
| s(*w*_s_) + *p_t_*_-1,s_ + *e_t_*_-1,s_ | 1 + *p_t_*_-1,s_\|*S* | 0.4726 | 0.5269 |
| *w*_s_ + s(*p_t_*_-1,s_) + *e_t_*_-1,s_ | 1 + *p_t_*_-1,s_\|*S* | 0.4726 | 0.5291 |
| s(*w*_s_) + s(*p_t_*_-1,s_) + *e_t_*_-1,s_ | 1 + *p_t_*_-1,s_\|*S* | 0.4726 | 0.5312 |
| *w*_s_ + s(*p_t_*_-1,s_) + *e_t_*_-1,s_ | 1 + *e_t_*_-1,s_\|*S* | 0.4726 | 0.5333 |
| s(*w*_s_) + *p_t_*_-1,s_ + *e_t_*_-1,s_ | 1 + *e_t_*_-1,s_\|*S* | 0.4726 | 0.5354 |
| *w*_s_ + *p_t_*_-1,s_ + *e_t_*_-1,s_ | 1 + *e_t_*_-1,s_\|*S* | 0.4726 | 0.5376 |
| s(*w*_s_) + s(*p_t_*_-1,s_) + *e_t_*_-1,s_ | 1 + *e_t_*_-1,s_\|*S* | 0.4726 | 0.5397 |
| *w*_s_ + s(*p_t_*_-1,s_) + *e_t_*_-1,s_ | 0 + *w*_s_\|*S* | 0.4727 | 0.5418 |
| s(*w*_s_) + *p_t_*_-1,s_ + *e_t_*_-1,s_ | 0 + *w*_s_\|*S* | 0.4727 | 0.5439 |
| s(*w*_s_) + s(*p_t_*_-1,s_) + *e_t_*_-1,s_ | 0 + *w*_s_\|*S* | 0.4727 | 0.5460 |
| s(*w*_s_) + s(*p_t_*_-1,s_) + *e_t_*_-1,s_ | 1 + *w*_s_\|*S* | 0.4727 | 0.5481 |
| *w*_s_ + *p_t_*_-1,s_ + *e_t_*_-1,s_ | 0 + *w*_s_\|*S* | 0.4727 | 0.5503 |
| *w*_s_ + *p_t_*_-1,s_ + *e_t_*_,s_ + *w*_s_ *p_t_*_-1,s_ | 0 + *e_t_*_,s_\|*S* | 0.4727 | 0.5524 |
| *w*_s_ + s(*p_t_*_-1,s_) + *e_t_*_-1,s_ | 0 + *e_t_*_-1,s_\|*S* | 0.4727 | 0.5545 |
| *w*_s_ + *p_t_*_-1,s_ + *e_t_*_-1,s_ | 0 + *e_t_*_-1,s_\|*S* | 0.4727 | 0.5566 |
| s(*w*_s_) + *p_t_*_-1,s_ + *e_t_*_-1,s_ | 0 + *e_t_*_-1,s_\|*S* | 0.4727 | 0.5587 |
| s(*w*_s_) + s(*p_t_*_-1,s_) + *e_t_*_-1,s_ | 0 + *e_t_*_-1,s_\|*S* | 0.4727 | 0.5608 |
| *w*_s_ + s(*e_t_*_-1,s_) | 1\|*S* | 0.4727 | 0.5629 |
| s(*w*_s_) + s(*e_t_*_-1,s_) | 1\|*S* | 0.4727 | 0.5650 |
| *w*_s_ + *p_t_*_-1,s_ + *e_t_*_,s_ + *w*_s_ *p_t_*_-1,s_ | 0 + *w*_s_\|*S* | 0.4727 | 0.5671 |
| *w*_s_ + s(*e_t_*_-1,s_) | 0 + *w*_s_\|*S* | 0.4727 | 0.5692 |
| *w*_s_ + s(*e_t_*_-1,s_) | 1 + *w*_s_\|*S* | 0.4727 | 0.5713 |
| s(*w*_s_) + s(*e_t_*_-1,s_) | 0 + *w*_s_\|*S* | 0.4727 | 0.5734 |
| s(*w*_s_) + s(*e_t_*_-1,s_) | 1 + *w*_s_\|*S* | 0.4727 | 0.5755 |
| *w*_s_ + *p_t_*_-1,s_ + *e_t_*_,s_ | 0 + *p_t_*_-1,s_\|*S* | 0.4728 | 0.5776 |
| *w*_s_ + s(*p_t_*_-1,s_) + *e_t_*_-1,s_ | 0 + *p_t_*_-1,s_\|*S* | 0.4728 | 0.5797 |
| *w*_s_ + *p_t_*_,s_ + *e_t_*_-1,s_ + *p_t_*_,s_ *e_t_*_-1,s_ | 1 + *w*_s_\|*S* | 0.4728 | 0.5818 |
| s(ws) + *p_t_*_,s_ + *e_t_*_-1,s_ + *p_t_*_,s_ *e_t_*_-1,s_ | 1 + *w*_s_\|*S* | 0.4728 | 0.5839 |
| s(*w*_s_) + s(*e_t_*_-1,s_) | 1 + *e_t_*_-1,s_\|*S* | 0.4728 | 0.5860 |
| *w*_s_ + s(*e_t_*_-1,s_) | 1 + *e_t_*_-1,s_\|*S* | 0.4728 | 0.5881 |
| *w*_s_ + *p_t_*_-1,s_ + *e_t_*_,s_ + *p_t_*_-1,s_ *e_t_*_,s_ | 0 + *p_t_*_-1,s_\|*S* | 0.4728 | 0.5902 |
| *w*_s_ + *p_t_*_-1,s_ + *e_t_*_,s_ + *p_t_*_-1,s_ *e_t_*_,s_ | 1 + *w*_s_\|*S* | 0.4729 | 0.5922 |
| s(*w*_s_) + *p_t_*_-1,s_ + *e_t_*_,s_ + *p_t_*_-1,s_ *e_t_*_,s_ | 1 + *w*_s_\|*S* | 0.4729 | 0.5943 |
| *w*_s_ + *p_t_*_-1,s_ + *e_t_*_,s_ + *w*_s_ *p_t_*_-1,s_ | 1 + *e_t_*_,s_\|*S* | 0.4729 | 0.5964 |
| *w*_s_ + *p_t_*_,s_ + *e_t-1_*_,s_ + *p_t_*_,s_ *e_t-1_*_,s_ | 1\|*S* | 0.4729 | 0.5985 |
| s(*w*_s_) + *p_t_*_,s_ + *e_t_*_-1,s_ + *p_t_*_,s_ *e_t_*_-1,s_ | 1\|*S* | 0.4729 | 0.6005 |
| *w*_s_ + *p_t_*_,s_ + *e_t_*_-1,s_ + *p_t_*_,s_ *e_t_*_-1,s_ | 1 + *p_t_*_,s_\|*S* | 0.4729 | 0.6026 |
| s(*w*_s_) + *p_t_*_,s_ + *e_t_*_-1,s_ + *p_t_*_,s_ *e_t_*_-1,s_ | 1 + *p_t_*_,s_\|*S* | 0.4729 | 0.6047 |
| s(*w*_s_) + *p_t_*_-1,s_ + *e_t_*_,s_ + *p_t_*_-1,s_ *e_t_*_,s_ | 1\|*S* | 0.4730 | 0.6067 |
| *w*_s_ + *p_t_*_-1,s_ + *e_t_*_,s_ + *p_t_*_-1,s_ *e_t_*_,s_ | 1\|*S* | 0.4730 | 0.6088 |
| s(*w*_s_) + *p_t_*_,s_ + *e_t_*_-1,s_ + *p_t_*_,s_ *e_t_*_-1,s_ | 0 + *w*_s_\|*S* | 0.4730 | 0.6109 |
| *w*_s_ + *p_t_*_,s_ + *e_t_*_-1,s_ + *p_t_*_,s_ *e_t_*_-1,s_ | 0 + *w*_s_\|*S* | 0.4730 | 0.6129 |
| *w*_s_ + *p_t_*_-1,s_ + *e_t_*_,s_ | 1 + *w*_s_\|*S* | 0.4730 | 0.6150 |
| *w*_s_ + s(*p_t_*_-1,s_) + *e_t_*_,s_ | 1 + *w*_s_\|*S* | 0.4730 | 0.6170 |
| s(*w*_s_) + *p_t_*_-1,s_ + *e_t_*_,s_ | 1 + *w*_s_\|*S* | 0.4730 | 0.6191 |
| *w*_s_ + *p_t_*_-1,s_ + *e_t_*_,s_ | 1\|*S* | 0.4731 | 0.6211 |
| s(*w*_s_) + s(*p_t_*_-1,s_) + *e_t_*_,s_ | 1\|*S* | 0.4731 | 0.6232 |
| *w*_s_ + s(*p_t_*_-1,s_) + *e_t_*_,s_ | 1\|*S* | 0.4731 | 0.6252 |
| s(*ws*) + *p_t_*_-1,s_ + *e_t_*_,s_ + *p_t_*_,s_ *e_t_*_,s_ | 1\|*S* | 0.4731 | 0.6272 |
| *ws* + *p_t_*_-1,s_ + *e_t_*_,s_ + *p_t_*_,s_ *e_t_*_,s_ | 1 + *p_t_*_-1,s_\|*S* | 0.4731 | 0.6293 |
| s(*ws*) + *p_t_*_-1,s_ + *e_t_*_,s_ + *p_t_*_,s_ *e_t_*_,s_ | 1 + *p_t_*_-1,s_\|*S* | 0.4731 | 0.6313 |
| s(*ws*) + *p_t_*_-1,s_ + *e_t_*_,s_ + *p_t_*_,s_ *e_t_*_,s_ | 0 + *w*_s_\|*S* | 0.4731 | 0.6333 |
| *w*_s_ + *p_t_*_-1,s_ + *e_t_*_,s_ + *p_t_*_-1,s_ *e_t_*_,s_ | 0 + *w*_s_\|*S* | 0.4731 | 0.6354 |
| s(*w*_s_) + *p_t_*_,s_ + *e_t_*_,s_ + *p_t_*_,s_ *e_t_*_,s_ | 1 + *e_t_*_,s_\|*S* | 0.4732 | 0.6374 |
| *w*_s_ + s(*p_t_*_-1,s_) + *e_t_*_,s_ | 1 + *p_t_*_-1,s_\|*S* | 0.4732 | 0.6394 |
| s(*w*_s_) + *p_t_*_-1,s_ + *e_t_*_-1,s_ | 1 + *p_t_*_-1,s_\|*S* | 0.4732 | 0.6415 |
| *w*_s_ + *p_t_*_-1,s_ + *e_t_*_,s_ | 1 + *p_t_*_-1,s_\|*S* | 0.4732 | 0.6435 |
| s(*w*_s_) + s(*p_t_*_-1,s_) + *e_t_*_-,s_ | 1 + *p_t_*_-1,s_\|*S* | 0.4732 | 0.6455 |
| *w*_s_ + *p_t_*_,s_ + *e_t_*_-1,s_ + *p_t_*_,s_ *e_t_*_-1,s_ | 1 + *e_t_*_-1,s_\|*S* | 0.4732 | 0.6475 |
| s(*w*_s_) + *p_t_*_,s_ + *e_t_*_-1,s_ + *p_t_*_,s_ *e_t_*_-1,s_ | 1 + *e_t_*_-1,s_\|*S* | 0.4732 | 0.6495 |
| s(*w*_s_) + *p_t_*_-1,s_ | 0 + *p_t_*_-1,s_\|*S* | 0.4732 | 0.6516 |
| s(*w*_s_) + s(*p_t_*_-1,s_) | 0 + *p_t_*_-1,s_\|*S* | 0.4732 | 0.6536 |
| *w*_s_ + *p_t_*_,s_ + *e_t_*_-1,s_ + *p_t_*_,s_ *e_t_*_-1,s_ | 0 + *e_t_*_-1,s_\|*S* | 0.4732 | 0.6556 |
| s(*w*_s_) + *p_t_*_,s_ + *e_t_*_-1,s_ + *p_t_*_,s_ *e_t_*_-1,s_ | 0 + *e_t_*_-1,s_\|*S* | 0.4732 | 0.6576 |
| *w*_s_ + *p_t-1_*_,s_ + *e_t_*_,s_ + *p_t-1_*_,s_ *e_t_*_,s_ | 0 + *e_t_*_,s_\|*S* | 0.4733 | 0.6596 |
| s(*w*_s_) + *p_t-1_*_,s_ + *e_t_*_,s_ + *p_t-1_*_,s_ *e_t_*_,s_ | 0 + *e_t_*_,s_\|*S* | 0.4733 | 0.6616 |
| *w*_s_ + s(*e_t_*_-1,s_) | 0 + *e_t_*_-1,s_\|*S* | 0.4733 | 0.6637 |
| s(*w*_s_) + s(*e_t_*_-1,s_) | 0 + *e_t_*_-1,s_\|*S* | 0.4733 | 0.6657 |
| *w*_s_ + *p_t_*_-1,s_ + *e_t_*_,s_ | 0 + *w*_s_\|*S* | 0.4733 | 0.6677 |
| *w*_s_ + s(*p_t_*_-1,s_) + *e_t_*_,s_ | 0 + *w*_s_\|*S* | 0.4733 | 0.6697 |
| s(*w*_s_) + s(*p_t_*_-1,s_) + *e_t_*_,s_ | 0 + *w*_s_\|*S* | 0.4733 | 0.6717 |
| s(*w*_s_) + *p_t_*_-1,s_ + *e_t_*_,s_ | 0 + *w*_s_\|*S* | 0.4733 | 0.6737 |
| s(*w*_s_) + s(*p_t_*_-1,s_) + *e_t_*_,s_ | 1 + *w*_s_\|*S* | 0.4733 | 0.6757 |
| *w*_s_ + s(*p_t_*_-1,s_) + *e_t_*_,s_ | 0 + *e_t_*_,s_\|*S* | 0.4734 | 0.6777 |
| s(*w*_s_) + s(*p_t_*_-1,s_) + *e_t_*_,s_ | 0 + *e_t_*_,s_\|*S* | 0.4734 | 0.6797 |
| s(*w*_s_) + *p_t_*_-1,s_ + *e_t_*_,s_ | 0 + *e_t_*_,s_\|S | 0.4734 | 0.6817 |
| *w*_s_ + *p_t_*_-1,s_ + *e_t_*_,s_ | 0 + *e_t_*_,s_\|*S* | 0.4734 | 0.6837 |
| *w*_s_ + *p_t_*_,s_ + *e_t_*_,s_ + *w*_s_ *p_t_*_,s_ + *w*_s_ *e_t_*_,s_ + *p_t_*_,s_ *e_t_*_,s_ | 0 + *p_t_*_,s_\|*S* | 0.4734 | 0.6857 |
| *w*_s_ + *p_t_*_-1,s_ + *e_t_*_,s_ + *w*_s_ *p_t_*_,s_ | 0 + *p_t_*_,s_\|*S* | 0.4735 | 0.6877 |
| *w*_s_ + s(*p_t_*_,s_) + *e_t_*_,s_ | 0 + *p_t_*_,s_\|*S* | 0.4735 | 0.6896 |
| *w*_s_ + *p_t_*_,s_ + *e_t_*_,s_ | 0 + *p_t_*_,s_\|*S* | 0.4735 | 0.6916 |
| s(*w*_s_) + s(*e_t_*_,s_) | 1\|*S* | 0.4735 | 0.6936 |
| *w*_s_ + s(*e_t_*_,s_) | 1\|*S* | 0.4735 | 0.6956 |
| s(*w*_s_) + s(*e_t_*_,s_) | 0 + *w*_s_\|*S* | 0.4735 | 0.6975 |
| s(*w*_s_) + s(*e_t_*_,s_) | 1 + *w*_s_\|*S* | 0.4735 | 0.6995 |
| *w*_s_ + s(*e_t_*_,s_) | 0 + *w*_s_\|*S* | 0.4735 | 0.7015 |
| *w*_s_ + s(*e_t_*_,s_) | 1 + *w*_s_\|*S* | 0.4735 | 0.7035 |
| *w*_s_ + *p_t_*_-1,s_ + *e_t_*_,s_ | 1 + *e_t_*_,s_\|*S* | 0.4735 | 0.7054 |
| *w*_s_ + s(*p_t_*_-1,s_) + *e_t_*_,s_ | 1 + *e_t_*_,s_\|*S* | 0.4735 | 0.7074 |
| *w*_s_ + *p_t-1_*_,s_ + *e_t_*_,s_ + *p_t-1_*_,s_ *e_t_*_,s_ | 1 + *e_t_*_,s_\|*S* | 0.4736 | 0.7094 |
| *w*_s_ + *p_t_*_,s_ + *e_t_*_,s_ + *p_t_*_,s_ *e_t_*_,s_ | 0 + *p_t_*_,s_\|*S* | 0.4736 | 0.7113 |
| *w*_s_ + *p_t_*_,s_ + *e_t_*_,s_ + *w*_s_ *p_t_*_,s_ + *w*_s_ *e_t_*_,s_ + *p_t_*_,s_ *e_t_*_,s_ | 0 + *e_t_*_,s_\|*S* | 0.4736 | 0.7133 |
| *w*_s_ + *p_t_*_,s_ + *e_t_*_,s_ + *w*_s_ *p_t_*_,s_ | 1 + *w*_s_\|*S* | 0.4737 | 0.7152 |
| *w*_s_ + s(*p_t_*_,s_) + *e_t_*_,s_ | 1 + *w*_s_\|*S* | 0.4738 | 0.7171 |
| s(*w*_s_) + *p_t_*_,s_ + *e_t_*_,s_ | 1 + *w*_s_\|*S* | 0.4738 | 0.7191 |
| *w*_s_ + *p_t_*_,s_ + *e_t_*_,s_ | 1 + *w*_s_\|*S* | 0.4738 | 0.7210 |
| *w*_s_ + *p_t_*_,s_ + *e_t_*_,s_ + *w*_s_ *p_t_*_,s_ | 1 + *p_t_*_,s_\|*S* | 0.4738 | 0.7229 |
| *w*_s_ + s(*p_t_*_,s_) + *e_t_*_-1,s_ | 0 + *p_t_*_,s_\|*S* | 0.4738 | 0.7249 |
| *w*_s_ + *p_t_*_-1,s_ + *e_t_*_-1,s_ | 0 + *p_t_*_,s_\|*S* | 0.4738 | 0.7268 |
| *w*_s_ + *p_t_*_,s_ + *e_t_*_,s_ + *w*_s_ *p_t_*_,s_ | 1\|*S* | 0.4738 | 0.7287 |
| *w*_s_ + *p_t_*_,s_ + *e_t_*_,s_ | 1 + *p_t_*_,s_\|*S* | 0.4738 | 0.7306 |
| s(*w*_s_) + *p_t_*_,s_ + *e_t_*_,s_ | 1 + *p_t_*_,s_\|*S* | 0.4738 | 0.7326 |
| *w*_s_ + s(*p_t_*_,s_) + *e_t_*_,s_ | 1 + *p_t_*_,s_\|*S* | 0.4738 | 0.7345 |
| s(*w*_s_) + s(*p_t_*_,s_) + *e_t_*_,s_ | 1 + *p_t_*_,s_\|*S* | 0.4738 | 0.7364 |
| s(*w*_s_) + s(*p_t_*_,s_) + *e_t_*_,s_ | 1\|*S* | 0.4738 | 0.7383 |
| s(*w*_s_) + *p_t_*_,s_ + *e_t_*_,s_ | 1\|*S* | 0.4738 | 0.7402 |
| *w*_s_ + *p_t_*_,s_ + *e_t_*_,s_ | 1\|*S* | 0.4738 | 0.7422 |
| *w*_s_ + s(*p_t_*_,s_) + *e_t_*_,s_ | 1\|*S* | 0.4738 | 0.7441 |
| *w*_s_ + s(*e_t_*_,s_) | 1 + *e_t_*_,s_\|*S* | 0.4738 | 0.7460 |
| s(*w*_s_) + s(*e_t_*_,s_) | 1 + *e_t_*_,s_\|*S* | 0.4738 | 0.7479 |
| *w*_s_ + *p_t_*_,s_ + *e_t-1_*_,s_ + *w*_s_ *p_t_*_,s_ | 0 + *p_t_*_,s_\|*S* | 0.4738 | 0.7498 |
| *w*_s_ + *p_t_*_,s_ + *e_t_*_,s_ + *p_t_*_,s_ *e_t_*_,s_ | 1 + *w*_s_\|*S* | 0.4739 | 0.7517 |
| s(*w*_s_) + *p_t_*_,s_ + *e_t_*_,s_ + *p_t_*_,s_ *e_t_*_,s_ | 1 + *w*_s_\|*S* | 0.4739 | 0.7536 |
| *w*_s_ + *p_t_*_,s_ + *e_t_*_,s_ + *w*_s_ *p_t_*_,s_ | 0 + *w*_s_\|*S* | 0.4740 | 0.7555 |
| *w*_s_ + *p_t_*_,s_ + *e_t_*_,s_ + *w*_s_ *p_t_*_,s_ + *w*_s_ *e_t_*_,s_ + *p_t_*_,s_ *e_t_*_,s_ | 1\|*S* | 0.4740 | 0.7574 |
| *w*_s_ + *p_t_*_,s_ + *e_t_*_,s_ + *w*_s_ *p_t_*_,s_ + *w*_s_ *e_t_*_,s_ + *p_t_*_,s_ *e_t_*_,s_ | 1 + *p_t_*_,s_\|*S* | 0.4740 | 0.7593 |
| *w*_s_ + s(*p_t_*_,s_) + *e_t_*_,s_ | 0 + *w*_s_\|*S* | 0.4740 | 0.7612 |
| s(*w*_s_) + s(*p_t_*_,s_) + *e_t_*_,s_ | 1 + *w*_s_\|*S* | 0.4740 | 0.7631 |
| s(*w*_s_) + *p_t_*_,s_ + *e_t_*_,s_ | 0 + *w*_s_\|*S* | 0.4740 | 0.7650 |
| *w*_s_ + *p_t_*_,s_ + *e_t_*_,s_ | 0 + *w*_s_\|*S* | 0.4740 | 0.7669 |
| s(*w*_s_) + s(*p_t_*_,s_) + *e_t_*_,s_ | 0 + *w*_s_\|*S* | 0.4740 | 0.7688 |
| *w*_s_ + *p_t_*_,s_ + *e_t_*_,s_ + *w*_s_ *p_t_*_,s_ + *w*_s_ *e_t_*_,s_ + *p_t_*_,s_ *e_t_*_,s_ | 1 + *w*_s_\|*S* | 0.4740 | 0.7707 |
| s(*w*_s_) + *p_t_*_,s_ + *e_t_*_,s_ + *p_t_*_,s_ *e_t_*_,s_ | 1\|*S* | 0.4740 | 0.7726 |
| *w*_s_ + *p_t_*_,s_ + *e_t_*_,s_ + *p_t_*_,s_ *e_t_*_,s_ | 1\|*S* | 0.4740 | 0.7745 |
| *w*_s_ + *p_t_*_,s_ + *e_t_*_,s_ + *p_t_*_,s_ *e_t_*_,s_ | 1 + *p_t_*_,s_\|*S* | 0.4740 | 0.7763 |
| s(*w*_s_) + *p_t_*_,s_ + *e_t_*_,s_ + *p_t_*_,s_ *e_t_*_,s_ | 1 + *p_t_*_,s_\|*S* | 0.4740 | 0.7782 |
| *w*_s_ + *p_t_*_,s_ + *e_t_*_,s_ + *w*_s_ *p_t_*_,s_ | 0 + *e_t_*_,s_\|*S* | 0.4741 | 0.7801 |
| *w*_s_ + s(*e_t_*_,s_) | 0 + *e_t_*_,s_\|*S* | 0.4741 | 0.7820 |
| s(*w*_s_) + s(*e_t_*_,s_) | 0 + *e_t_*_,s_\|*S* | 0.4741 | 0.7839 |
| *w*_s_ + *p_t_*_,s_ + *e_t-1_*_,s_ + *w*_s_ *p_t_*_,s_ | 1 + *w*_s_\|*S* | 0.4741 | 0.7858 |
| s(*w*_s_) + *p_t_*_,s_ + *e_t_*_-1,s_ | 1 + *w*_s_\|*S* | 0.4741 | 0.7876 |
| *w*_s_ + s(*p_t_*_,s_) + *e_t_*_-1,s_ | 1 + *w*_s_\|*S* | 0.4741 | 0.7895 |
| *w*_s_ + *p_t_*_,s_ + *e_t_*_-1,s_ | 1 + *w*_s_\|*S* | 0.4741 | 0.7914 |
| s(*w*_s_) + s(*p_t_*_,s_) + *e_t_*_,s_ | 0 + *e_t_*_,s_\|*S* | 0.4741 | 0.7933 |
| s(*w*_s_) + *p_t_*_,s_ + *e_t_*_,s_ | 0 + *e_t_*_,s_\|*S* | 0.4741 | 0.7951 |
| *w*_s_ + *p_t_*_,s_ + *e_t_*_,s_ | 0 + *e_t_*_,s_\|*S* | 0.4741 | 0.7970 |
| *w*_s_ + s(*p_t_*_,s_) + *e_t_*_,s_ | 0 + *e_t_*_,s_\|*S* | 0.4741 | 0.7989 |
| *w*_s_ + *p_t_*_,s_ + *e_t_*_,s_ + *w*_s_ *p_t_*_,s_ | 1 + *p_t_*_,s_\|*S* | 0.4742 | 0.8008 |
| *w*_s_ + *p_t_*_,s_ + *e_t_*_,s_ + *w*_s_ *p_t_*_,s_ | 1\|*S* | 0.4742 | 0.8026 |
| s(*w*_s_) + *p_t_*_,s_ + *e_t_*_,s_ + *p_t_*_,s_ *e_t_*_,s_ | 0 + *w*_s_\|*S* | 0.4742 | 0.8045 |
| *w*_s_ + *p_t_*_,s_ + *e_t_*_,s_ + *p_t_*_,s_ *e_t_*_,s_ | 0 + *w*_s_\|*S* | 0.4742 | 0.8063 |
| *w*_s_ + *p_t_*_,s_ + *e_t_*_-1,s_ | 1 + *p_t_*_,s_\|*S* | 0.4742 | 0.8082 |
| s(*w*_s_) + s(*p_t_*_,s_) + *e_t_*_-1,s_ | 1 + *p_t_*_,s_\|*S* | 0.4742 | 0.8101 |
| s(*w*_s_) + *p_t_*_,s_ + *e_t_*_-1,s_ | 1 + *p_t_*_,s_\|*S* | 0.4742 | 0.8119 |
| *w*_s_ + s(*p_t_*_,s_) + *e_t_*_-1,s_ | 1 + *p_t_*_,s_\|*S* | 0.4742 | 0.8138 |
| *w*_s_ + s(*p_t_*_,s_) + *e_t_*_-1,s_ | 1\|*S* | 0.4742 | 0.8157 |
| s(*w*_s_) + *p_t_*_,s_ + *e_t_*_-1,s_ | 1\|*S* | 0.4742 | 0.8175 |
| *w*_s_ + *p_t_*_,s_ + *e_t_*_-1,s_ | 1\|*S* | 0.4742 | 0.8194 |
| s(*w*_s_) + s(*p_t_*_,s_) + *e_t_*_-1,s_ | 1\|*S* | 0.4742 | 0.8213 |
| s(*w*_s_) + *p_t_*_,s_ | 0 + *p_t_*_,s_\|*S* | 0.4742 | 0.8231 |
| s(*w*_s_) + s(*p_t_*_,s_) | 0 + *p_t_*_,s_\|*S* | 0.4742 | 0.8250 |
| *w*_s_ + *p_t_*_,s_ + *e_t_*_,s_ + *w*_s_ *p_t_*_,s_ | 1 + *e_t_*_,s_\|*S* | 0.4742 | 0.8268 |
| *w*_s_ + *p_t_*_,s_ + *e_t-1_*_,s_ + *w*_s_ *p_t_*_,s_ | 1 + *e_t_*_-1,s_\|*S* | 0.4743 | 0.8287 |
| *w*_s_ + *p_t_*_,s_ + *e_t_*_,s_ + *p_t_*_,s_ *e_t_*_,s_ | 0 + *e_t_*_,s_\|*S* | 0.4743 | 0.8305 |
| s(*w*_s_) + *p_t_*_,s_ + *e_t_*_,s_ + *p_t_*_,s_ *e_t_*_,s_ | 0 + *e_t_*_,s_\|*S* | 0.4743 | 0.8324 |
| s(*w*_s_) + *p_t_*_,s_ + *e_t_*_-1,s_ | 1 + *e_t_*_-1,s_\|*S* | 0.4743 | 0.8342 |
| *w*_s_ + s(*p_t_*_,s_) + *e_t_*_-1,s_ | 1 + *e_t_*_-1,s_\|*S* | 0.4743 | 0.8361 |
| *w*_s_ + *p_t_*_,s_ + *e_t_*_-1,s_ | 1 + *e_t_*_-1,s_\|*S* | 0.4743 | 0.8379 |
| s(*w*_s_) + s(*p_t_*_,s_) + *e_t_*_-1,s_ | 1 + *e_t_*_-1,s_\|*S* | 0.4743 | 0.8398 |
| *w*_s_ + *p_t_*_,s_ + *e_t_*_,s_ | 1 + *e_t_*_,s_\|*S* | 0.4743 | 0.8416 |
| *w*_s_ + s(*p_t_*_,s_) + *e_t_*_,s_ | 1 + *e_t_*_,s_\|*S* | 0.4743 | 0.8435 |
| *w*_s_ + *p_t_*_,s_ + *e_t_*_,s_ + *w*_s_ *p_t_*_,s_ + *w*_s_ *e_t_*_,s_ + *p_t_*_,s_ *e_t_*_,s_ | 0 + *w*_s_\|*S* | 0.4743 | 0.8453 |
| *w*_s_ + *p_t_*_,s_ + *e_t_*_-1,s_ + *p_t_*_,s_ *e_t_*_-1,s_ | 0 + *w*_s_\|*S* | 0.4743 | 0.8472 |
| s(*w*_s_) + s(*p_t_*_,s_) + *e_t_*_-1,s_ | 0 + *w*_s_\|*S* | 0.4743 | 0.8490 |
| *w*_s_ + s(*p_t_*_,s_) + *e_t_*_-1,s_ | 0 + *w*_s_\|*S* | 0.4743 | 0.8509 |
| *w*_s_ + *p_t_*_,s_ + *e_t_*_-1,s_ | 0 + *w*_s_\|*S* | 0.4743 | 0.8527 |
| s(*w*_s_) + *p_t_*_,s_ + *e_t_*_-1,s_ | 0 + *w*_s_\|*S* | 0.4743 | 0.8545 |
| s(*w*_s_) + s(*p_t_*_,s_) + *e_t_*_-1,s_ | 1 + *w*_s_\|*S* | 0.4743 | 0.8564 |
| *w*_s_ + *p_t_*_,s_ + *e_t_*_-1,s_ + *w*_s_ *p_t_*_,s_ | 0 + *e_t_*_-1,s_\|*S* | 0.4744 | 0.8582 |
| s(*w*_s_) + *p_t_*_,s_ + *e_t_*_-1,s_ | 0 + *e_t_*_-1,s_\|*S* | 0.4744 | 0.8601 |
| *w*_s_ + s(*p_t_*_,s_) + *e_t_*_-1,s_ | 0 + *e_t_*_-1,s_\|*S* | 0.4744 | 0.8619 |
| *w*_s_ + *p_t_*_,s_ + *e_t_*_-1,s_ | 0 + *e_t_*_-1,s_\|*S* | 0.4744 | 0.8637 |
| s(*w*_s_) + s(*p_t_*_,s_) + *e_t_*_-1,s_ | 0 + *e_t_*_-1,s_\|*S* | 0.4744 | 0.8655 |
| *w*_s_ + *p_t_*_,s_ + *e_t_*_,s_ + *w*_s_ *p_t_*_,s_ + *w*_s_ *e_t_*_,s_ + *p_t_*_,s_ *e_t_*_,s_ | 1 + *e_t_*_,s_\|*S* | 0.4745 | 0.8674 |
| *w*_s_ + *p_t_*_,s_ + *e_t_*_,s_ + *p_t_*_,s_ *e_t_*_,s_ | 1 + *e_t_*_,s_\|*S* | 0.4748 | 0.8691 |
| *w*_s_ + *e_t_*_-1,s_ + *w*_s_ *e_t_*_-1,s_ | 0 + *e_t_*_-1,s_\|*S* | 0.4752 | 0.8708 |
| *w*_s_ + *e_t_*_-1,s_ | 1 + *w*_s_\|*S* | 0.4753 | 0.8725 |
| *w*_s_ + *e_t_*_-1,s_ + *w*_s_ *e_t_*_-1,s_ | 1 + *w*_s_\|*S* | 0.4754 | 0.8742 |
| *w*_s_ + *e_t_*_-1,s_ + *w*_s_ *e_t_*_-1,s_ | 1\|*S* | 0.4754 | 0.8759 |
| *w*_s_ + *e_t_*_-1,s_ | 1\|*S* | 0.4754 | 0.8776 |
| s(*w*_s_) + *e_t_*_-1,s_ | 1\|*S* | 0.4754 | 0.8793 |
| *w*_s_ + *e_t_*_-1,s_ + *w*_s_ *e_t_*_-1,s_ | 1 + *e_t_*_-1,s_\|*S* | 0.4755 | 0.8810 |
| *w*_s_ + *e_t_*_-1,s_ | 0 + *w*_s_\|*S* | 0.4755 | 0.8826 |
| s(*w*_s_) + *e_t_*_-1,s_ | 1 + *w*_s_\|*S* | 0.4755 | 0.8843 |
| *w*_s_ + *e_t_*_-1,s_ | 0 + *w*_s_\|*S* | 0.4755 | 0.8860 |
| s(*w*_s_) + *e_t_*_-1,s_ | 1 + *e_t_*_-1,s_\|*S* | 0.4755 | 0.8877 |
| *w*_s_ + *e_t_*_-1,s_ | 1 + *e_t_*_-1,s_\|*S* | 0.4755 | 0.8893 |
| *w*_s_ + *e_t_*_-1,s_ + *w*_s_ *e_t_*_-1,s_ | 0 + *w*_s_\|*S* | 0.4756 | 0.8910 |
| *w*_s_ + *e_t_*_-1,s_ | 0 + *e_t_*_-1,s_\|*S* | 0.4757 | 0.8926 |
| s(*w*_s_) + *e_t_*_-1,s_ | 0 + *e_t_*_-1,s_\|*S* | 0.4757 | 0.8943 |
| *w*_s_ + *p_t_*_-1,s_ + *w*_s_ *p_t_*_-1,s_ | 0 + *p_t_*_-1,s_\|*S* | 0.4757 | 0.8959 |
| *w*_s_ + *e_t_*_,s_ + *w*_s_ *e_t_*_,s_ | 0 + *e_t_*_,s_\|*S* | 0.4758 | 0.8976 |
| *w*_s_ + *e_t_*_,s_ + *w*_s_ *e_t_*_,s_ | 1 + *w*_s_\|*S* | 0.4759 | 0.8992 |
| *w*_s_ + *e_t_*_,s_ + *w*_s_ *e_t_*_,s_ | 1\|*S* | 0.4760 | 0.9008 |
| *w*_s_ + *e_t_*_,s_ | 1 + *w*_s_\|*S* | 0.4760 | 0.9024 |
| *w*_s_ + *e_t_*_,s_ + *w*_s_ *e_t_*_,s_ | 0 + *w*_s_\|*S* | 0.4761 | 0.9040 |
| *w*_s_ + *p_t-1_*_,s_ + *w*_s_ *p_t-1_*_,s_ | 1\|*S* | 0.4761 | 0.9056 |
| *w*_s_ + *p_t-1_*_,s_ + *w*_s_ *p_t-1_*_,s_ | 1 + *w*_s_\|*S* | 0.4761 | 0.9072 |
| *w*_s_ + *p_t-1_*_,s_ + *w*_s_ *p_t-1_*_,s_ | 1 + *p_t_*_-1,s_\|*S* | 0.4761 | 0.9088 |
| *w*_s_ + *e_t_*_,s_ | 1\|*S* | 0.4761 | 0.9104 |
| s(*w*_s_) + *e_t_*_,s_ | 1\|*S* | 0.4761 | 0.9120 |
| *w*_s_ + *e_t_*_,s_ | 0 + *w*_s_\|*S* | 0.4761 | 0.9136 |
| s(*w*_s_) + *e_t_*_,s_ | 1 + *w*_s_\|*S* | 0.4761 | 0.9151 |
| s(*w*_s_) + *e_t_*_,s_ | 0 + *w*_s_\|*S* | 0.4761 | 0.9167 |
| *w*_s_ + *e_t_*_,s_ + *w*_s_ *e_t_*_,s_ | 1 + *e_t_*_,s_\|*S* | 0.4762 | 0.9183 |
| *w*_s_ + *p_t-1_*_,s_ + *w*_s_ *p_t-1_*_,s_ | 0 + *w*_s_\|*S* | 0.4764 | 0.9199 |
| *w*_s_ + *e_t_*_,s_ | 1 + *e_t_*_,s_\|*S* | 0.4764 | 0.9214 |
| s(*w*_s_) + *e_t_*_,s_ | 1 + *e_t_*_,s_\|*S* | 0.4764 | 0.9230 |
| *w*_s_ + s(*p_t_*_-1,s_) | 0 + *p_t_*_-1,s_\|*S* | 0.4764 | 0.9245 |
| *w*_s_ + *p_t_*_-1,s_ | 0 + *p_t_*_-1,s_\|*S* | 0.4764 | 0.9261 |
| s(*w*_s_) + *e_t_*_,s_ | 0 + *e_t_*_,s_\|*S* | 0.4764 | 0.9276 |
| *w*_s_ + *e_t_*_,s_ | 0 + *e_t_*_,s_\|*S* | 0.4764 | 0.9292 |
| s(*w*_s_) + *p_t_*_-1,s_ | 1 + *w*_s_\|*S* | 0.4766 | 0.9307 |
| *w*_s_ + s(*p_t_*_-1,s_) | 1 + *w*_s_\|*S* | 0.4766 | 0.9322 |
| s(*w*_s_) + s(*p_t_*_-1,s_) | 1 + *w*_s_\|*S* | 0.4766 | 0.9337 |
| s(*w*_s_) + s(*p_t_*_-1,s_) | 1\|*S* | 0.4767 | 0.9353 |
| *w*_s_ + *p_t_*_-1,s_ | 1\|*S* | 0.4767 | 0.9368 |
| s(*w*_s_) + *p_t_*_-1,s_ | 1\|*S* | 0.4767 | 0.9383 |
| *w*_s_ + s(*p_t_*_-1,s_) | 1\|*S* | 0.4767 | 0.9398 |
| *w*_s_ + s(*p_t_*_-1,s_) | 1 + *p_t_*_-1,s_\|*S* | 0.4768 | 0.9413 |
| *w*_s_ + *p_t_*_-1,s_ | 1 + *p_t_*_-1,s_\|*S* | 0.4768 | 0.9428 |
| s(*w*_s_) + *p_t_*_-1,s_ | 1 + *p_t_*_-1,s_\|*S* | 0.4768 | 0.9443 |
| s(*w*_s_) + s(*p_t_*_-1,s_) | 1 + *p_t_*_-1,s_\|*S* | 0.4768 | 0.9458 |
| *w*_s_ + *p_t_*_-1,s_ | 0 + *w*_s_\|*S* | 0.4769 | 0.9473 |
| *w*_s_ + *p_t_*_-1,s_ | 1 + *w*_s_\|*S* | 0.4769 | 0.9488 |

**Table S5.** Best predictive models used in the construction of the number of recruits average model, where *w* refers to soil water content at saturation, *p_t_* to the Palmer Drought Severity index at time *t* , *p_t_*_-1_ Palmer Drought Severity index at time *t*-1, *e_t_* to the El Niño-Southern Oscillation (ENSO) at time *t*, *e_t_*_-1_ to the El Niño-Southern Oscillation (ENSO) at time *t*-1, and *S* to the random effect of Site. For each variable, subscript s refers to standardization, and function s() refers to a spline (i.e., non-linear model).

| **Model** | | **MRSME** | **Cumulative MRSME weight** |
| --- | --- | --- | --- |
| **Fixed effect** | **Random effect** |  |  |
| s(*w*_,s_) + *p_t_*_,s_ + s(*e_t_*_,s_) | 0 + *p_t_*_,s_\|*S* | 17.8357 | 0.0961 |
| s(*w*_,s_) + *p_t_*_,s_ + s(*e_t_*_,s_) | 0 + *w*_,s_\|*S* | 17.8357 | 0.1921 |
| s(*w*_,s_) + *p_t_*_,s_ + *e_t_*_,s_ + *p_t_*_,s_ *e_t_*_,s_ | 0 + *p_t_*_,s_\|*S* | 17.8580 | 0.2382 |
| s(*w*_,s_) + *p_t_*_,s_ + *e_t_*_,s_ + *p_t_*_,s_ *e_t_*_,s_ | 1\|*S* | 17.8582 | 0.2839 |
| s(*w*_,s_) + *p_t_*_,s_ + *e_t_*_,s_ | 0 + *p_t_*_,s_\|*S* | 17.8587 | 0.3289 |
| s(*w*_,s_) + s(*p_t_*_,s_) + *e_t_*_,s_ | 0 + *w*_,s_\|*S* | 17.8587 | 0.3739 |
| s(*w*_,s_) + s(*p_t_*_,s_) + *e_t_*_,s_ | 0 + *p_t_*_,s_\|*S* | 17.8587 | 0.4189 |
| s(*w*_,s_) + s(*p_t_*_,s_) + *e_t_*_,s_ | 1 + *p_t_*_,s_\|*S* | 17.8587 | 0.4639 |
| s(*w*_,s_) + s(*p_t_*_,s_) + *e_t_*_,s_ | 0 + *e_t_*_,s_\|*S* | 17.8587 | 0.5088 |
| s(*w*_,s_) + s(*p_t_*_,s_) + s(*e_t_*_,s_) | 0 + *e_t_*_,s_\|*S* | 17.8587 | 0.5538 |
| s(*w*_,s_) + s(*p_t_*_,s_) + s(*e_t_*_,s_) | 0 + *w*_,s_\|*S* | 17.8587 | 0.5988 |
| s(*w*_,s_) + s(*p_t_*_,s_) + s(*e_t_*_,s_) | 0 + *p_t_*_,s_\|*S* | 17.8587 | 0.6438 |
| s(*w*_,s_) + s(*p_t_*_-1,s_) + s(*e_t_*_,s_) | 0 + *w*_,s_\|*S* | 17.8663 | 0.6787 |
| s(*w*_,s_) + *p_t_*_-1,s_ + s(*e_t_*_,s_) | 0 + *w*_,s_\|*S* | 17.8663 | 0.7136 |
| s(*w*_,s_) + s(*p_t_*_-1,s_) + s(*e_t_*_,s_) | 0 + *e_t_*_,s_\|*S* | 17.8663 | 0.7485 |
| s(*w*_,s_) + *p_t_*_-1,s_ + *e_t_*_,s_ + *p_t_*_-1,s_ *e_t_*_,s_ | 0 + *e_t_*_,s_\|*S* | 17.8832 | 0.7686 |
| s(*w*_,s_) + *p_t_*_-1,s_ + *e_t_*_,s_ + *p_t_*_-1,s_ *e_t_*_,s_ | 0 + *p_t_*_-1,s_\|*S* | 17.8832 | 0.7886 |
| s(*w*_,s_) + s(*p_t_*_-1,s_) + *e_t_*_,s_ | 0 + *p_t_*_-1,s_\|*S* | 17.8837 | 0.8083 |
| s(*w*_,s_) + *p_t_*_-1,s_ + s(*e_t_*_,s_) | 0 + *p_t_*_-1,s_\|*S* | 17.8837 | 0.8280 |
| s(*w*_,s_) + s(*p_t_*_-1,s_) + *e_t_*_,s_ | 0 + *w*_,s_\|*S* | 17.8837 | 0.8477 |
| s(*w*_,s_) + *p_t_*_-1,s_ + *e_t_*_,s_ | 0 + *p_t_*_-1,s_\|*S* | 17.8837 | 0.8674 |
| *w*_,s_ + *p_t_*_,s_ + *e_t_*_,s_ + *w*_,s_ *p_t_*_,s_ + *w*_,s_ *e_t_*_,s_ + *p_t_*_,s_ *e_t_*_,s_ | 0 + *p_t_*_,s_\|*S* | 17.9094 | 0.8758 |
| *w*_,s_ + *p_t_*_,s_ + *e_t_*_,s_ + *w*_,s_ *p_t_*_,s_ + *w*_,s_ *e_t_*_,s_ + *p_t_*_,s_ *e_t_*_,s_ | 0 + *e_t_*_,s_\|*S* | 17.9094 | 0.8843 |
| *w*_,s_ + *p_t_*_,s_ + *e_t_*_,s_ + *w*_,s_ *p_t_*_,s_ + *w*_,s_ *e_t_*_,s_ + *p_t_*_,s_ *e_t_*_,s_ | 1\|*S* | 17.9094 | 0.8927 |
| *w*_,s_ + *p_t_*_,s_ + *e_t_*_,s_ + *w*_,s_ *p_t_*_,s_ + *w*_,s_ *e_t_*_,s_ + *p_t_*_,s_ *e_t_*_,s_ | 0 + *w*_,s_\|*S* | 17.9094 | 0.9012 |
| *w*_,s_ + *p_t_*_,s_ + s(*e_t_*_,s_) + *w*_,s_ *p_t_*_,s_ | 0 + *p_t_*_,s_\|*S* | 17.9110 | 0.9092 |
| s(*w*_,s_) + *p_t_*_-1,s_ + s(*e_t_*_,s_) | 1 + *p_t_*_-1,s_\|*S* | 17.9133 | 0.9166 |
| *w*_,s_ + *p_t_*_,s_ + *e_t_*_,s_ + *w*_,s_ *p_t_*_,s_ | 0 + *e_t_*_,s_\|*S* | 17.9249 | 0.9217 |
| *w*_,s_ + *p_t_*_,s_ + s(*e_t_*_,s_) + *w*_,s_ *p_t_*_,s_ | 1\|*S* | 17.9249 | 0.9267 |
| *w*_,s_ + *p_t_*_,s_ + *e_t_*_,s_ + *w*_,s_ *p_t_*_,s_ | 0 + *p_t_*_,s_\|*S* | 17.9249 | 0.9318 |
| *w*_,s_ + *p_t_*_,s_ + s(*e_t_*_,s_) + *w*_,s_ *p_t_*_,s_ | 0 + *e_t_*_,s_\|*S* | 17.9249 | 0.9368 |
| *w*_,s_ + *p_t_*_,s_ + *e_t_*_,s_ + *w*_,s_ *p_t_*_,s_ | 0 + *w*_,s_\|*S* | 17.9249 | 0.9419 |
| *w*_,s_ + *p_t_*_,s_ + *e_t_*_,s_ + *w*_,s_ *p_t_*_,s_ | 1\|*S* | 17.9249 | 0.9470 |

**Table S6:** Statistics (geometric mean, standard deviation, and slope over time) of the population growth rate (ind.∙y^-1^) time series projected for 13 saguaro populations from 2017 to 2099 under two climate change scenarios.

| **Population** | Climate change scenario | | | | | | |
| --- | --- | --- | --- | --- | --- | --- | --- |
|  | Very low emissions | | |  | Very high emissions | | |
|  | **Mean** | **SD** | **Slope** |  | **Mean** | **SD** | **Slope** |
| Joyita | 0.940 | 0.155 | 1.96⋅10^-3^ |  | 0.933 | 0.135 | 1.18⋅10^-3^ |
| Vidrios | 0.947 | 0.059 | 1.02⋅10^-3^ |  | 0.943 | 0.044 | 2.96⋅10^-4^ |
| McDougal | 0.936 | 0.112 | 2.04⋅10^-3^ |  | 0.929 | 0.094 | 1.20⋅10^-3^ |
| Primavera | 0.962 | 0.039 | 4.47⋅10^-4^ |  | 0.959 | 0.032 | -8.53⋅10^-6^ |
| Caborca | 0.953 | 0.070 | 8.01⋅10^-4^ |  | 0.948 | 0.048 | 5.89⋅10^-5^ |
| Cucurpe | 0.965 | 0.042 | 2.04⋅10^-4^ |  | 0.961 | 0.033 | -3.24⋅10^-4^ |
| Dipo | 0.958 | 0.063 | 6.89⋅10^-4^ |  | 0.953 | 0.046 | 9.83⋅10^-5^ |
| Lobos | 0.938 | 0.193 | 2.19⋅10^-3^ |  | 0.930 | 0.152 | 1.28⋅10^-3^ |
| Orégano | 0.958 | 0.060 | 5.38⋅10^-4^ |  | 0.953 | 0.044 | -1.84⋅10^-4^ |
| Bahía Kino | 0.950 | 0.077 | 9.81⋅10^-4^ |  | 0.944 | 0.048 | 1.33⋅10^-4^ |
| San Marcial | 0.943 | 0.114 | 1.42⋅10^-3^ |  | 0.938 | 0.069 | 6.00.10-4 |
| Guásimas | 0.945 | 0.073 | 1.07⋅10^-3^ |  | 0.940 | 0.047 | 3.06⋅10^-4^ |
| Masiaca | 0.946 | 0.114 | 8.01⋅10^-4^ |  | 0.941 | 0.083 | 5.33⋅10^-4^ |
